# Small RNA Sequencing Reveals *Dlk1-Dio3* Locus-Embedded MicroRNAs as Major Drivers of Ground-State Pluripotency

**DOI:** 10.1016/j.stemcr.2017.10.009

**Published:** 2017-11-09

**Authors:** Sharif Moradi, Ali Sharifi-Zarchi, Amirhossein Ahmadi, Sepideh Mollamohammadi, Alexander Stubenvoll, Stefan Günther, Ghasem Hosseini Salekdeh, Sassan Asgari, Thomas Braun, Hossein Baharvand

**Affiliations:** 1 Department of Stem Cells and Developmental Biology, Cell Science Research Center, Royan Institute for Stem Cell Biology and Technology, ACECR, Banihashem Square, Banihashem Street, Ressalat Highway, Tehran 1665659911, Iran; 2 Department of Developmental Biology, University of Science and Culture, Tehran, Iran; 3 Computer Engineering Department, Sharif University of Technology, Tehran, Iran; 4 Max-Planck Institute for Heart and Lung Research, Department of Cardiac Development and Remodelling, Ludwigstrasse 43, 61231 Bad Nauheim, Germany; 5 Australian Infectious Disease Research Centre, School of Biological Sciences, The University of Queensland, Brisbane, QLD, Australia

**Keywords:** *Dlk1-Dio3* locus, ground-state pluripotency, microRNA, small RNA sequencing, differentiation, self-renewal, *Sfmbt2* locus

## Abstract

Ground-state pluripotency is a cell state in which pluripotency is established and maintained through efficient repression of endogenous differentiation pathways. Self-renewal and pluripotency of embryonic stem cells (ESCs) are influenced by ESC-associated microRNAs (miRNAs). Here, we provide a comprehensive assessment of the “miRNome” of ESCs cultured under conditions favoring ground-state pluripotency. We found that ground-state ESCs express a distinct set of miRNAs compared with ESCs grown in serum. Interestingly, most “ground-state miRNAs” are encoded by an imprinted region on chromosome 12 within the *Dlk1-Dio3* locus. Functional analysis revealed that ground-state miRNAs embedded in the *Dlk1-Dio3* locus (miR-541-5p, miR-410-3p, and miR-381-3p) promoted pluripotency via inhibition of multi-lineage differentiation and stimulation of self-renewal. Overall, our results demonstrate that ground-state pluripotency is associated with a unique miRNA signature, which supports ground-state self-renewal by suppressing differentiation.

## Highlights

• Ground-state pluripotency is associated with a unique miRNA signature
• *Dlk1-Dio3* locus on 12qF1 encodes the majority of ground-state miRNAs
• *Dlk1-Dio3* locus is hypomethylated in ground-state ESCs
• Ground-state miRNAs promote ESC self-renewal and inhibit differentiation

## Graphical Abstract

**Figure:**
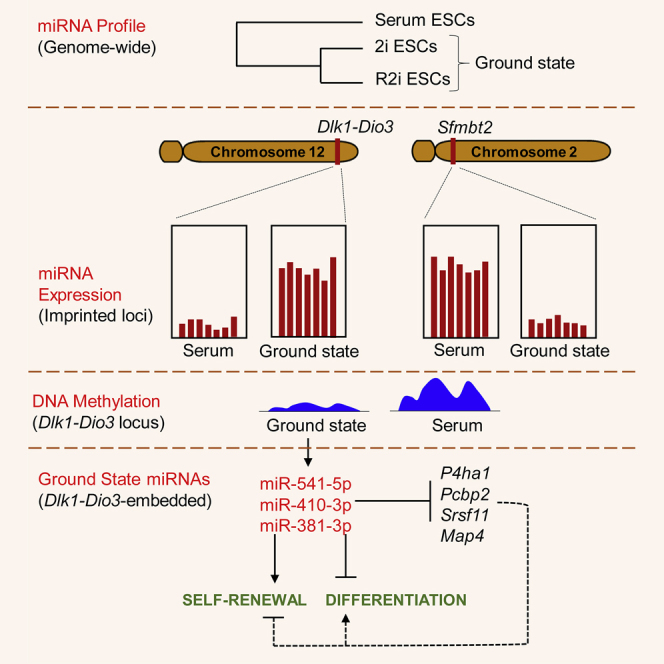

## Teaser

Ground-state pluripotency is a cell state in which pluripotency is maintained through inhibition of differentiation. In this paper, Baharvand and colleagues report that ground-state pluripotency is associated with a unique microRNA signature. They find that ground-state microRNAs, which are mostly encoded by the *Dlk1-Dio3* locus, contribute to the maintenance of ESCs through stimulating self-renewal and inhibiting differentiation.

## Introduction

Embryonic stem cells (ESCs) are derived from the inner cell mass of blastocyst-stage embryo and provide a perpetual cell source to investigate pluripotency and stem cell self-renewal *in vitro* (Evans and Kaufman, 1981, Hassani et al., 2014a, Martin, 1981). ESCs were originally derived and maintained in serum-containing media on feeder cells (Evans and Kaufman, 1981, Martin, 1981). Further studies revealed that feeder cells provide leukemia inhibitory factor (LIF) whereas serum provides bone morphogenetic protein (BMP) signals, which inhibit ESC differentiation into mesendoderm and neuroectoderm, respectively (Ying et al., 2003). Based on these findings, ESC cultures supplemented with BMP and LIF signals have been used to maintain ESCs in an undifferentiated state and to suppress endogenous differentiation-promoting signals (Ying et al., 2003). Notably, pharmacological inhibition of endogenous pro-differentiation ESC signals allows maintenance and establishment of ESCs from different mouse and rat strains. Such culture conditions are defined as 2i, whereby two small-molecule inhibitors are used to block the glycogen synthase kinase 3 (GSK3) and fibroblast growth factor-extracellular regulated kinase (FGF-ERK) pathways, allowing indefinite growth of ESCs without the need for exogenous signals. This so-called ground state of pluripotency displays robust pluripotency due to efficient repression of intrinsic differentiation signals and shows a remarkable homogeneity compared with ESCs kept in serum (Wray et al., 2010, Ying et al., 2008).

Recently, we devised alternative culture conditions, dubbed R2i, which allow ground-state cultivation and efficient generation of ESCs from pre-implantation embryos (Hassani et al., 2014b). R2i conditions feature inhibition of transforming growth factor β (TGF-β) and FGF-ERK signaling instead of GSK3 and FGF-ERK blockage used in the 2i approach. Compared with GSK3 inhibition, suppression of TGF-β signaling reduces genomic instability of ESCs and allows derivation of ESCs from single blastomeres at much higher efficiency (Hassani et al., 2014a, Hassani et al., 2014b). Since 2i and R2i ESCs both represent the ground state of ESC pluripotency, a systematic comparison of similarities and differences might aid in the understanding of core mechanisms underlying ground-state pluripotency.

MicroRNAs (miRNAs) are ∼22-nt long non-coding RNAs that post-transcriptionally regulate a large number of genes in mammalian cells, thereby modulating virtually all biological pathways including cell-fate decisions and reprogramming (Baek et al., 2008, Bartel, 2009, Moradi et al., 2014, Sayed and Abdellatif, 2011). In ESCs, ablation of miRNA-processing enzymes impairs self-renewal, rendering ESCs unable to differentiate (Kanellopoulou et al., 2005, Wang et al., 2007). Individual miRNAs play important roles in ESC regulation. miR-290–295 cluster or let-7 family members, for example, promote or impair ESC self-renewal, respectively (Melton et al., 2010). Moreover, miRNAs enriched in ESCs promote de-differentiation of somatic cells into induced pluripotent stem cells (iPSCs) (Moradi et al., 2014). So far, most studies have focused on the expression and functional significance of miRNAs in ESCs kept in serum (Graham et al., 2016, Hadjimichael et al., 2016, Houbaviy et al., 2003, Liu et al., 2014, Marson et al., 2008, Melton et al., 2010, Parchem et al., 2015, Tay et al., 2008, Wang et al., 2008), which leaves a critical gap about the functional importance of miRNAs in ESCs cultured in ground-state conditions despite many insights into the transcriptome, epigenome, and proteome of ground-state pluripotency (Habibi et al., 2013, Marks et al., 2012, Taleahmad et al., 2015).

In the present study, we analyzed the global expression patterns of miRNAs in ESCs cultured in ground-state conditions of 2i and R2i compared with serum using small RNA sequencing. We provide a comprehensive report on the “miRNome” of ground-state pluripotency compared with serum cells, which enabled us to identify miRNAs specific to each cell state. Furthermore, we found that selected ground-state miRNAs contribute to the maintenance of ground-state pluripotency by promoting self-renewal and repressing differentiation.

## Results

### Analysis of Small RNA Expression in Ground-State ESCs

**Figure 1:**
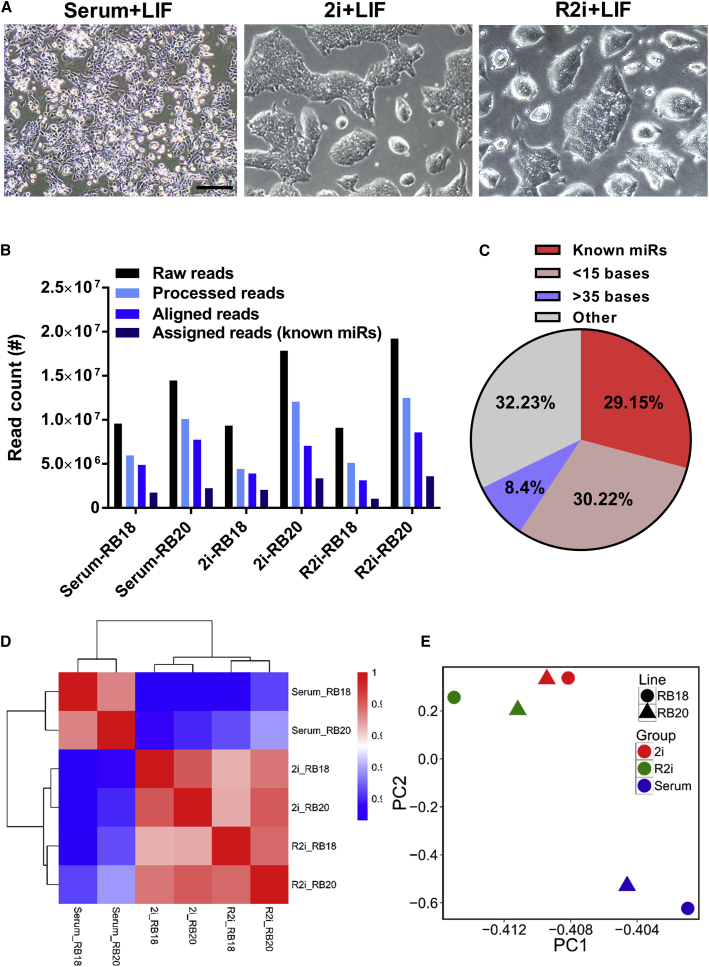
Small RNA Sequencing of ESCs Cultured under Serum, 2i, and R2i Conditions (A) Phase-contrast images of ESCs cultured under serum, 2i, and R2i. Scale bar, 200 μm. (B) Raw, processed, aligned, and assigned read counts of each ESC sample. Small RNA-sequencing data were obtained for two ESC lines (RB18 and EB20) each time using pools of three independently grown cultures (independent experiments). (C) Pie chart indicating the proportion of processed reads accounting for miRNAs. Small RNA profiles were obtained for RB18 and EB20 ESC lines using pools of three independently grown cultures (independent experiments). (D) Pearson heatmap of the ESC samples. Samples are shown as rows and columns. Each square represents the Pearson correlation coefficient between two samples. Each small RNA profile was obtained from three independently grown cultures (independent experiments). (E) Principal component (PC) analysis of the ESC samples maintained under serum, 2i, and R2i culture conditions. Each small RNA-sequencing data was obtained from three independently grown cultures (independent experiments).

To obtain a comprehensive expression profile of miRNAs in ground-state ESCs, we used the RB18 and RB20 ESC lines maintained under feeder-free conditions in serum, 2i, or R2i cultures. RB18 and RB20 ESC lines were initially derived from C57BL/6 mice using the R2i + LIF protocol (Hassani et al., 2014b). Isolated R2i cells were then transferred to 2i or serum-containing medium and passaged at least 10 times to derive stable 2i and serum cell lines (Figure 1A). Pluripotency of established cell lines was confirmed by chimera formation and germline contribution as previously reported (Hassani et al., 2014b). Small RNA-sequencing data were obtained for both the RB18 and the EB20 ESC lines each time using pools of three independently grown cultures.

Analysis of the small RNA profiles of ESC samples revealed a total of 79,520,099 raw reads. After removal of adaptors and reads with <15–35> bases, the processed reads were aligned to mouse genome, which yielded a total of 35,287,315 mappable reads (Figure 1B). We found that on average 29.15% of the processed reads were matched to known mouse miRNAs (Figure 1C). Analysis of the length distribution revealed two peaks (Figure S1A). The minor peak represented Piwi-interacting RNAs (piRNAs with a size range of 26–31 nt) (Yang et al., 2013), whereas the major peak represented mature miRNAs (∼22 nt long) (Bartel, 2004).

A Pearson correlation coefficient heatmap (Figure 1D) revealed a remarkably high degree of correlation between miRNA profiles in serum, 2i, and the R2i cell lines, which was also supported by hierarchical co-clustering of the samples. The heatmap revealed that 2i and R2i cells showed greater similarity to each other compared with serum cells, which suggests that 2i and R2i cultures share a similar miRNA profile that might reflect the ESC ground state. Moreover, two-dimensional principal component analysis showed that 2i and R2i cells were located in close proximity to each other, but quite distinct from serum cells (Figure 1E), demonstrating that ground-state pluripotency features a unique signature of small RNA expression. Since both cell lines showed virtually identical results, we merged the data from both ESC lines for the subsequent analyses.

### Expression Patterns of miRNAs Associated with Pluripotency and Differentiation

Bioinformatics analysis identified a set of 20 miRNAs, which were abundantly expressed under all conditions in ESCs (Figure S1B). Members of the miR-290–295 cluster represented the most highly expressed miRNAs, as previously reported (Marson et al., 2008). Interestingly, expression of most members of the miR-290–295 cluster did not change significantly in different culture conditions (Figure S2A). Likewise, we observed that several other pluripotency-associated miRNAs (e.g., miR-182-5p and miR-183-5p) were expressed at similarly high levels under all conditions (Figure S1C), suggesting that the most abundant miRNAs in ESCs did not undergo major expression changes under different culture conditions.

In contrast, members of the miR-302–367 cluster were mostly upregulated in 2i/R2i cells compared with serum ESCs (Figure S2B). This finding is in disagreement with results reported by Parchem et al. (2015), who suggested that miR-302b is expressed at higher levels in serum compared with 2i cells. However, in the previous study 2i chemicals were added to serum-containing medium, whereas in the current study 2i (similar to R2i) cells were cultured serum free, which might explain the discrepancy. Analysis of the expression patterns of the pluripotency-associated miR-17 family (consisting of three clusters) revealed that most members of the miR-17–92 and miR-106b-25 clusters did not change their expression patterns in different ESC media (Figures S2C and S2D). In contrast, most members of the miR-106a∼363 cluster were upregulated in serum ESCs compared with 2i and R2i (Figure S2E).

We next asked whether miRNAs associated with differentiation were differentially expressed (Figure S2F). We found that the majority of differentiation-affiliated miRNAs were upregulated in serum cells compared with 2i and R2i, suggesting that serum + LIF failed to completely suppress differentiation-associated processes. Interestingly, we found that a small number of other differentiation-associated miRNAs including let-7g-5p were more abundant in ground state compared with serum cells, although their read numbers were very small (data not shown). This finding is consistent with a previous study showing increased expression of let-7 miRNAs in 2i compared with serum (Kumar et al., 2014). We hypothesize that some differentiation-associated miRNAs might promote some features of ground-state pluripotency and/or render ground-state cells “poised” for rapid differentiation once 2i/R2i + LIF components of the ESC medium are removed from culture.

**Figure 2:**
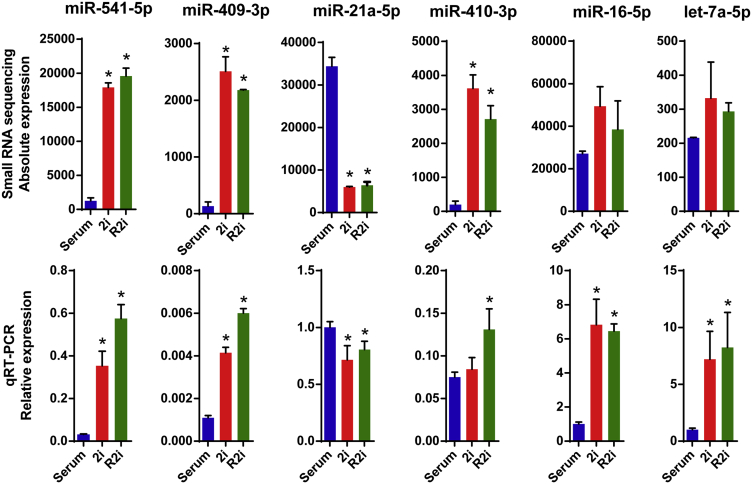
qRT-PCR Confirmation of the Small RNA-Sequencing Data A selected set of miRNAs with differential expression in serum, 2i, and R2i conditions were chosen for qRT-PCR confirmation. Data are shown as mean ± SD, n = 3. ^∗^p < 0.05. Each small RNA-sequencing datum was obtained from three independently grown cultures (independent experiments).

To validate the expression patterns of miRNAs obtained by small RNA sequencing, we performed TaqMan miRNA qRT-PCR assays of RNA isolated from serum, 2i, and R2i cells, and observed highly similar results compared with small RNA sequencing (Figure 2). However, we noted statistically significant differences in the expression of let-7a-5p and miR-16-5p between the two platforms. Nevertheless the differences were minor, indicating that the small RNA-sequencing data are suitable for further analysis.

### Identification of Differentially Expressed miRNAs

**Figure 3:**
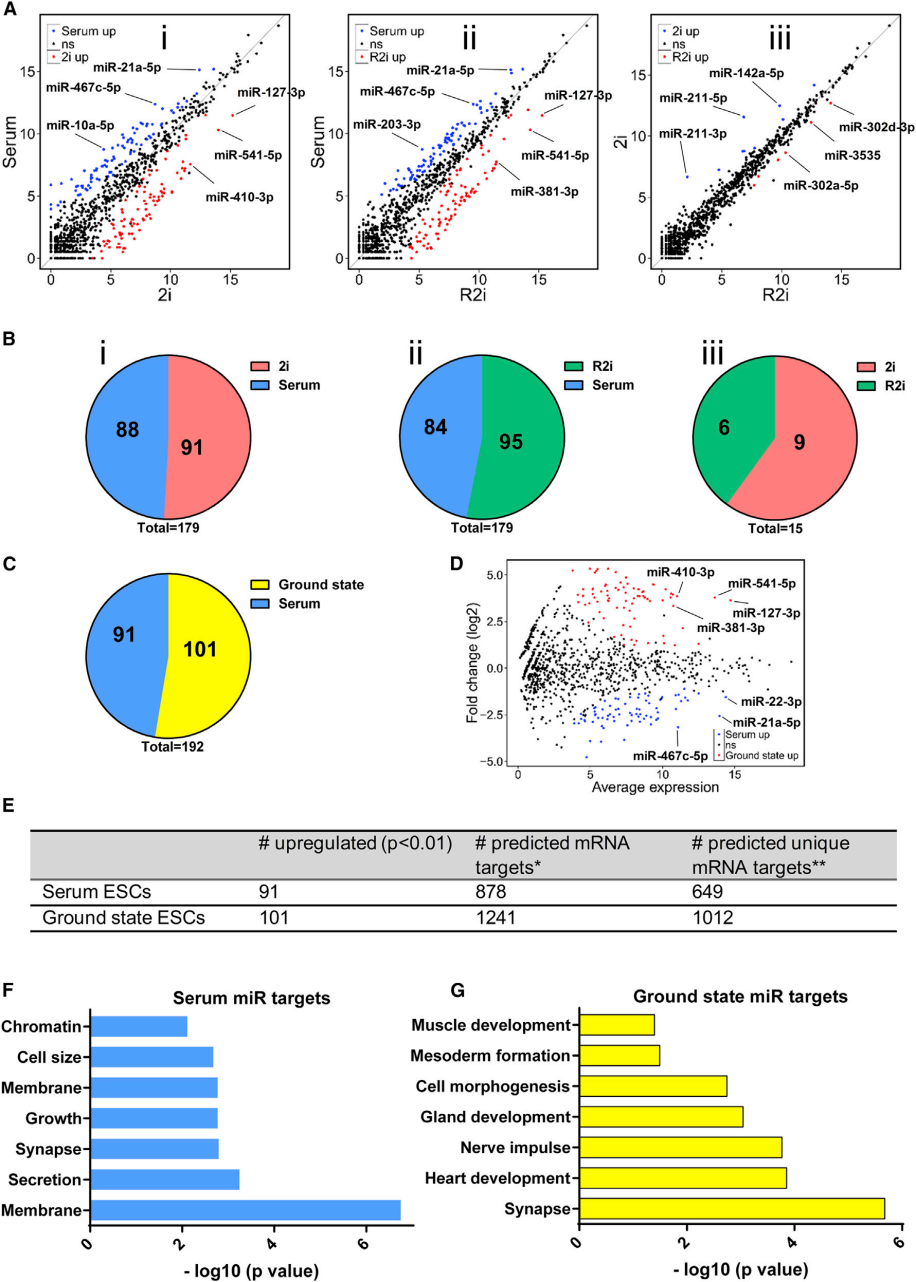
Differentially Expressed miRNAs (A) Scatterplots indicating the global expression pattern of miRNAs in (i) 2i versus serum, (ii) R2i versus serum, and (iii) 2i versus R2i. Each small RNA-sequencing datum was obtained from three independently grown cultures (independent experiments). (B) Pie charts indicating the number of differentially expressed miRNAs for (I) 2i versus serum, (ii) R2i versus serum, and (iii) 2i versus R2i. Each small RNA-sequencing datum was obtained from three independently grown cultures (independent experiments). (C) Pie chart showing the number of differentially expressed miRNAs for ground-state ESCs versus serum. Each small RNA-sequencing datum was obtained from three independently grown cultures (independent experiments). (D) Scatterplot indicating the global expression pattern of miRNAs in ground-state ESCs versus serum. Each small RNA-sequencing datum was obtained from three independently grown cultures (independent experiments). (E) miRNAs upregulated in serum and ground-state cells plus the number of transcripts predicted to be targeted by these miRNAs before and after the exclusion of commonly targeted mRNAs. (F and G) GO analysis of transcripts predicted to be targeted by ground-state (F) and serum-associated miRNAs (G).

Our small RNA-sequencing approach detected 1,233 miRNAs in each ESC state. Scatterplots indicated that miRNAs were significantly differentially expressed under different conditions (Figures 3Ai–iii). Some miRNAs such as miR-127-3p and miR-410-3p were upregulated in 2i versus serum, whereas miR-467c-5p and miR-10a-5p were upregulated in serum (Figure 3Ai). We observed that miR-21a-5p and miR-203-3p were expressed significantly higher in serum than in R2i, whereas miR-381-3p and miR-127-3p were expressed much higher in R2i than in serum (Figure 3Aii). Moreover, although the miRNome of 2i and R2i states was strikingly similar, some miRNAs such as miR-211-5p and miR-142a-5p were differentially expressed between 2i and R2i (Figure 3Aiii). These data show that ESCs' miRNA expression changes dynamically in response to changes in extrinsic signals.

A global assessment of miRNAs exhibiting >2-fold differences in expression revealed that 179 miRNAs were differentially expressed between serum and 2i cells (Figure 3Bi; Tables S1 and S2). Additionally, when comparing R2i with serum cells, we found that 95 miRNAs were upregulated in R2i and 84 miRNAs were upregulated in serum (Figure 3Bii; Tables S3 and S4). In addition, 2i cells expressed nine miRNAs more abundantly than R2i cells, whereas R2i cells expressed six miRNAs more abundantly than 2i cells (Figure 3Biii and Table S5).

Next, we sought to pinpoint miRNAs associated with serum or ground-state pluripotency. We defined ground-state-associated miRNAs as miRNAs that are significantly upregulated in both 2i and R2i. Our data identified 91 miRNAs associated with serum and 101 miRNAs associated with the ground state (Figure 3C and Table S6). Top serum-enriched miRNAs included miR-21a-5p and miR-467c-5p, while top miRNAs upregulated in ground state included miR-381-3p and miR-541-5p (Figure 3D). To identify the biological pathways that are potentially modulated by serum and ground-state miRNAs in ESCs, we performed miRNA target prediction using TargetScan for the miRNAs enriched in each ESC state (Figure 3E). A total of 878 transcripts were predicted to be targeted by miRNAs under serum conditions and 1,241 transcripts were potentially targeted by miRNAs specific to ground state. To identify only pathways that are “specifically” regulated by state-specific miRNAs, we excluded all transcripts co-targeted by both sets of miRNAs. This rationale reduced the number of predicted targets to 649 (serum) and 1,012 (ground state) (Table S7). DAVID analysis revealed that miRNAs associated with the serum condition did not modulate critical pathways associated with fate decision in ESCs (Figure 3F), while miRNAs associated with ground-state pluripotency were predicted to control key developmental processes predominantly related to differentiation (Figure 3G). Our gene ontology (GO) analysis using Enrichr also indicated that in contrast to serum-up miRNAs, which did not seem to modulate ESC fate decisions, ground-state-up miRNAs targeted several crucial differentiation-associated pathways including neurogenesis and organ morphogenesis (Figure S3). We reasoned that miRNAs upregulated in ground-state cells might contribute to the inhibition of differentiation, which is in line with the concept that ground-state pluripotency is achieved by repression of differentiation processes (Wray et al., 2010, Ying et al., 2008).

### Chromosomes Differentially Contribute to the Production of Mature miRNAs in ESCs

**Figure 4:**
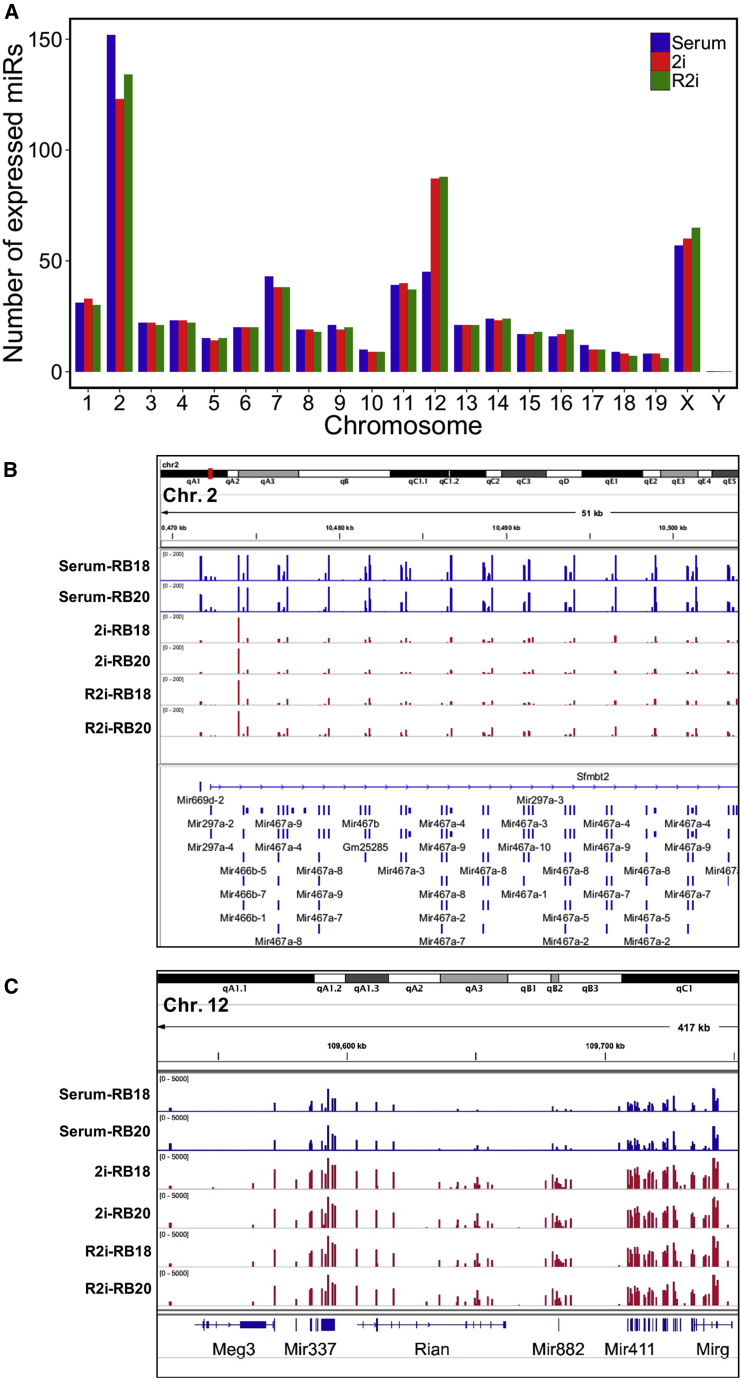
Contributions of Chromosomes to miRNA Generation in ESCs (A) Chromosome pattern of miRNA expression in serum, 2i, and R2i cultures. The y axis indicates the number of miRNA-coding sequences within chromosomes which are transcribed as mature miRNAs. Each small RNA-sequencing datum was obtained from three independently grown cultures (independent experiments). (B) Integrative Genomics Viewer (IGV) browser screenshot indicating miRNA read alignments in the 10^th^ intron of *Sfmbt2* locus (chromosome 2). Small RNA-sequencing data were obtained for two ESC lines (RB18 and EB20) each time using pools of three independently grown cultures (independent experiments). (C) IGV visualization of the read alignments of miRNAs embedded within the *Dlk1-Dio3* locus (chromosome 12). Small RNA-sequencing data were obtained for two ESC lines (RB18 and EB20) each time using pools of three independently grown cultures (independent experiments).

Next, we sought to profile the global chromosomal distribution of miRNAs detected in the different ESC samples. miRNAs were mapped to different chromosomes and the relative expression of mature miRNAs per chromosome was determined. miRNAs were encoded on and expressed in ESCs by all chromosomes other than the Y chromosome (Figures 4A and S4A). Four chromosomes (6, 7, 14, and X) were observed to transcribe miRNAs more actively than other chromosomes (Figure S4A) and, interestingly, these four chromosomes expressed the most abundant miRNAs in ESCs: chromosome 6 harbors miR-182, miR-183, and miR-148a species; chromosome 7 harbors the miR-290–295 cluster; chromosome 14 harbors the miR-17–92 cluster and miR-16; and chromosome X harbors the miR-106a∼363 cluster. These observations indicate that chromosomes 6, 7, 14, and X code for and express the most abundant miRNAs in ESCs (see Figures S1B and S1C).

Next, we determined how many miRNA genes are active per chromosome in different ESCs. As shown in Figure 4A, two chromosomes (2 and X) in serum ESCs and three chromosomes (2, 12, and X) in 2i/R2i ESCs activated the largest number of miRNA genes. Since chromosomes 2 and 12 showed a significant difference in the number of miRNA genes they activated under different cultures, we focused on these two chromosomes and found, interestingly, that most members of a repetitive miRNA cluster within the 10^th^ intron of the imprinted *Sfmbt2* gene located on chromosome 2 were expressed much higher in serum compared with 2i/R2i (Figure 4B). Moreover, we found that approximately all members of a large miRNA cluster embedded in an imprinted region overlapping with the developmentally important *Dlk1-Dio3* locus on chromosome 12 (12qF1) were expressed much higher in 2i/R2i than in serum (Figure 4C). Surprisingly, these *Dlk1-Dio3* locus-embedded miRNAs constituted the majority of miRNAs upregulated in ground-state cells (Figure S4B). In conclusion, we uncovered a ground-state-specific, imprinted genomic region coding for miRNAs that can serve as a signature for ground-state pluripotency and might be of functional importance.

### Ground-State-Associated miRNAs Promote ESC Self-Renewal

**Figure 5:**
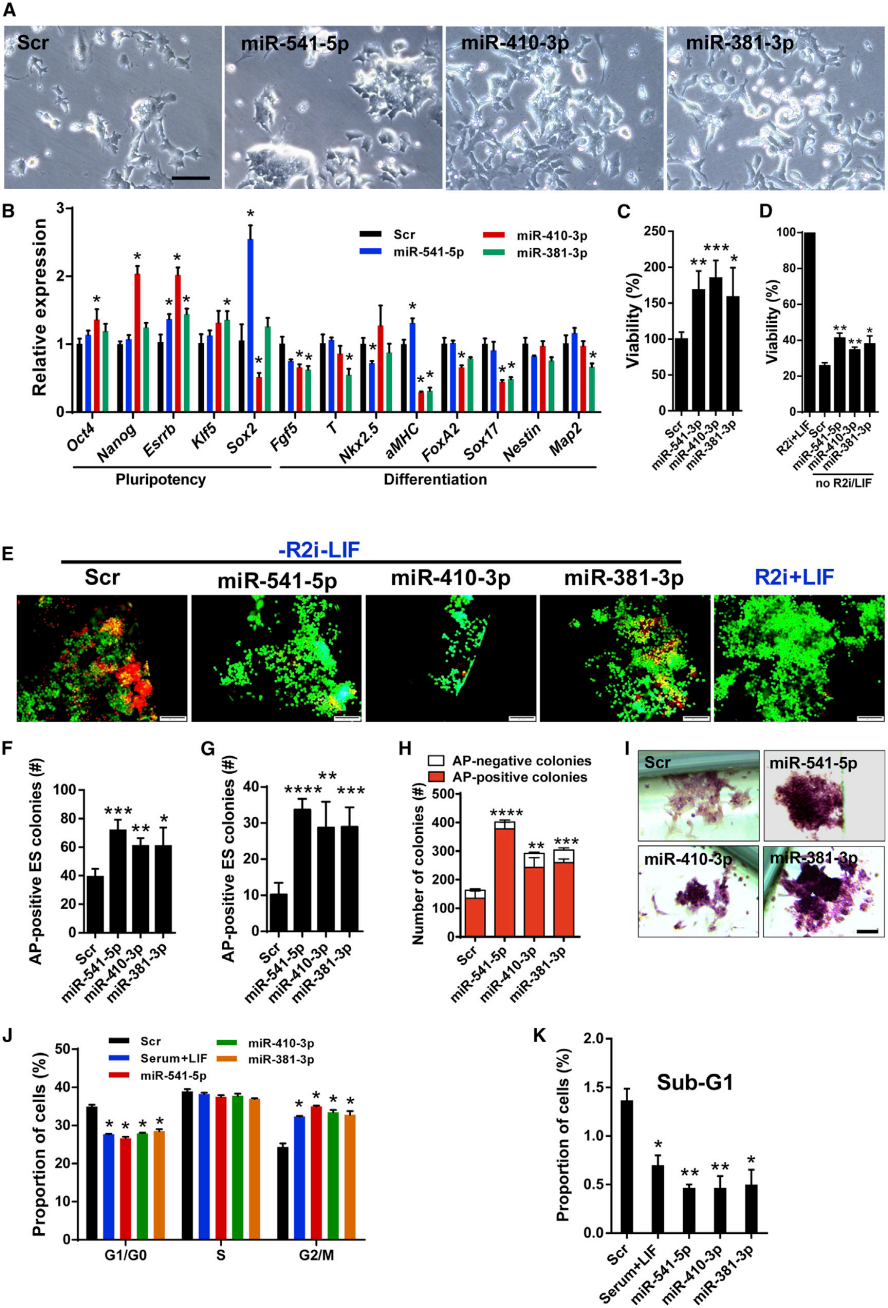
Effect of Ground-State-Associated miRNAs on ESC Self-Renewal (A) Phase-contrast image of serum ESCs treated with representative ground-state miRNAs. Scale bar, 100 μm. (B) qRT-PCR analysis of pluripotency- and differentiation-associated transcripts 3 days following serum ESC treatment with ground-state miRNAs. *Gapdh* was used as an internal normalization control. Data are shown as mean ± SD, n = 3. ^∗^p < 0.05. (C) MTS assay of ESCs 3 days after transfection of miRNAs. Data are shown as mean ± SD, n = 3. ^∗^p = 0.0311, ^∗∗^p = 0.0029, ^∗∗∗^p = 0.0007. (D) MTS assay of ESCs 3 days after transfection with miRNAs in the presence or absence of R2i + LIF. Data are shown as mean ± SD, n = 3. ^∗^p = 0.0111, ^∗∗^p < 0.0025. (E) Live/dead staining of ESCs 3 days after transfection with miRNAs in the presence or absence of R2i + LIF. Scale bars, 100 μm. (F) Number of AP-positive ESC colonies grown in the presence of LIF 5 days after transfection of miRNAs. Data are shown as mean ± SD, n = 4. ^∗^p = 0.0295, ^∗∗^p = 0.0015, ^∗∗∗^p = 0.0004. (G) Number of AP-positive ESC colonies grown in the absence of LIF 5 days after transfection of miRNAs. Data are shown as mean ± SD, n = 4. ^∗∗^p = 0.0032, ^∗∗∗^p = 0.001, ^∗∗∗∗^p < 0.0001. (H) ESC clonogenicity assay following serum ESC treatment with miRNAs. Cells were stained for AP activity 8 days after seeding. AP-positive and -negative colonies were counted under the microscope. Data are shown as mean ± SD, n = 3. ^∗∗^p = 0.0017, ^∗∗∗^p = 0.0002, ^∗∗∗∗^p < 0.0001. (I) AP staining of serum ESCs transfected with miRNAs on day 8. Scale bar, 50 μm. (J) Cell-cycle profiling of serum ESCs treated with miRNAs using flow cytometry 3 days after transfection. Scr (serum ESCs without LIF at the presence of a control scrambled oligonucleotide) is the negative control and serum + LIF is the positive control. miRNA mimics were added to LIF-withdrawn ESCs, which allowed us to score the effects of miRNA mimics relative to the positive and negative controls. Data are shown as mean ± SD, n = 3. ^∗^p < 0.05. (K) Ratio of cells in sub-G_1_ phase of the cell cycle 3 days following serum ESC treatment with miRNAs based on flow-cytometry analysis of the cell cycle. Data are shown as mean ± SD, n = 3. ^∗^p < 0.05, ^∗∗^p < 0.0065.

The association of chromosome 12-located miRNA gene expression with ground-state ESCs raised the intriguing question of whether these miRNAs directly affect acquisition or maintenance of the ground state. To evaluate this possibility, we selected three ground-state-associated miRNAs located within the imprinted *Dlk1-Dio3* locus (miR-541-5p, miR-410-3p, and miR-381-3p) based on the following: (1) their high abundance compared with other ground-state-specific miRNAs (see Figure 3C) and (2) their putative ability to target diverse differentiation pathways (Tables S8). After confirmation of efficient delivery of the candidate miRNAs into cultured mouse embryonic fibroblasts (Figure S4C), we treated undifferentiated serum-grown ESCs with individual miRNAs and analyzed morphology and expression of different stemness and differentiation genes 3 days after transfection. ESCs treated with candidate miRNAs exhibited typical ESC colony formation with more compact morphology compared with the Scr control (Figure 5A). qRT-PCR analysis revealed that miRNA-transfected ESCs expressed higher levels of stemness genes and lower levels of differentiation genes (Figure 5B). In addition, we found that miR-541-5p, miR-410-3p, and miR-381-3p promoted the viability of ESCs 3 days post treatment as measured by the MTS assay (Figure 5C). Moreover, transfection of miR-541-5p, miR-410-3p, and miR-381-3p into ground-state R2i ESCs cultured for 3 days in the absence of R2i/LIF (a condition that considerably reduces ESC viability) significantly enhanced viability (Figure 5D). Additional assessments of viability using the Live/Dead Viability/Cytotoxicity Kit validated these results (Figure 5E). Furthermore, we observed an increase of the number of alkaline phosphatase (AP)-positive ESC colonies 5 days post transfection in both the presence (Figure 5F) and absence of LIF (Figure 5G). Notably, overexpression of miRNAs also enhanced AP activity in ESCs cultured without LIF for 5 days (Figure S4D), further supporting our conclusion that ground-state-associated miRNAs promote pluripotency and partially “rescue” LIF-free ESCs from differentiation.

Since ground-state-specific miRNAs promoted ESC viability and 2i and R2i cells exhibit a much higher cloning efficiency than serum cells (Hassani et al., 2014b), we wanted to determine the colony-forming ability of ESCs transfected with miR-541-5p, miR-410-3p, and miR-381-3p. Five days after reseeding of day-3 transfected serum ESCs, we analyzed AP activity by counting AP-positive and -negative colonies and observed a significant increase of the number of AP-positive colonies by all three miRNAs but particularly by miR-541-5p (Figure 5H), as well as a general enhancement of AP staining intensity (Figure 5I). These results indicated that ground-state-associated miRNAs boosted AP activity and markedly promoted ESC clonogenicity.

Ground-state miRNAs might influence ESC viability and clonogenicity by modulating the ESC cycle. We focused specifically on the G_1_ phase, which is shortened in ESCs compared with differentiated cells and the extension of G_1_ phase upon differentiation (Becker et al., 2006, Becker et al., 2010, Calder et al., 2013, Coronado et al., 2013, Li et al., 2012). To induce ESC differentiation, we removed LIF from the cultures and concomitantly added miRNAs. Three days after transfection, differentiating ESCs were analyzed by flow cytometry. As expected, we observed prolonged G_0_/G_1_ phases in Scr-treated control cells after LIF withdrawal, indicative of the exit from pluripotency. In contrast, transfection with either miR-541-5p, miR-410-3p, or miR-381-3p shortened the prolonged G_1_ phase of differentiating ESCs, which reached values characteristic for LIF-grown ESCs (Figure 5J). Similarly, all three miRNAs reduced the sub-G_1_ phase under LIF-free conditions (Figure 5K), which implicates improved survival upon initiation of differentiation.

**Figure 6:**
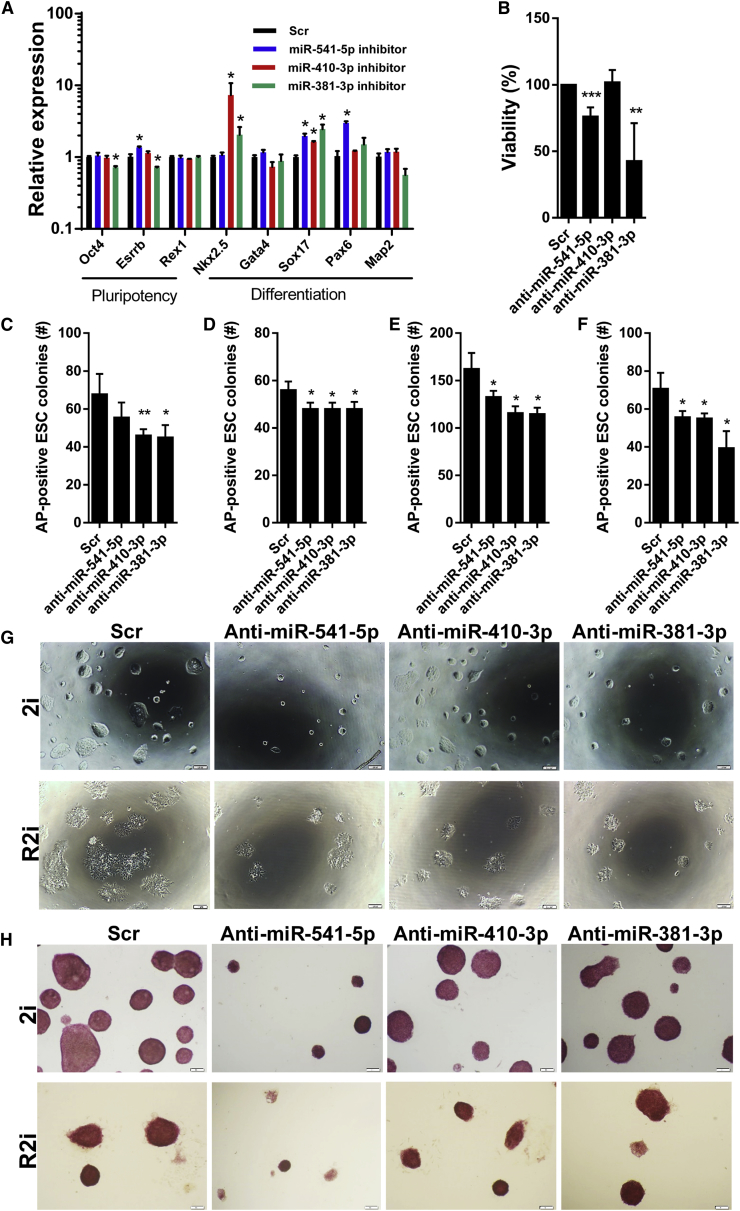
Effect of Ground-State-Associated miRNA Inhibitors on ESC Self-Renewal (A) qRT-PCR analysis of pluripotency- and differentiation-associated transcripts 3 days following 2i ESC treatment with the inhibitors of ground-state miRNAs. *Gapdh* was used as an internal normalization control. Data are shown as mean ± SD, n = 3. ^∗^p < 0.05. (B) MTS assay of 2i ESCs 3 days after transfection of miRNA inhibitors. Data are shown as mean ± SD, n = 3. ^∗∗^p = 0.0029, ^∗∗∗^p = 0.0007. (C) Number of AP-positive 2i ESC colonies 3 days after transfection of miRNA inhibitors. Data are shown as mean ± SD, n = 3. ^∗^p < 0.05, ^∗∗^p = 0.0015. (D) Number of AP-positive R2i ESC colonies 3 days after transfection of miRNA inhibitors. Data are shown as mean ± SD, n = 3. ^∗^p < 0.05. (E) 2i ESC clonogenicity assay 8 days following treatment with miRNA inhibitors. Data are shown as mean ± SD, n = 3. ^∗^p < 0.05. (F) R2i ESC clonogenicity assay 8 days following treatment with miRNA inhibitors. Data are shown as mean ± SD, n = 3. ^∗^p < 0.05. (G) Phase-contrast image of 2i and R2i ESC colonies 3 days after treatment with miRNA inhibitors. Scale bars, 100 μm. (H) AP staining of 2i and R2i ESC colonies 8 days after treatment with miRNA inhibitors. Scale bars, 100 μm.

To corroborate our results, we asked whether inhibition of identified miRNAs will disrupt the ESC ground state. Transfection of ground-state ESCs with miR-541-5p, miR-410-3p, and miR-381-3p antagomirs induced upregulation of differentiation-associated genes 3 days after treatment (Figure 6A). Inhibition of miR-541-5p and miR-381-3p also reduced cellular viability in MTS assays (Figure 6B). Moreover, inhibition of ground-state miRNAs not only reduced the number of AP-positive 2i/R2i ESC colonies 3 days after miRNA inhibitor delivery (Figures 6C and 6D) but also the clonogenicity of the cells 8 days post transfection (Figures 6E and 6F). Of note, we also observed that inhibition of miR-541-5p remarkably decreased the colony size in both 2i and R2i ESCs 3 days (Figure 6G) and 8 days post transfection (Figure 6H). Taken together, our data indicate that miR-541-5p, miR-410-3p, and miR-381-3p promote ESC self-renewal.

### Ground-State-Associated miRNAs Inhibit Multi-lineage Differentiation of ESCs

**Figure 7:**
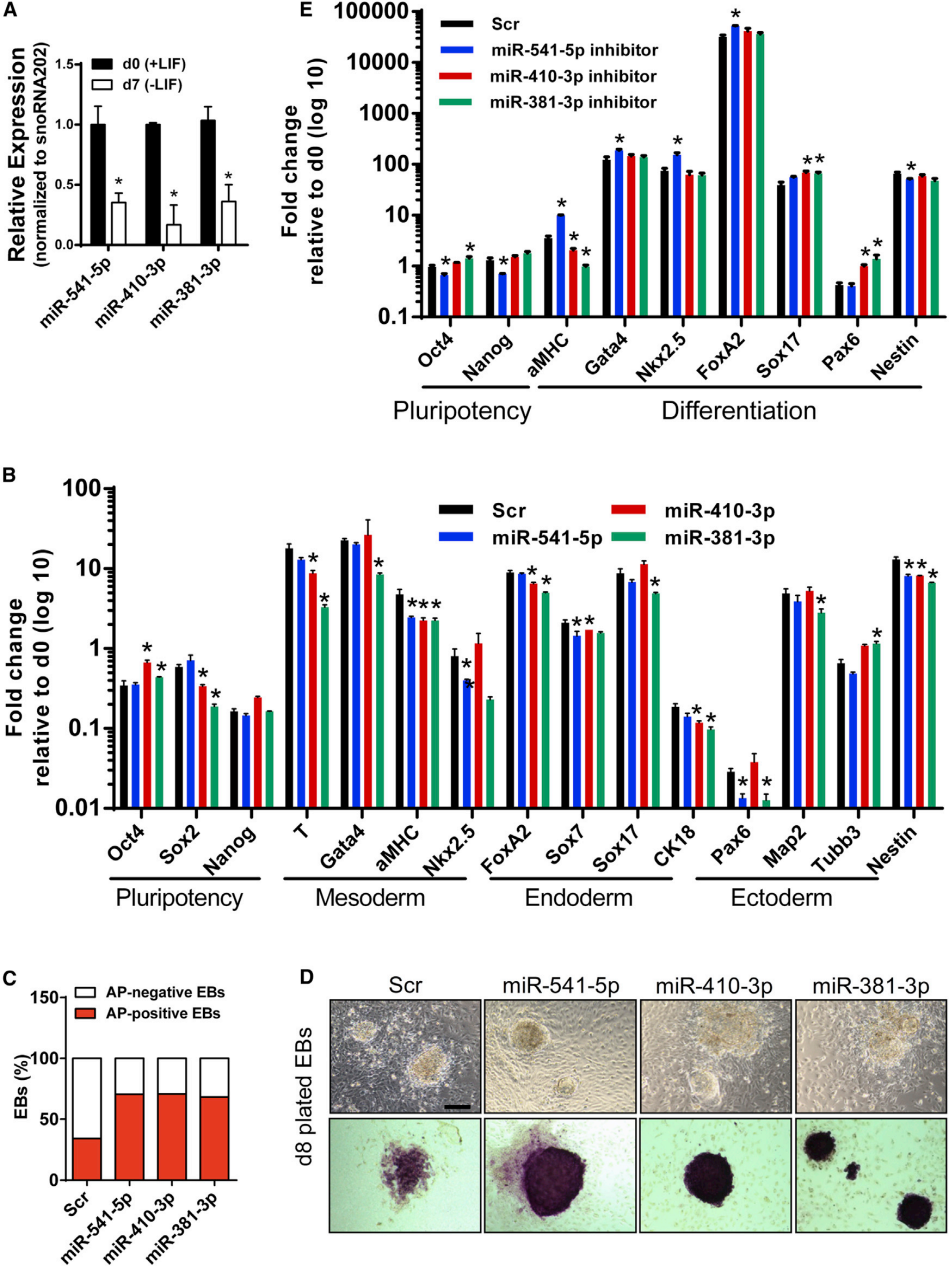
Effect of Ground-State-Associated miRNAs on ESC Differentiation (A) qRT-PCR analysis of miRNAs 7 days after induction of EB formation. snoRNA202 was used as an internal normalization control. Data are shown as mean ± SD, n = 3. ^∗^p < 0.0001. (B) qRT-PCR analysis of transcripts associated with pluripotency and differentiation 7 days following serum ESC treatment with miRNAs in the absence of LIF. *Gapdh* was used as an internal normalization control. Data are shown as mean ± SD, n = 3. ^∗^p < 0.05. (C) Percentages of AP-positive and -negative EBs 7 days after transfection of ESCs in the absence of LIF. (D) AP staining of EBs treated with miRNAs 7 days post differentiation. Scale bar, 200 μm. (E) qRT-PCR analysis of gene transcripts associated with pluripotency and differentiation 7 days following 2i ESC treatment with miRNA inhibitors in the absence of 2i/LIF. *Gapdh* was used as an internal normalization control. Data are shown as mean ± SD, n = 3. ^∗^p < 0.05.

We hypothesized that miR-541-5p, miR-410-3p, and miR-381-3p might contribute to the maintenance of ground-state ESCs by inhibiting differentiation, which would fit into the observed downregulation of these miRNAs 7 days after initiation of differentiation (Figure 7A). For analysis of the effects of miR-541-5p, miR-410-3p, and miR-381-3p on ESC differentiation, undifferentiated serum ESCs were seeded 1 day prior to miRNA transfection and ESCs were induced to form embryoid bodies (EBs) in LIF-free medium 1 day after transfection. EBs were harvested at day 7 and subjected to qRT-PCR expression analysis of key genes representing pluripotency and differentiation.

Interestingly, we found that all candidate miRNAs repressed the majority of genes characteristic for ESC differentiation into different germ layers (Figure 7B), suggesting that they block multi-lineage differentiation of ESCs. We also detected increased numbers of AP-positive EBs compared with the Scr control 7 days after differentiation (Figure 7C). Similar to our previous results with undifferentiated ESCs cultured in monolayers, we observed increased AP activity of miRNA-treated EBs (Figure 7D). Next, we examined whether inhibition of ground-state miRNAs stimulates multi-lineage differentiation of ground-state ESCs. To this end, we treated the cells with miRNA inhibitors 2 days prior to EB induction and then harvested the cells for qRT-PCR analysis 10 days post treatment. We observed a significant upregulation of most of the tested differentiation-associated genes (Figure 7E), which is consistent with the data obtained using miRNA mimics. Taken together, our results indicate that miR-541-5p, miR-410-3p, and miR-381-3p contribute to the maintenance of ESC pluripotency and self-renewal by blocking differentiation.

### DNA Methylation Status of the *Dlk1-Dio3* Locus

Since (1) most differentially expressed miRNA genes are located in imprinted regions of the genome and (2) serum cells own higher DNA methylation levels than 2i (Ficz et al., 2013, Habibi et al., 2013, Yamaji et al., 2013), we reasoned that differences in DNA methylation might contribute to differential miRNA gene expression from the *Dlk1-Dio3* locus in ESCs. To test this hypothesis, we reanalyzed previously published DNA methylome data of serum and 2i cells (Angermueller et al., 2016). The average DNA methylation ratio in serum and 2i ESCs at the *Dlk1-Dio3* locus was accounted for 73.2% and 37.6%, respectively (Figure S5A), indicating that the *Dlk1-Dio3* locus is hypomethylated in 2i compared with serum. To confirm this result, we subjected DNA from serum, 2i, and R2i ESCs to methylation-sensitive restriction enzyme digestion. The resulting genomic fragments flanking specific restriction sites within the *Dlk1-Dio3* locus were analyzed by qPCR using specific primers. We found that ground-state ESCs compared with serum cells exhibited a significantly lower DNA methylation level at the three differentially methylated regions present in the *Dlk1-Dio3* locus (Figure S5B). We hypothesize that the higher DNA methylation levels at the *Dlk1-Dio3* locus in serum cells explain the significantly lower expression of miRNAs embedded in this imprinted region.

### Putative Target Genes of miR-541-5p, miR-410-3p, and miR-381-3p

We next wanted to determine potential target genes of the candidate miRNAs. To achieve this goal, we intersected putative miRNA targets predicted by TargetScan (Table S7) with proteins that were downregulated in 2i and R2i ESCs relative to serum cells (Taleahmad et al., 2015; H.B., unpublished data). This approach narrowed the putative target list down to 10, 4, and 5 genes for miR-541-5p, miR-410-3p, and miR-381-3p, respectively (Figure S6A). Using qRT-PCR, we confirmed that miR-541-5p overexpression reduced prolyl 4-hydroxylase (*P4ha1*) (Figure S6B), miR-410-3p overexpression reduced serine-arginine rich splicing factor 11 (*Srsf11*) and poly(C)-binding protein 2 (*Pcbp2*) (Figure S6C), and miR-381-3p overexpression downregulated microtubule-associated protein 4 (*Map4*) (Figure S6D). *P4ha1* codes for a key enzyme implicated in the synthesis of collagen, which has been shown to function as a barrier in iPSC generation (Jiao et al., 2013). The extracellular matrix (ECM) is of critical importance to ESC maintenance, and the expression of collagens and other ECM components have been reported to be increased upon exit from ground-state pluripotency (Veluscek et al., 2016). *Srsf11* is a nuclear protein involved in pre-mRNA splicing (Shepard and Hertel, 2009, Zhang and Wu, 1996). *Pcbp2* encodes an RNA binding protein that regulates pre-mRNA splicing in the nucleus and mRNA stabilization in the cytoplasm (Ji et al., 2007, Ji et al., 2011, Makeyev and Liebhaber, 2002) and is implicated in the regulation of collagen synthesis (Stefanovic et al., 1997). *Map4* is a non-neuronal microtubule-interacting protein promoting tubulin polymerization (Nguyen et al., 1999). Map4 phosphorylation modulates microtubule assembly and dynamics, and cell cycling (Chang et al., 2002, Illenberger et al., 1996, Kitazawa et al., 2000). Although further investigation is required to uncover the exact function of these genes in serum and ground-state pluripotency, we can speculate that the *Dlk1-Dio3* locus-embedded miRNAs contribute to the maintenance of ground-state pluripotency by regulating ECM, cytoskeletal dynamics, RNA processing, and cell cycling.

## Discussion

Small RNA sequencing has been used to profile miRNAs in different plant and animal species, diverse cell types, and human tissue fluids (Bellingham et al., 2012, Chiang et al., 2010, Glazov et al., 2008, Landgraf et al., 2007, Sunkar et al., 2005, Vojtech et al., 2014), but similar datasets for ground-state ESCs have been missing so far. In the present study, we used small RNA sequencing to obtain expression profiles of miRNAs in ground-state (2i and R2i) ESCs versus serum ESCs. Several miRNAs were differentially expressed between ground-state ESCs and serum ESCs. Most miRNAs upregulated in both 2i and R2i ESCs are located within the *Dlk1-Dio3* locus. Interestingly, activity of the *Dlk1-Dio3* locus seems crucial to maintain pluripotency of iPSCs, and its reduced expression is associated with incomplete iPSC reprogramming and poor contribution to mouse chimeras (Liu et al., 2010, Stadtfeld et al., 2010). We assume that ESCs undergo a gradual decline of *Dlk1-Dio3* locus activity during the transition from ground to serum state and differentiation.

Interestingly, the majority of miRNAs that are preferentially expressed in serum compared with 2i and R2i ESCs are located in the imprinted locus *Sfmbt2*. Our study, therefore, identifies large sets of differentially expressed miRNAs at imprinted loci, which can serve as specific markers of ground-state pluripotency versus serum state. The lower DNA methylation level observed at the *Dlk1-Dio3* locus in ground-state cells might lead to a more accessible chromatin for pluripotency factors to bind and activate the miRNAs embedded in the locus. Of note, the imprinted *Sfmbt2* locus, which harbors a large miRNA cluster, does not seem to carry a classic DMR (Wang et al., 2011), suggesting that different mechanisms regulate the expression of miRNAs embedded in the *Sfmbt2* locus.

Results from a previous study indicated that miRNAs from the *Sfmbt2* locus are expressed at higher levels in serum ESCs compared with epiblast stem cells (EpiSCs) (Jouneau et al., 2012). Since we found higher levels of *Sfmbt2*-embedded miRNAs in serum ESCs versus 2i/R2i, it is tempting to speculate that expression of miRNAs embedded within *Sfmbt2* locus presents a key miRNA signature of naive pluripotency allowing distinction of different mouse pluripotent states.

We demonstrated using miRNA gain- and loss-of-function analyses that ground-state miRNAs within the *Dlk1-Dio3* locus contribute to the maintenance of ground-state pluripotency by enhancing viability, clonogenicity, AP activity, and ESC cycling as well as inhibiting differentiation. Our analysis revealed that the putative targets of miR-541-5p, miR-410-3p, and miR-381-3p are involved in cytoskeletal organization, ECM dynamics, control of RNA processing/decay, and cell cycling. These processes have previously been found to be important for pluripotency in general and ground-state pluripotency in particular (Chowdhury et al., 2010, Dalton, 2015, Ji and Tian, 2009, Kalkan et al., 2017, Salomonis et al., 2010, Taleahmad et al., 2015, Veluscek et al., 2016). Our findings provide a useful perspective to temporarily lock ESCs in the ground state either by exogenous introduction of cell-permeable miRNAs or by the identification of small molecules that increase ground-state miRNA expression.

## Experimental Procedures

Mouse ESC lines were cultivated on gelatinized tissue-culture plates and dishes (Sigma-Aldrich) and passaged every other day. Serum ESCs were cultured in the presence of 15% ES-qualified fetal bovine serum (HyClone), and 2i/R2i cells were cultured in serum-free N2B27 medium.

Additional experimental procedures are detailed in Supplemental Experimental Procedures.

## Author Contributions

H.B. and S. Moradi conceived and designed the study. S. Moradi, H.B., and T.B. designed experiments and analyzed and interpreted the data. S. Moradi performed most of the experiments and wrote the manuscript. H.B., T.B., and S.A. provided financial and administrative support, discussed the results, and approved the manuscript. S. Mollamohammadi and A.S. contributed to cell-culture and cell-cycle analysis. A.S.-Z. performed bioinformatics analysis. A.A. contributed to qRT-PCR and DNA methylation analysis. S.A., G.H.S., and S.G. contributed to data analysis and interpretation. All authors reviewed and confirmed the manuscript before submission.

## Accession Numbers

The processed data of small RNA sequencing is deposited in the NCBI Gene Expression Omnibus (GEO: GSE87174).

## Supplemental Information

Document S1. Supplemental Experimental Procedures, Figures S1–S6, and Tables S1–S6 and S8

Table S7. List of Predicted mRNA Targets Used for Gene Ontology and Pathway Analysis, Related to Figures 3 and 5

Document S2. Article plus Supplemental Information
