# Supplementary material for: Small RNA Sequencing Reveals Dlk1-Dio3 Locus-Embedded MicroRNAs as Major Drivers of Ground-State Pluripotency
Source: Stem Cell Reports. 2017 Nov 9;9(6):2081–96. doi: 10.1016/j.stemcr.2017.10.009 (PMC5785679; doi:10.1016/j.stemcr.2017.10.009)
Supplement: Document S1. Supplemental Experimental Procedures, Figures S1–S6, and Tables S1–S6 and S8 [file mmc1.pdf]

Stem Cell Reports, Volume *9*

Supplemental Information

Small RNA Sequencing Reveals *Dlk1-Dio3* Locus-Embedded MicroRNAs as Major Drivers of Ground-State Pluripotency

Sharif Moradi, Ali Sharifi-Zarchi, Amirhossein Ahmadi, Sepideh Mollamohammadi, Alexander Stubenvoll, Stefan Günther, Ghasem Hosseini Salekdeh, Sassan Asgari, Thomas Braun, and Hossein Baharvand

Supplemental Information

Small RNA Sequencing Reveals *Dlk1-Dio3* Locus-Embedded MicroRNAs as Major Drivers of Ground State Pluripotency

Sharif Moradi, Ali Sharifi-Zarchi, Amirhossein Ahmadi, Sepideh Mollamohammadi, Alexander Stubenvoll, Stefan Günther, Ghasem Hosseini Salekdeh, Sassan Asgari, Thomas Braun, Hossein Baharvand

INVENTORY OF SUPPLEMENTARY INFORMATION

I. Supplementary Data

Figure S1, related to Figures 1 and 3

Figure S2, related to Figure 3

Figure S3, related to Figure 3

Figure S4, related to Figures 4 and 5

Figure S5, related to Figure 4

Figure S6, related to Figures 5, 6, and 7

Table S1, related to Figure 3

Table S2, related to Figure 3

Table S3, related to Figure 3

Table S4, related to Figure 3

Table S5, related to Figure 3

Table S6, related to Figure 3

Table S7, related to Figures 3 and 5

Table S8, related to Figure 5

II. Supplemental Experimental Procedure

III. Supplemental References

I. Supplemental Data

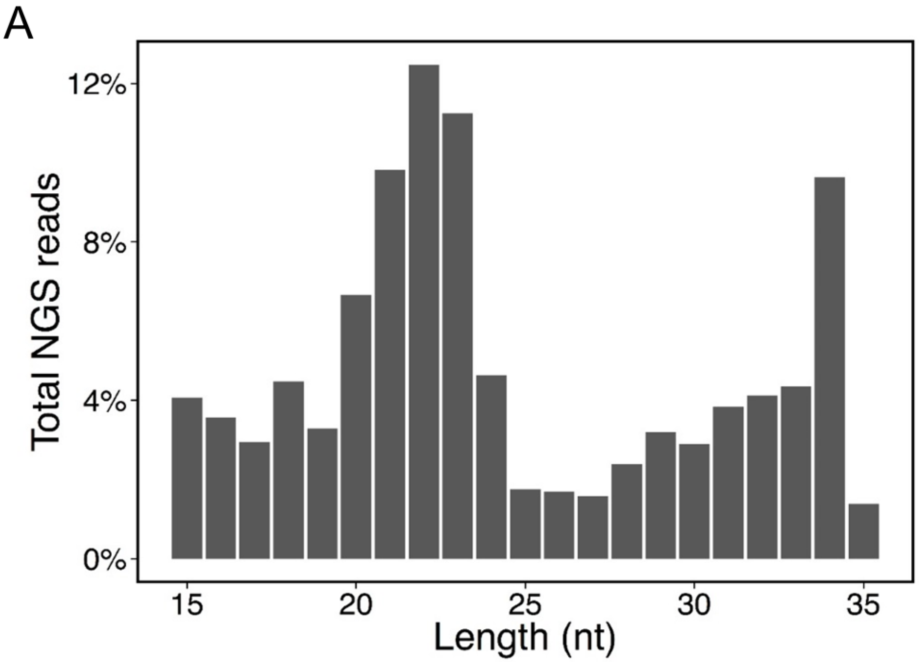

B Top 20: with miR-290 cluster

Serum	2i	R2i
mmu-miR-295-3p	mmu-miR-295-3p	mmu-miR-295-3p
mmu-miR-92a-3p	mmu-miR-182-5p	mmu-miR-182-5p
mmu-miR-182-5p	mmu-miR-183-5p	mmu-miR-92a-3p
mmu-miR-183-5p	mmu-miR-92a-3p	mmu-miR-183-5p
mmu-miR-292a-5p	mmu-miR-16-5p	mmu-miR-16-5p
mmu-miR-292a-3p	mmu-miR-26a-5p	mmu-miR-292a-3p
mmu-miR-291a-5p	mmu-miR-291a-5p	mmu-miR-292a-5p
mmu-miR-294-3p	mmu-miR-292a-3p	mmu-miR-291a-5p
mmu-miR-16-5p	mmu-miR-293-3p	mmu-miR-26a-5p
mmu-miR-26a-5p	mmu-miR-27b-3p	mmu-miR-293-3p
mmu-miR-293-3p	mmu-miR-292a-5p	mmu-miR-294-3p
mmu-miR-30e-5p	mmu-miR-294-3p	mmu-miR-30e-5p
mmu-miR-191-5p	mmu-miR-93-5p	mmu-miR-191-5p
mmu-miR-22-3p	mmu-miR-191-5p	mmu-miR-27b-3p
mmu-miR-21a-5p	mmu-miR-127-3p	mmu-miR-127-3p
mmu-miR-27b-3p	mmu-miR-30e-5p	mmu-miR-93-5p
mmu-miR-93-5p	mmu-miR-291a-3p	mmu-miR-291a-3p
mmu-miR-181c-5p	mmu-miR-148a-3p	mmu-miR-148a-3p
mmu-miR-291a-3p	mmu-miR-186-5p	mmu-miR-293-5p
mmu-miR-293-5p	mmu-miR-293-5p	mmu-miR-25-3p

C Top 20: without miR-290 cluster

Serum	2i	R2i
mmu-miR-92a-3p	mmu-miR-182-5p	mmu-miR-182-5p
mmu-miR-182-5p	mmu-miR-183-5p	mmu-miR-92a-3p
mmu-miR-183-5p	mmu-miR-92a-3p	mmu-miR-183-5p
mmu-miR-16-5p	mmu-miR-16-5p	mmu-miR-16-5p
mmu-miR-26a-5p	mmu-miR-26a-5p	mmu-miR-26a-5p
mmu-miR-30e-5p	mmu-miR-27b-3p	mmu-miR-30e-5p
mmu-miR-191-5p	mmu-miR-93-5p	mmu-miR-191-5p
mmu-miR-22-3p	mmu-miR-191-5p	mmu-miR-27b-3p
mmu-miR-21a-5p	mmu-miR-127-3p	mmu-miR-127-3p
mmu-miR-27b-3p	mmu-miR-30e-5p	mmu-miR-93-5p
mmu-miR-93-5p	mmu-miR-148a-3p	mmu-miR-148a-3p
mmu-miR-181c-5p	mmu-miR-186-5p	mmu-miR-25-3p
mmu-miR-148a-3p	mmu-miR-181c-5p	mmu-miR-541-5p
mmu-miR-25-3p	mmu-miR-25-3p	mmu-miR-302d-3p
mmu-miR-5099	mmu-miR-541-5p	mmu-miR-186-5p
mmu-miR-186-5p	mmu-miR-30d-5p	mmu-miR-130a-3p
mmu-miR-130a-3p	mmu-miR-22-3p	mmu-miR-5099
mmu-miR-7a-5p	mmu-miR-298-5p	mmu-miR-22-3p
mmu-miR-30a-5p	mmu-miR-130a-3p	mmu-miR-30d-5p
mmu-miR-26b-5p	mmu-miR-30c-5p	mmu-miR-30a-5p

Figure S1: Assessment of miRNA expression in serum, 2i, and R2i cultures, related to Figures 1 and 3

(A) Size distribution of small RNA sequences identified by small RNA sequencing

(B) Top 20 abundantly expressed miRNAs in three ESC culture conditions.

(C) Top 20 abundantly expressed miRNAs in three ESC culture conditions excluding the miR-290~295 cluster.

Small RNA sequencing data were obtained for two ESC lines (RB18 and EB20) each time using pools of three independently grown cultures.

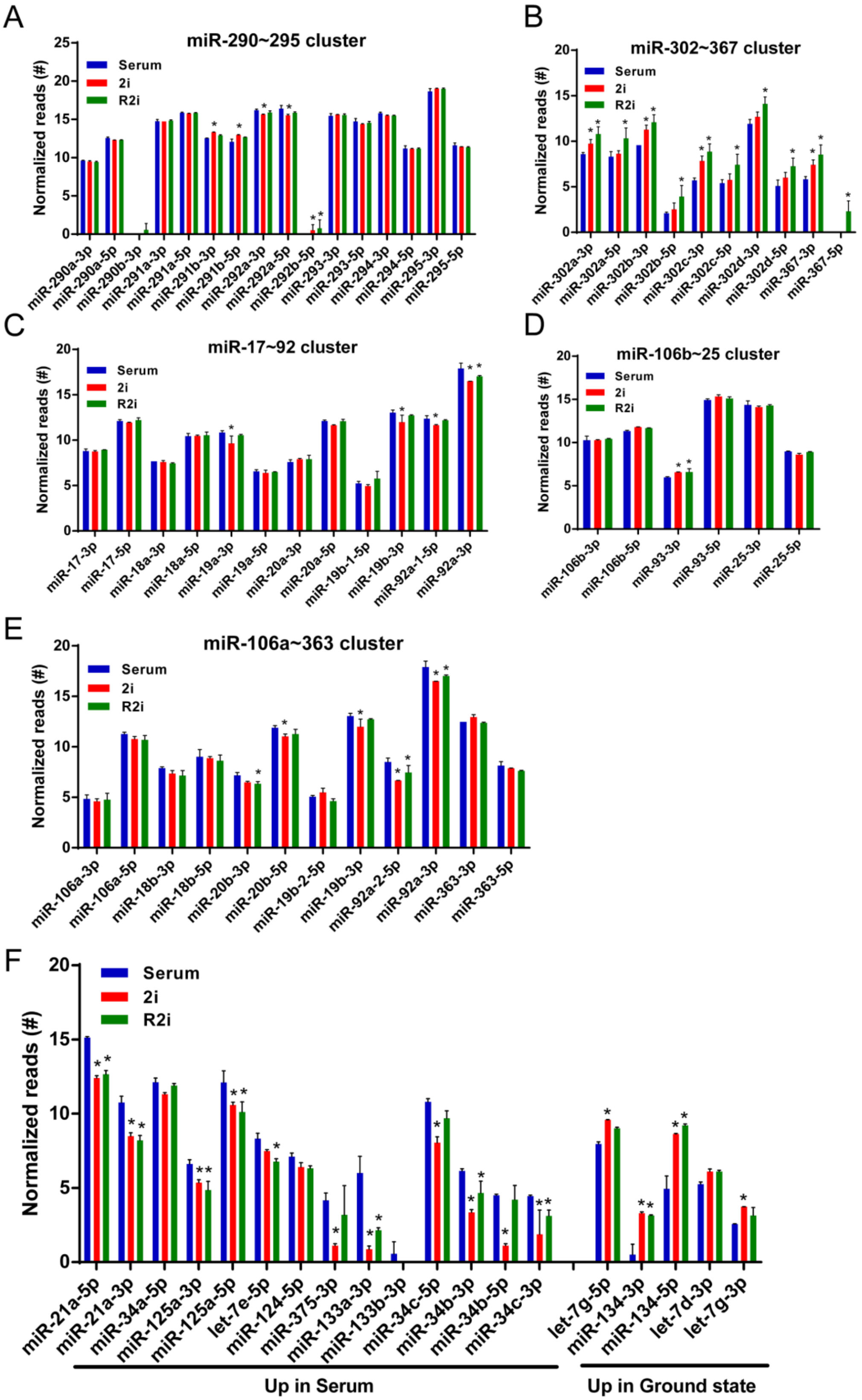
A
miR-290~295 cluster
Serum
2i
R2i
Normalized reads (#)
miR-290a-3p
miR-290a-5p
miR-290b-3p
miR-291a-3p
miR-291a-5p
miR-291b-3p
miR-291b-5p
miR-292a-3p
miR-292a-5p
miR-292b-5p
miR-293-3p
miR-293-5p
miR-294-3p
miR-294-5p
miR-295-3p
miR-295-5p
B
miR-302~367 cluster
Serum
2i
R2i
Normalized reads (#)
miR-302a-3p
miR-302a-5p
miR-302b-3p
miR-302b-5p
miR-302c-3p
miR-302c-5p
miR-302d-3p
miR-302d-5p
miR-367-3p
miR-367-5p
C
miR-17~92 cluster
Serum
2i
R2i
Normalized reads (#)
miR-17-3p
miR-17-5p
miR-18a-3p
miR-18a-5p
miR-19a-3p
miR-19a-5p
miR-20a-3p
miR-20a-5p
miR-19b-1-5p
miR-19b-3p
miR-92a-1-5p
miR-92a-3p
D
miR-106b~25 cluster
Serum
2i
R2i
Normalized reads (#)
miR-106b-3p
miR-106b-5p
miR-93-3p
miR-93-5p
miR-25-3p
miR-25-5p
E
miR-106a~363 cluster
Serum
2i
R2i
Normalized reads (#)
miR-106a-3p
miR-106a-5p
miR-18b-3p
miR-18b-5p
miR-20b-3p
miR-20b-5p
miR-19b-2-5p
miR-19b-3p
miR-92a-2-5p
miR-92a-3p
miR-363-3p
miR-363-5p
F
Serum
2i
R2i
Normalized reads (#)
miR-21a-5p
miR-21a-3p
miR-34a-5p
miR-125a-3p
miR-125a-5p
let-7e-5p
miR-124-5p
miR-375-3p
miR-133a-3p
miR-133b-3p
miR-34c-5p
miR-34b-3p
miR-34b-5p
miR-34c-3p
let-7g-5p
miR-134-3p
miR-134-5p
let-7d-3p
let-7g-3p
Up in Serum
Up in Ground state

Figure S2: Expression status of miRNAs associated with pluripotency and differentiation, related to Figure 3

(A) Bar plot depicting the expression pattern of members of the miR-290~295 cluster.

(B) Bar plot depicting the expression pattern of the miR-302~367 cluster.

(C-E) Bar plots depicting the expression patterns of the miR-17~92 cluster (E), miR-106b~25 cluster (F), and miR-106a-363 cluster (G).

(F) Bar plot depicting the expression pattern of miRNAs associated with differentiation.

We used two different ESC lines (RB18 and RB20). Each cell line was a pool of three independently grown cultures. Data are shown as mean ± SD. *$p<0.05$.

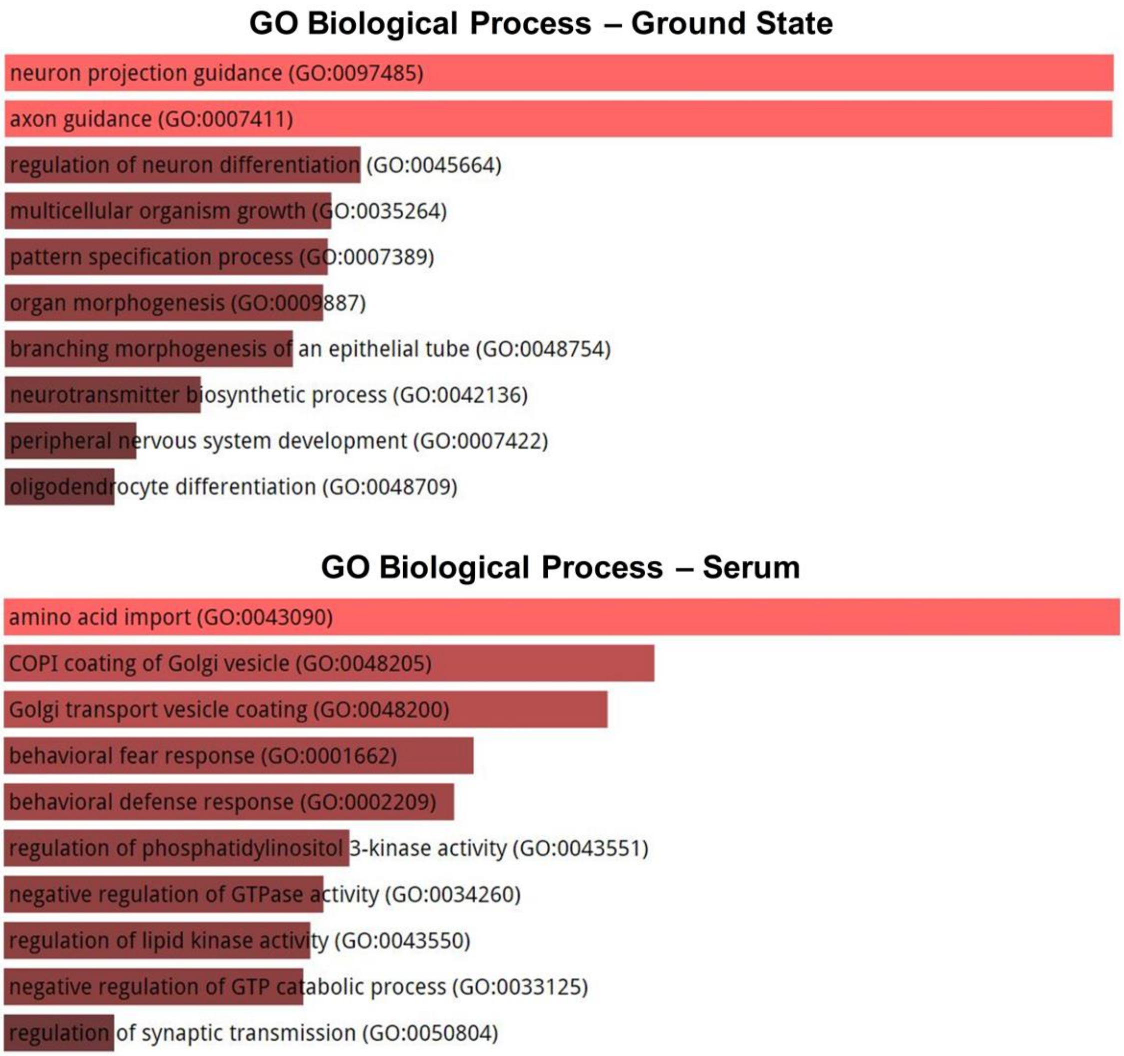

Figure S3: GO analysis of predicted targets of miRNAs associated with serum or ground state using Enrichr, related to Figure 3

A

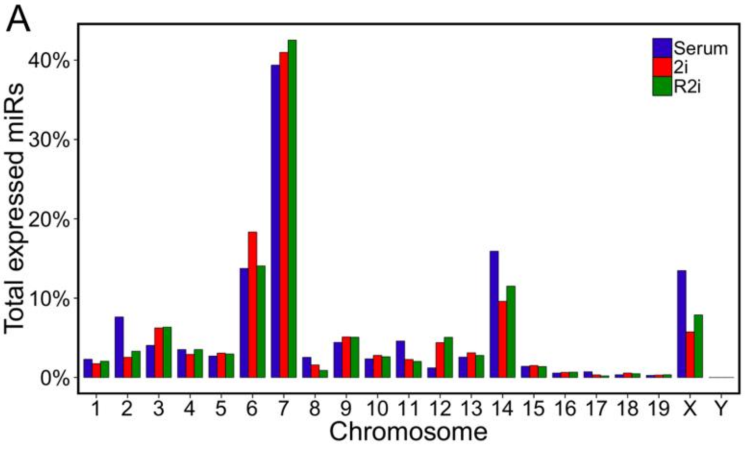

B

Ground state up miRs			
mmu-let-7g-5p	mmu-miR-301a-3p	mmu-miR-376b-5p	mmu-miR-485-5p
mmu-miR-1193-3p	mmu-miR-301a-5p	mmu-miR-376c-3p	mmu-miR-487b-3p
mmu-miR-1197-3p	mmu-miR-301b-3p	mmu-miR-377-3p	mmu-miR-494-3p
mmu-miR-1247-3p	mmu-miR-301b-5p	mmu-miR-379-3p	mmu-miR-495-3p
mmu-miR-127-3p	mmu-miR-302a-3p	mmu-miR-379-5p	mmu-miR-496a-3p
mmu-miR-127-5p	mmu-miR-302b-3p	mmu-miR-380-3p	mmu-miR-499-5p
mmu-miR-134-5p	mmu-miR-302c-3p	mmu-miR-381-3p	mmu-miR-505-3p
mmu-miR-136-3p	mmu-miR-3072-3p	mmu-miR-382-3p	mmu-miR-539-5p
mmu-miR-136-5p	mmu-miR-323-3p	mmu-miR-382-5p	mmu-miR-540-3p
mmu-miR-147-3p	mmu-miR-324-3p	mmu-miR-409-3p	mmu-miR-540-5p
mmu-miR-147-5p	mmu-miR-324-5p	mmu-miR-409-5p	mmu-miR-541-3p
mmu-miR-154-3p	mmu-miR-329-3p	mmu-miR-410-3p	mmu-miR-541-5p
mmu-miR-154-5p	mmu-miR-329-5p	mmu-miR-411-3p	mmu-miR-543-3p
mmu-miR-15a-3p	mmu-miR-337-3p	mmu-miR-411-5p	mmu-miR-665-3p
mmu-miR-15a-5p	mmu-miR-337-5p	mmu-miR-412-3p	mmu-miR-666-3p
mmu-miR-16-1-3p	mmu-miR-341-3p	mmu-miR-412-5p	mmu-miR-666-5p
mmu-miR-184-3p	mmu-miR-341-5p	mmu-miR-431-3p	mmu-miR-667-3p
mmu-miR-187-3p	mmu-miR-367-3p	mmu-miR-431-5p	mmu-miR-668-3p
mmu-miR-1981-5p	mmu-miR-369-3p	mmu-miR-433-3p	mmu-miR-673-3p
mmu-miR-199a-3p	mmu-miR-369-5p	mmu-miR-433-5p	mmu-miR-673-5p
mmu-miR-218-5p	mmu-miR-370-3p	mmu-miR-434-3p	mmu-miR-679-5p
mmu-miR-299a-3p	mmu-miR-370-5p	mmu-miR-434-5p	mmu-miR-743b-3p
mmu-miR-299a-5p	mmu-miR-376a-3p	mmu-miR-451a	mmu-miR-758-3p
mmu-miR-300-3p	mmu-miR-376a-5p	mmu-miR-470-5p	mmu-miR-871-3p
mmu-miR-300-5p	mmu-miR-376b-3p	mmu-miR-485-3p	mmu-miR-874-3p
mmu-miR-98-5p			
72 out of 101 ground state up miRs are embedded in Dlk-1-Dio3 locus			

C

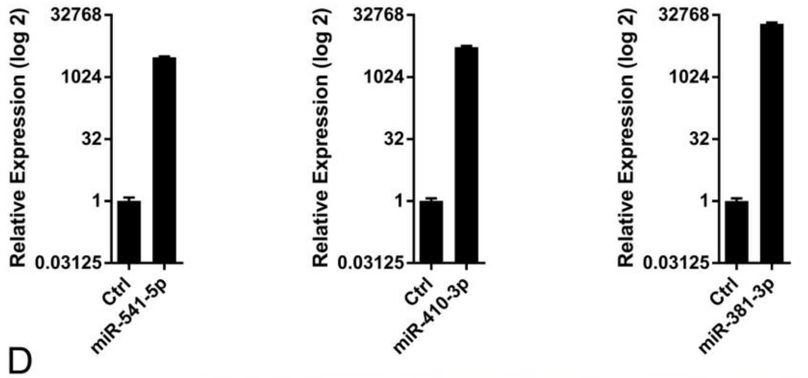

D

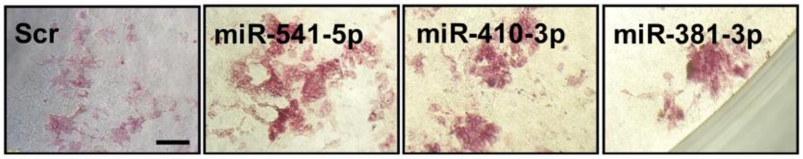

Figure S4: Expression of miRNA genes relative to chromosomal location and functional analysis of ground state-associated miRNAs, related to Figures 4 and 5

(A) Expression of miRNA genes relative to chromosomal location in (i) serum, (ii) 2i, and (iii) R2i cultures. Y-axis indicates the total read numbers of miRNAs expressed from different chromosomes. Small RNA sequencing data were obtained for two ESC lines (RB18 and EB20) each time using pools of three independently grown cultures.

(B) The majority of ground state up miRNAs are embedded in the *Dlk1-Dio3* locus. Ground state up miRNAs that are embedded in *Dlk1-Dio3* locus are shown in red font.

(C) qRT-PCR analysis indicating expression levels of miRNAs delivered into MEFs 1 day after transfection. snoRNA202 was used as an internal normalization control. Data are shown as mean ± SD, n=3.

(D) AP staining of LIF-deprived ESCs treated with miRNA mimics 5 days after transfection. Scale bar: 50 μm.

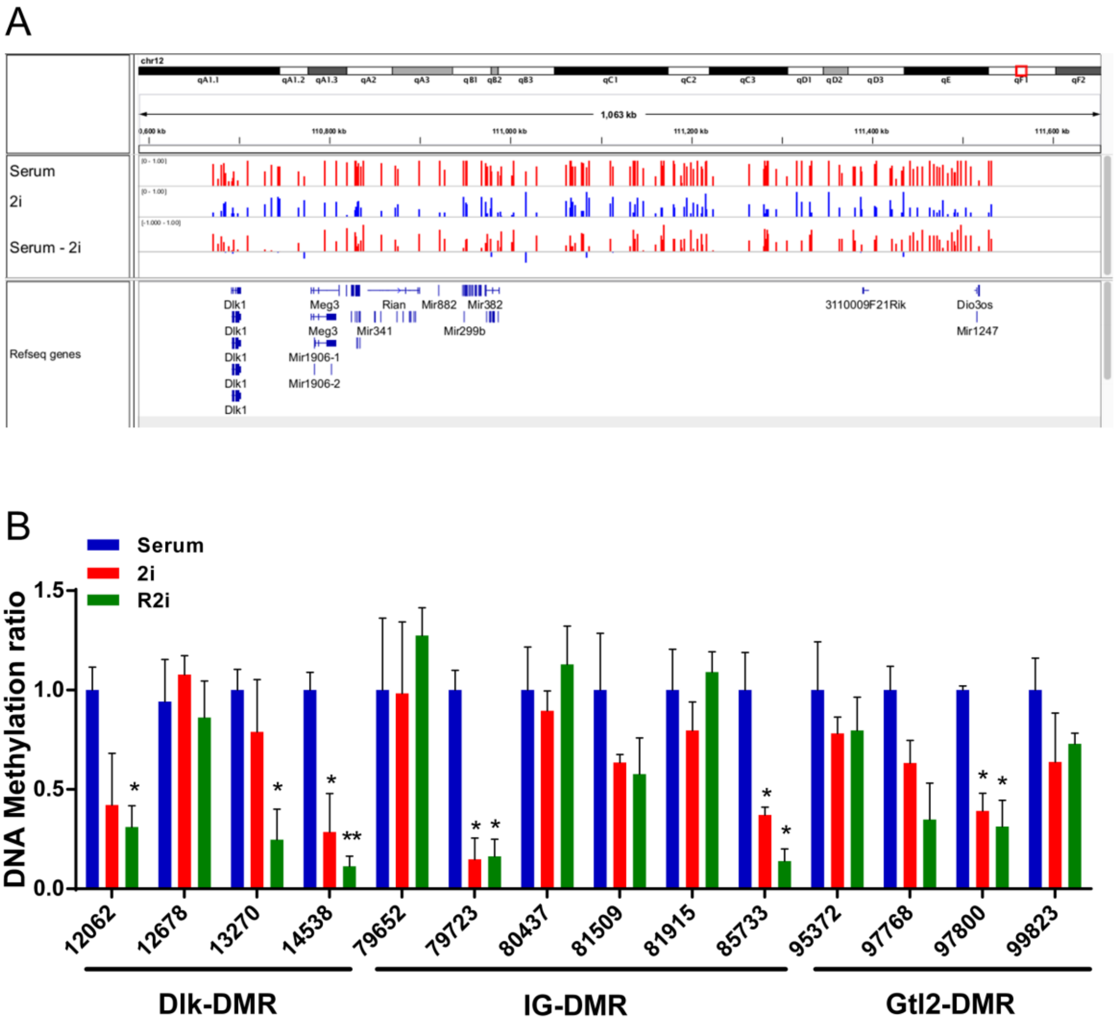

Figure S5: DNA methylation analysis of the *Dlk1-Dio3* locus, related to Figure 4

(A) IGV visualization of the methylation level of CpG dinucleotides at the *Dlk1-Dio3* locus for serum and 2i ESCs.

(B) Bar plots indicating the ratio of DNA methylation of the three DMRs at the *Dlk1-Dio3* locus in serum, 2i, and R2i cells as determined by qPCR. The X-axis numbers refer to the nucleotide positions in the *Dlk1-Dio3* locus corresponding to the site of enzymatic digestion. Data are shown as mean ± SD, n=3. *$p<0.05$.

A

miRNA	miR-541-5p	miR-410-3p	miR-381-3p
**Putative gene targets**	P4ha1<br>Prkag1<br>Acat1<br>Cpt1a<br>Myo1b<br>Ero1l<br>Mrpl15<br>Sbno1<br>Cdv3<br>Map4	Myl12b<br>Cbx3<br>Pcbp2<br>Srsf11	Map4<br>Rbm26<br>Idh2<br>Srsf1<br>Pura

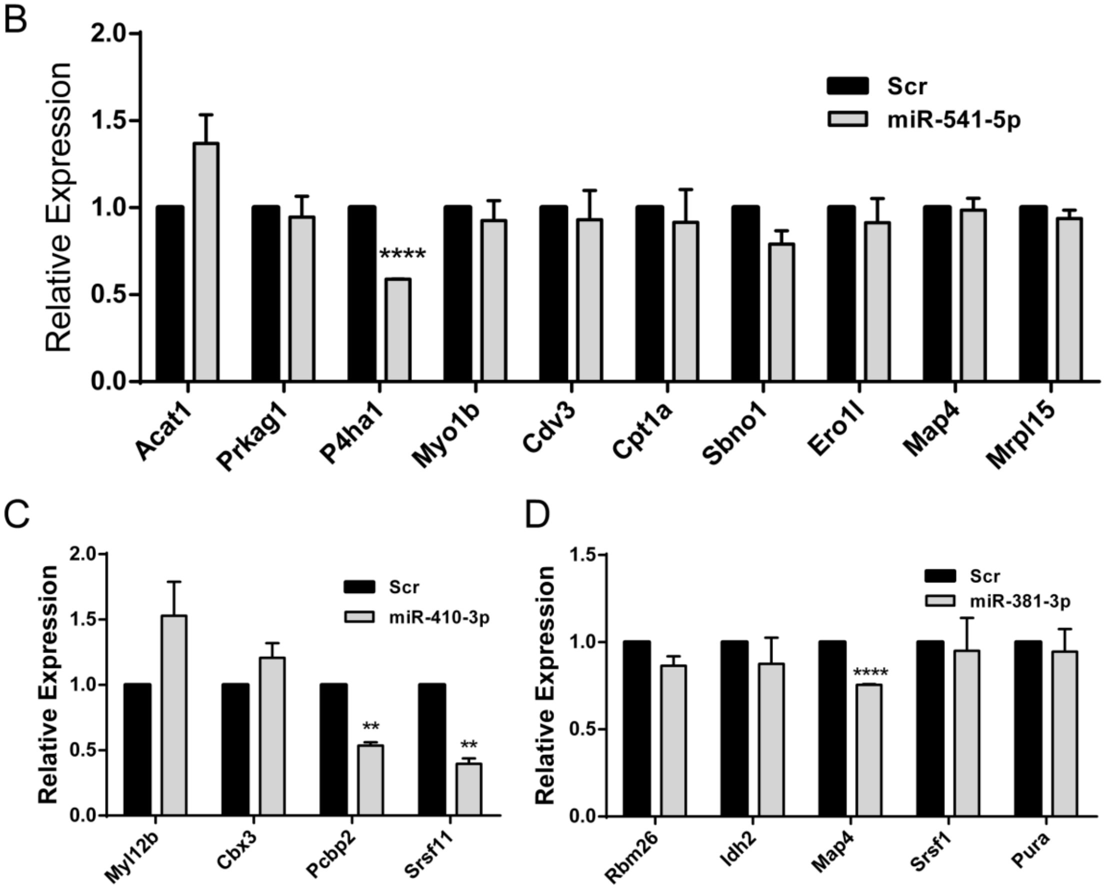

Figure S6: Putative gene targets of miR-541-5p, miR-410-3p, and miR-381-3p, related to Figures 5, 6, and 7

(A) List of genes that are both predicted to be targeted by the candidate miRNAs and downregulated at the protein level.

(B) Bar plot indicating expression of genes that are potentially regulated by miR-541-5p. Data are shown as mean ± SD, n=3. ****$p<0.0001$

(C) Bar plot indicating the expression of genes that are potentially regulated by miR-410-5p. Data are shown as mean ± SD, n=3. **$p<0.01$

(D) Bar plot indicating the expression of genes that are potentially regulated by miR-381-5p. Data are shown as mean ± SD, n=3. ****$p<0.0001$

Table S1. List of 2i-associated miRNAs (versus serum), related to Figure 3

miRNA ID	Log2 fold change	P value	Adjusted P value
mmu-let-7g-5p	1.60052	8.23E-08	5.08E-07
mmu-miR-1193-3p	4.177641	2.62E-08	1.68E-07
mmu-miR-1197-3p	5.020111	5.88E-10	4.37E-09
mmu-miR-127-3p	3.583421	3.93E-25	8.99E-24
mmu-miR-127-5p	4.201075	1.31E-10	1.06E-09
mmu-miR-134-5p	3.515577	3.96E-19	6.11E-18
mmu-miR-136-3p	3.884938	3.00E-31	2.05E-29
mmu-miR-136-5p	4.061845	4.40E-27	1.36E-25
mmu-miR-147-3p	1.535454	8.18E-06	3.85E-05
mmu-miR-147-5p	1.60728	2.46E-05	0.000105
mmu-miR-154-3p	3.66622	5.04E-07	2.83E-06
mmu-miR-154-5p	2.991621	0.000712	0.002363
mmu-miR-15a-3p	1.537571	0.002305	0.006524
mmu-miR-15a-5p	1.510485	6.02E-07	3.32E-06
mmu-miR-184-3p	2.532587	1.07E-08	7.10E-08
mmu-miR-199a-3p	1.23427	0.001959	0.005858
mmu-miR-218-5p	1.336842	0.00029	0.001045
mmu-miR-299a-3p	2.266181	2.80E-05	0.000119
mmu-miR-299a-5p	2.123809	0.002128	0.006253
mmu-miR-300-3p	4.318177	9.93E-34	1.02E-31
mmu-miR-301a-3p	1.41271	1.50E-07	8.88E-07
mmu-miR-301a-5p	1.8322	1.42E-05	6.39E-05
mmu-miR-301b-3p	1.367345	3.86E-06	1.89E-05
mmu-miR-301b-5p	2.461023	0.000719	0.002374
mmu-miR-302a-3p	1.087048	0.00168	0.005107
mmu-miR-302b-3p	1.661023	2.77E-07	1.61E-06
mmu-miR-302c-3p	2.050203	4.35E-07	2.46E-06
mmu-miR-3085-3p	2.79865	5.87E-05	0.00024
mmu-miR-323-3p	4.547108	1.65E-22	3.18E-21
mmu-miR-324-5p	1.766364	8.94E-08	5.46E-07
mmu-miR-329-3p	3.722546	9.44E-11	7.87E-10
mmu-miR-329-5p	3.579605	4.23E-11	3.63E-10
mmu-miR-337-3p	4.599271	1.73E-08	1.13E-07
mmu-miR-337-5p	3.924626	3.28E-19	5.18E-18
mmu-miR-341-3p	3.656685	6.23E-19	9.38E-18
mmu-miR-341-5p	2.958018	0.001512	0.004689
mmu-miR-367-3p	1.516567	0.000476	0.001631
mmu-miR-369-3p	4.156381	3.05E-26	7.53E-25
mmu-miR-369-5p	4.914904	1.02E-09	7.23E-09
mmu-miR-370-3p	3.742272	1.99E-18	2.73E-17
mmu-miR-370-5p	2.679494	0.00211	0.00623
mmu-miR-376a-3p	4.580739	1.14E-19	1.89E-18
mmu-miR-376a-5p	3.429957	1.52E-05	6.79E-05
mmu-miR-376b-3p	4.023984	2.28E-25	5.40E-24
mmu-miR-376b-5p	4.44467	5.64E-12	5.27E-11
mmu-miR-376c-3p	3.50518	1.27E-17	1.63E-16
mmu-miR-377-3p	5.239542	2.05E-14	2.18E-13
mmu-miR-379-3p	3.098211	2.40E-10	1.90E-09
mmu-miR-379-5p	3.211695	1.81E-18	2.56E-17
mmu-miR-380-3p	4.617464	9.99E-38	2.05E-35
mmu-miR-381-3p	3.087566	1.07E-15	1.22E-14
mmu-miR-382-3p	3.47989	4.67E-08	2.97E-07
mmu-miR-382-5p	4.122065	2.23E-14	2.34E-13
mmu-miR-409-3p	3.982859	1.75E-30	1.08E-28
mmu-miR-409-5p	3.59994	2.13E-11	1.97E-10
mmu-miR-410-3p	3.94237	2.77E-28	1.01E-26

mmu-miR-411-3p	3.736336	2.36E-11	2.14E-10
mmu-miR-411-5p	3.679751	1.41E-22	2.81E-21
mmu-miR-412-3p	3.425334	0.003376	0.009342
mmu-miR-412-5p	3.58605	8.97E-07	4.77E-06
mmu-miR-431-3p	5.073442	3.44E-17	4.24E-16
mmu-miR-431-5p	4.681326	2.70E-36	4.17E-34
mmu-miR-433-3p	4.323864	7.97E-18	1.07E-16
mmu-miR-434-3p	3.627581	2.45E-30	1.26E-28
mmu-miR-434-5p	3.652803	2.44E-29	1.07E-27
mmu-miR-451a	3.084706	0.000384	0.001347
mmu-miR-485-3p	3.173984	1.42E-14	1.54E-13
mmu-miR-485-5p	3.455305	5.24E-05	0.000218
mmu-miR-487b-3p	4.388037	8.64E-20	1.48E-18
mmu-miR-493-3p	3.385705	0.00217	0.006342
mmu-miR-494-3p	3.652028	2.64E-07	1.55E-06
mmu-miR-495-3p	4.388145	1.71E-28	7.03E-27
mmu-miR-496a-3p	4.051567	3.33E-12	3.21E-11
mmu-miR-499-5p	3.1575	5.88E-15	6.60E-14
mmu-miR-505-3p	2.738654	0.000355	0.00126
mmu-miR-539-5p	4.224067	7.66E-05	0.000305
mmu-miR-540-3p	3.293543	4.12E-17	4.99E-16
mmu-miR-540-5p	3.734214	5.37E-05	0.000222
mmu-miR-541-5p	3.620427	3.74E-34	4.62E-32
mmu-miR-543-3p	4.300728	2.78E-31	2.05E-29
mmu-miR-665-3p	4.217966	2.00E-28	7.72E-27
mmu-miR-666-3p	5.317512	2.55E-09	1.77E-08
mmu-miR-666-5p	5.06793	1.72E-26	5.04E-25
mmu-miR-667-3p	3.87041	1.19E-07	7.22E-07
mmu-miR-668-3p	4.3836	7.17E-07	3.85E-06
mmu-miR-673-3p	3.94442	3.60E-07	2.07E-06
mmu-miR-673-5p	3.728312	1.53E-19	2.49E-18
mmu-miR-679-5p	2.917934	5.74E-07	3.19E-06
mmu-miR-743b-3p	1.971061	0.000579	0.001942
mmu-miR-758-3p	4.209685	2.20E-30	1.23E-28
mmu-miR-871-3p	1.104186	0.002272	0.006461

Table S2. List of serum-associated miRNAs (versus 2i), related to Figure 3

miRNA ID	Log2 fold change	P value	Adjusted P value
mmu-miR-10a-5p	3.87726	1.98E-31	1.74E-29
mmu-miR-125a-5p	1.57999	5.80E-05	0.000239
mmu-miR-133a-3p	5.43483	6.30E-10	4.63E-09
mmu-miR-155-5p	1.60141	2.01E-06	1.01E-05
mmu-miR-181c-3p	1.39042	8.04E-05	0.000318
mmu-miR-181d-5p	1.24856	1.92E-05	8.36E-05
mmu-miR-1a-3p	6.16233	9.40E-10	6.74E-09
mmu-miR-200a-3p	1.16794	1.17E-05	5.38E-05
mmu-miR-200a-5p	1.64397	0.0001	0.000388
mmu-miR-200b-3p	1.75556	3.51E-10	2.70E-09
mmu-miR-203-3p	4.27738	2.95E-26	7.53E-25
mmu-miR-208b-3p	3.94806	2.92E-11	2.54E-10
mmu-miR-21a-3p	2.23079	4.51E-12	4.28E-11
mmu-miR-21a-5p	2.68639	1.95E-24	4.29E-23
mmu-miR-22-3p	1.58509	4.32E-07	2.46E-06
mmu-miR-23a-3p	1.46251	1.28E-05	5.81E-05
mmu-miR-24-2-5p	1.38923	0.000363	0.001279
mmu-miR-27a-3p	1.4175	1.87E-06	9.56E-06
mmu-miR-27a-5p	1.91032	0.000226	0.000829
mmu-miR-28a-3p	1.95939	3.01E-09	2.06E-08
mmu-miR-28a-5p	1.53412	5.09E-06	2.45E-05
mmu-miR-297a-3p	2.92385	9.40E-07	4.96E-06
mmu-miR-297a-5p	3.03081	6.31E-11	5.33E-10
mmu-miR-297b-3p	3.13206	6.90E-06	3.29E-05
mmu-miR-297b-5p	3.17505	1.62E-05	7.18E-05
mmu-miR-297c-3p	2.39433	0.000852	0.002752
mmu-miR-335-3p	1.73001	3.21E-09	2.18E-08
mmu-miR-34b-3p	2.73207	6.94E-06	3.29E-05
mmu-miR-34c-5p	2.72307	8.57E-17	1.02E-15
mmu-miR-362-3p	1.86082	6.34E-05	0.000258
mmu-miR-374b-3p	1.75378	0.000606	0.002022
mmu-miR-374b-5p	1.48947	1.95E-06	9.86E-06
mmu-miR-421-3p	1.59994	9.82E-07	5.13E-06
mmu-miR-466a-3p	2.4662	1.21E-05	5.55E-05
mmu-miR-466a-5p	2.96415	3.32E-05	0.00014
mmu-miR-466b-3p	2.90333	8.45E-18	1.11E-16
mmu-miR-466b-5p	2.70187	1.34E-14	1.47E-13
mmu-miR-466c-3p	2.67511	2.46E-11	2.20E-10
mmu-miR-466c-5p	2.87989	2.30E-17	2.89E-16
mmu-miR-466d-3p	3.48829	1.73E-06	8.92E-06
mmu-miR-466d-5p	3.25446	4.67E-14	4.72E-13
mmu-miR-466e-3p	2.47321	1.04E-05	4.84E-05
mmu-miR-466e-5p	2.3323	0.000535	0.001805
mmu-miR-466f	2.55241	0.000511	0.001744
mmu-miR-466f-5p	3.34727	1.71E-10	1.37E-09
mmu-miR-466h-3p	3.56794	0.000868	0.00279
mmu-miR-466h-5p	2.87567	2.23E-13	2.21E-12
mmu-miR-466n-3p	2.93058	3.55E-10	2.71E-09
mmu-miR-466n-5p	3.02528	2.61E-12	2.55E-11
mmu-miR-466o-5p	2.66791	4.64E-05	0.000195
mmu-miR-466p-3p	3.02355	6.31E-07	3.44E-06
mmu-miR-466p-5p	2.90491	0.000295	0.001058
mmu-miR-467a-3p	3.54528	1.81E-29	8.58E-28
mmu-miR-467a-5p	2.6155	9.87E-19	1.45E-17
mmu-miR-467b-5p	2.66652	2.09E-09	1.46E-08
mmu-miR-467c-5p	3.63232	4.89E-39	1.51E-36

mmu-miR-467d-3p	3.21852	5.72E-10	4.31E-09
mmu-miR-467d-5p	2.98514	2.25E-20	3.97E-19
mmu-miR-467e-3p	2.97482	6.90E-07	3.74E-06
mmu-miR-467e-5p	3.16111	2.84E-26	7.53E-25
mmu-miR-483-3p	4.62733	3.29E-10	2.57E-09
mmu-miR-483-5p	4.67976	6.90E-05	0.000278
mmu-miR-532-3p	1.45958	0.00263	0.007376
mmu-miR-532-5p	1.50469	5.91E-08	3.72E-07
mmu-miR-669a-3-3p	3.02171	1.83E-05	8.02E-05
mmu-miR-669a-3p	3.08277	1.80E-26	5.04E-25
mmu-miR-669a-5p	2.64805	3.30E-22	6.17E-21
mmu-miR-669b-5p	3.00143	1.83E-18	2.56E-17
mmu-miR-669c-5p	3.32342	1.02E-27	3.32E-26
mmu-miR-669d-5p	3.4231	3.75E-22	6.81E-21
mmu-miR-669e-3p	2.58227	0.002179	0.006342
mmu-miR-669e-5p	3.66885	2.05E-23	4.36E-22
mmu-miR-669f-3p	2.20867	9.07E-05	0.000356
mmu-miR-669f-5p	2.71234	2.67E-11	2.36E-10
mmu-miR-669h-5p	3.24075	6.30E-09	4.23E-08
mmu-miR-669l-3p	3.5162	0.000971	0.003088
mmu-miR-669l-5p	3.57413	8.02E-28	2.75E-26
mmu-miR-669m-5p	3.02974	3.38E-23	6.95E-22
mmu-miR-669o-3p	3.07816	1.31E-10	1.06E-09
mmu-miR-669o-5p	2.48472	4.53E-14	4.66E-13
mmu-miR-669p-3p	2.5773	0.001892	0.005696
mmu-miR-669p-5p	2.62983	5.78E-16	6.73E-15
mmu-miR-674-3p	1.72256	1.16E-06	6.00E-06
mmu-miR-674-5p	1.53656	0.000121	0.000459
mmu-miR-675-3p	4.28965	0.000343	0.001222
mmu-miR-6944-3p	3.30547	6.42E-10	4.66E-09
mmu-miR-92a-2-5p	1.85015	4.20E-06	2.04E-05
mmu-miR-99b-5p	1.04178	0.002262	0.00646

Table S3. List of R2i-associated miRNAs (versus serum), related to Figure 3

miRNA ID	Log2 Fold Change	P value	Adjusted p value
mmu-let-7g-5p	1.026505	0.000639	0.002435
mmu-miR-1193-3p	3.682127	1.18E-06	6.81E-06
mmu-miR-1197-3p	4.557951	2.29E-08	1.63E-07
mmu-miR-1247-3p	2.023308	0.002368	0.008161
mmu-miR-127-3p	3.660928	3.71E-26	2.00E-24
mmu-miR-127-5p	4.250293	9.30E-11	9.25E-10
mmu-miR-134-5p	4.09155	2.44E-25	1.08E-23
mmu-miR-136-3p	3.962237	2.14E-32	4.39E-30
mmu-miR-136-5p	3.563506	4.94E-21	1.53E-19
mmu-miR-147-3p	1.469419	2.21E-05	0.000107
mmu-miR-147-5p	1.87855	9.10E-07	5.35E-06
mmu-miR-153-3p	2.129974	5.11E-06	2.72E-05
mmu-miR-154-3p	3.47848	2.18E-06	1.22E-05
mmu-miR-154-5p	3.404436	0.00012	0.000529
mmu-miR-15a-5p	1.125982	0.000207	0.000882
mmu-miR-184-3p	1.974559	1.02E-05	5.27E-05
mmu-miR-187-3p	1.262837	0.00274	0.009379
mmu-miR-218-5p	1.115704	0.002751	0.009379
mmu-miR-299a-3p	2.915455	6.74E-08	4.57E-07
mmu-miR-299a-5p	2.502315	0.000304	0.001259
mmu-miR-300-3p	4.095046	2.00E-30	2.46E-28
mmu-miR-300-5p	3.300759	0.000978	0.003553
mmu-miR-301a-3p	1.169306	1.39E-05	7.03E-05
mmu-miR-301a-5p	1.428146	0.000847	0.00315
mmu-miR-301b-3p	1.056701	0.000367	0.001499
mmu-miR-302a-3p	2.172296	3.26E-10	2.96E-09
mmu-miR-302a-5p	2.010583	2.38E-06	1.32E-05
mmu-miR-302b-3p	2.494541	1.17E-14	1.68E-13
mmu-miR-302c-3p	3.070571	3.19E-14	4.38E-13
mmu-miR-302c-5p	1.992109	6.53E-05	0.000301
mmu-miR-302d-3p	2.123171	1.07E-08	7.87E-08
mmu-miR-302d-5p	2.053181	6.84E-05	0.000313
mmu-miR-3072-3p	3.127403	0.00189	0.006625
mmu-miR-323-3p	4.322572	2.48E-20	6.64E-19
mmu-miR-324-5p	1.074954	0.001285	0.004584
mmu-miR-329-3p	3.510127	1.31E-09	1.08E-08
mmu-miR-329-5p	3.44769	2.83E-10	2.61E-09
mmu-miR-337-3p	4.820792	3.72E-09	2.98E-08
mmu-miR-337-5p	4.003505	8.28E-20	1.89E-18
mmu-miR-341-3p	3.683565	3.93E-19	8.35E-18

mmu-miR-3535	1.127635	0.000149	0.000646
mmu-miR-367-3p	2.672808	6.26E-10	5.37E-09
mmu-miR-369-3p	3.991549	3.37E-24	1.09E-22
mmu-miR-369-5p	4.880154	1.52E-09	1.23E-08
mmu-miR-370-3p	3.634605	2.37E-17	4.29E-16
mmu-miR-376a-3p	4.364697	7.54E-18	1.41E-16
mmu-miR-376a-5p	2.857094	0.000374	0.001519
mmu-miR-376b-3p	3.611718	1.45E-20	4.26E-19
mmu-miR-376b-5p	4.130688	2.00E-10	1.93E-09
mmu-miR-376c-3p	3.589962	2.77E-18	5.51E-17
mmu-miR-377-3p	4.845733	2.00E-12	2.29E-11
mmu-miR-379-3p	3.257469	3.24E-11	3.27E-10
mmu-miR-379-5p	3.872263	3.89E-26	2.00E-24
mmu-miR-380-3p	4.230026	9.08E-32	1.40E-29
mmu-miR-381-3p	3.516498	6.79E-20	1.66E-18
mmu-miR-382-3p	3.336479	2.02E-07	1.31E-06
mmu-miR-382-5p	4.237989	5.05E-15	7.98E-14
mmu-miR-409-3p	3.950542	5.72E-30	5.88E-28
mmu-miR-409-5p	3.403733	3.37E-10	3.01E-09
mmu-miR-410-3p	3.716682	2.78E-25	1.14E-23
mmu-miR-411-3p	3.801019	1.32E-11	1.40E-10
mmu-miR-411-5p	3.831339	2.92E-24	1.06E-22
mmu-miR-412-5p	2.884534	9.87E-05	0.000444
mmu-miR-431-3p	4.841877	1.12E-15	1.82E-14
mmu-miR-431-5p	4.202259	2.05E-29	1.58E-27
mmu-miR-433-3p	3.853132	2.86E-14	4.01E-13
mmu-miR-434-3p	3.584975	1.39E-29	1.23E-27
mmu-miR-434-5p	3.880914	7.24E-33	2.23E-30
mmu-miR-451a	3.925137	5.86E-06	3.09E-05
mmu-miR-470-5p	1.584688	9.93E-05	0.000444
mmu-miR-485-3p	3.212024	9.05E-15	1.36E-13
mmu-miR-485-5p	3.642984	2.15E-05	0.000104
mmu-miR-487b-3p	4.08367	3.53E-17	5.88E-16
mmu-miR-494-3p	4.175452	4.02E-09	3.18E-08
mmu-miR-495-3p	4.045337	3.15E-24	1.08E-22
mmu-miR-496a-3p	3.940805	1.65E-11	1.73E-10
mmu-miR-499-5p	2.932804	5.69E-13	6.75E-12
mmu-miR-5121	1.247815	0.000174	0.000751
mmu-miR-539-5p	4.292943	6.19E-05	0.000287
mmu-miR-540-3p	3.529823	2.44E-19	5.37E-18
mmu-miR-540-5p	3.211803	0.000588	0.002254
mmu-miR-541-5p	3.900606	2.31E-39	1.43E-36

mmu-miR-543-3p	4.055485	8.85E-28	6.07E-26
mmu-miR-665-3p	4.128172	3.84E-27	2.37E-25
mmu-miR-666-3p	4.152546	4.29E-06	2.32E-05
mmu-miR-666-5p	4.418284	2.93E-20	7.53E-19
mmu-miR-667-3p	3.599379	1.04E-06	6.08E-06
mmu-miR-668-3p	3.157516	0.000451	0.001795
mmu-miR-673-3p	4.02305	2.34E-07	1.49E-06
mmu-miR-673-5p	3.770622	7.01E-20	1.66E-18
mmu-miR-677-5p	1.303209	2.06E-05	0.000102
mmu-miR-679-5p	2.899979	8.11E-07	4.81E-06
mmu-miR-758-3p	3.85022	1.74E-25	8.26E-24
mmu-miR-871-3p	1.360755	0.000175	0.000751
mmu-miR-874-3p	2.629872	2.56E-07	1.61E-06

Table S4. List of serum-associated miRNAs (versus R2i), related to Figure 3

miRNA ID	Log2 Fold Change	P value	Adjusted p value
mmu-let-7e-5p	1.52341	3.01E-05	0.000144
mmu-miR-10a-5p	2.15682	2.14E-11	2.20E-10
mmu-miR-125a-3p	1.65385	0.000949	0.003484
mmu-miR-125a-5p	1.9674	5.77E-07	3.49E-06
mmu-miR-125b-1-3p	1.79227	0.000477	0.001888
mmu-miR-133a-3p	3.97152	2.97E-07	1.85E-06
mmu-miR-142a-3p	2.19719	7.19E-08	4.82E-07
mmu-miR-142a-5p	2.43938	6.36E-12	7.01E-11
mmu-miR-146a-5p	2.04349	1.76E-10	1.72E-09
mmu-miR-152-5p	1.53467	0.000979	0.003553
mmu-miR-155-5p	1.48648	1.25E-05	6.38E-05
mmu-miR-181c-3p	2.60936	4.29E-13	5.29E-12
mmu-miR-181c-5p	2.14744	6.17E-15	9.52E-14
mmu-miR-181d-5p	2.4829	2.76E-17	4.87E-16
mmu-miR-1a-3p	2.57797	0.000411	0.001645
mmu-miR-200a-3p	1.05069	8.20E-05	0.000372
mmu-miR-200a-5p	1.5657	0.00026	0.001086
mmu-miR-200b-3p	1.40789	4.94E-07	3.05E-06
mmu-miR-203-3p	3.46253	1.70E-18	3.51E-17
mmu-miR-208b-3p	3.73695	6.26E-10	5.37E-09
mmu-miR-21a-3p	2.51088	1.17E-14	1.68E-13
mmu-miR-21a-5p	2.43637	2.25E-20	6.30E-19
mmu-miR-22-3p	1.50959	1.49E-06	8.49E-06
mmu-miR-23a-3p	1.94247	8.39E-09	6.31E-08
mmu-miR-24-2-5p	2.21037	2.10E-08	1.51E-07
mmu-miR-27a-3p	1.4747	7.51E-07	4.50E-06
mmu-miR-27a-5p	2.53934	2.99E-06	1.63E-05
mmu-miR-28a-3p	1.25755	0.000136	0.000596
mmu-miR-28a-5p	1.23602	0.00025	0.00105
mmu-miR-297a-3p	2.07167	0.0004	0.001613
mmu-miR-297a-5p	2.12757	2.79E-06	1.54E-05
mmu-miR-297b-3p	2.38195	0.000488	0.001916
mmu-miR-297c-3p	2.26393	0.001958	0.006826
mmu-miR-31-5p	1.68844	7.88E-08	5.23E-07
mmu-miR-335-3p	1.1739	5.87E-05	0.000274
mmu-miR-34c-5p	1.08604	0.000826	0.003087
mmu-miR-362-3p	1.58077	0.000761	0.002879
mmu-miR-374b-5p	1.2173	0.000106	0.000469
mmu-miR-421-3p	1.34879	3.90E-05	0.000185
mmu-miR-466a-3p	2.07749	0.000245	0.001034

mmu-miR-466b-3p	2.10253	3.74E-10	3.29E-09
mmu-miR-466b-5p	2.12545	1.31E-09	1.08E-08
mmu-miR-466c-3p	1.78562	6.25E-06	3.27E-05
mmu-miR-466c-5p	2.14839	2.18E-10	2.07E-09
mmu-miR-466d-3p	2.1528	0.001796	0.006333
mmu-miR-466d-5p	2.43989	9.38E-09	6.98E-08
mmu-miR-466e-3p	1.69531	0.002196	0.00761
mmu-miR-466f	2.60037	0.000581	0.00224
mmu-miR-466f-5p	2.15427	1.83E-05	9.08E-05
mmu-miR-466h-5p	2.13618	3.93E-08	2.72E-07
mmu-miR-466n-3p	2.053	7.97E-06	4.13E-05
mmu-miR-466n-5p	2.22737	1.79E-07	1.17E-06
mmu-miR-466p-3p	1.9775	0.000809	0.003044
mmu-miR-466p-5p	2.64913	0.001106	0.003992
mmu-miR-467a-3p	2.63471	3.16E-17	5.42E-16
mmu-miR-467a-5p	1.80709	9.20E-10	7.77E-09
mmu-miR-467b-5p	1.55055	0.000349	0.001435
mmu-miR-467c-5p	2.82503	2.37E-24	9.15E-23
mmu-miR-467d-3p	2.8617	4.48E-08	3.07E-07
mmu-miR-467d-5p	2.28022	1.24E-12	1.45E-11
mmu-miR-467e-3p	2.60333	1.58E-05	7.92E-05
mmu-miR-467e-5p	2.24406	3.47E-14	4.65E-13
mmu-miR-483-3p	3.21729	1.57E-06	8.86E-06
mmu-miR-532-5p	1.59101	1.19E-08	8.62E-08
mmu-miR-669a-3p	2.51499	3.64E-18	7.03E-17
mmu-miR-669a-5p	1.85917	9.65E-12	1.04E-10
mmu-miR-669b-5p	2.13996	2.67E-10	2.50E-09
mmu-miR-669c-5p	2.28157	4.73E-14	6.21E-13
mmu-miR-669d-5p	2.40782	4.06E-12	4.56E-11
mmu-miR-669e-5p	2.62906	2.32E-13	2.92E-12
mmu-miR-669f-3p	1.96794	0.000567	0.002206
mmu-miR-669f-5p	1.83346	4.83E-06	2.59E-05
mmu-miR-669h-5p	2.16641	5.79E-05	0.000273
mmu-miR-669l-5p	2.41132	5.44E-14	7.00E-13
mmu-miR-669m-5p	2.1922	5.20E-13	6.29E-12
mmu-miR-669o-3p	2.37317	5.37E-07	3.28E-06
mmu-miR-669o-5p	1.90913	6.70E-09	5.17E-08
mmu-miR-669p-3p	3.00953	0.000568	0.002206
mmu-miR-669p-5p	1.8738	6.99E-09	5.32E-08
mmu-miR-674-3p	2.11798	5.96E-09	4.66E-08
mmu-miR-674-5p	1.73585	2.09E-05	0.000102
mmu-miR-6944-3p	2.98412	3.18E-08	2.23E-07

mmu-miR-99b-3p	1.5231	0.000867	0.003202
mmu-miR-99b-5p	1.77098	2.16E-07	1.39E-06

Table S5. List of 2i- and R2i-associated miRNAs, related to Figure 3

miRNA ID	Log2 Fold Change	P value	Adjusted p value
**2i-associated miRNAs (versus R2i)**			
mmu-miR-142a-3p	1.92114	2.45E-06	0.000134
mmu-miR-142a-5p	2.62459	1.41E-13	3.48E-11
mmu-miR-146a-5p	1.23012	0.000126	0.004782
mmu-miR-152-3p	1.86974	2.18E-08	2.68E-06
mmu-miR-152-5p	2.44772	8.70E-08	8.57E-06
mmu-miR-181c-5p	1.44296	1.60E-07	1.31E-05
mmu-miR-181d-5p	1.23435	2.67E-05	0.001187
mmu-miR-211-3p	4.5873	2.63E-10	4.32E-08
mmu-miR-211-5p	4.56645	2.23E-39	1.10E-36
**R2i-associated miRNAs (versus 2i)**			
mmu-miR-10a-5p	1.720439	4.07E-07	2.87E-05
mmu-miR-302a-5p	1.776923	2.89E-05	0.001187
mmu-miR-302d-3p	1.412517	0.000141	0.00496
mmu-miR-34c-5p	1.637035	6.20E-07	3.82E-05
mmu-miR-3535	1.325008	8.27E-06	0.000408
mmu-miR-6240	1.335964	0.000265	0.00872

Table S6. List of serum- and ground state-associated miRNAs, related to Figure 3

Ground state-associated miRNAs (versus serum)			Serum-associated miRNAs (versus ground state)		
**miRNA ID**	**Log2 Fold Change**	**Adjusted p value**	**miRNA ID**	**Log2 Fold Change**	**Adjusted p value**
mmu-let-7g-5p	1.346466	0.000227	mmu-miR-10a-5p	2.71438	1.40E-05
mmu-miR-1193-3p	4.223692	1.53E-07	mmu-miR-125a-3p	1.45761	0.001066
mmu-miR-1197-3p	5.213232	2.83E-08	mmu-miR-125a-5p	1.76216	0.000277
mmu-miR-1247-3p	2.047209	0.001109	mmu-miR-133a-3p	4.77846	1.16E-09
mmu-miR-127-3p	3.641083	1.23E-22	mmu-miR-149-5p	1.0969	0.003174
mmu-miR-127-5p	4.411212	1.05E-10	mmu-miR-155-5p	1.55942	6.57E-08
mmu-miR-134-5p	3.863615	9.43E-18	mmu-miR-181a-2-3p	2.07394	0.006915
mmu-miR-136-3p	3.950312	8.35E-31	mmu-miR-181c-3p	1.87785	0.000973
mmu-miR-136-5p	3.868125	1.29E-20	mmu-miR-181d-5p	1.72886	0.001278
mmu-miR-147-3p	1.519911	7.51E-07	mmu-miR-200a-3p	1.11462	3.42E-06
mmu-miR-147-5p	1.769462	3.53E-07	mmu-miR-200a-5p	1.62601	4.12E-05
mmu-miR-154-3p	3.77435	1.04E-06	mmu-miR-200b-3p	1.57816	1.68E-07
mmu-miR-154-5p	3.540456	0.00019	mmu-miR-203-3p	3.85143	2.67E-20
mmu-miR-15a-3p	1.40228	0.003559	mmu-miR-208b-3p	3.93947	8.45E-13
mmu-miR-15a-5p	1.336776	9.95E-05	mmu-miR-21a-3p	2.37758	1.72E-12
mmu-miR-16-1-3p	1.007219	0.009531	mmu-miR-21a-5p	2.56769	2.36E-24
mmu-miR-184-3p	2.2992	3.49E-06	mmu-miR-22-3p	1.55562	3.42E-06

mmu-miR-187-3p	1.175675	0.00399	mmu-miR-23a-3p	1.69033	2.39E-05
mmu-miR-1981-5p	1.555043	0.002766	mmu-miR-24-2-5p	1.74394	0.000958
mmu-miR-199a-3p	1.20635	0.003041	mmu-miR-27a-3p	1.45499	8.59E-07
mmu-miR-218-5p	1.246712	0.000324	mmu-miR-27a-5p	2.20878	3.72E-05
mmu-miR-299a-3p	2.684175	1.88E-06	mmu-miR-28a-3p	1.57158	0.000144
mmu-miR-299a-5p	2.447515	0.000307	mmu-miR-28a-5p	1.38864	6.54E-05
mmu-miR-300-3p	4.242033	1.42E-28	mmu-miR-297a-3p	2.51336	1.56E-05
mmu-miR-300-5p	3.341582	0.004116	mmu-miR-297a-5p	2.55356	9.07E-08
mmu-miR-301a-3p	1.302725	1.28E-06	mmu-miR-297b-3p	2.84516	2.98E-06
mmu-miR-301a-5p	1.672406	5.31E-05	mmu-miR-297b-5p	2.5682	0.000793
mmu-miR-301b-3p	1.227613	0.000166	mmu-miR-297c-3p	2.45532	5.69E-05
mmu-miR-301b-5p	2.362827	0.001111	mmu-miR-335-3p	1.42225	0.000136
mmu-miR-302a-3p	1.729754	0.001358	mmu-miR-34b-3p	1.99459	0.003108
mmu-miR-302b-3p	2.140033	2.14E-06	mmu-miR-362-3p	1.75341	1.11E-05
mmu-miR-302c-3p	2.657353	1.20E-06	mmu-miR-374b-3p	1.68092	0.000199
mmu-miR-3072-3p	3.133091	0.00741	mmu-miR-374b-5p	1.35723	1.36E-05
mmu-miR-323-3p	4.52449	9.71E-22	mmu-miR-421-3p	1.47989	1.38E-05
mmu-miR-324-3p	1.247244	0.004731	mmu-miR-466a-3p	2.33663	9.17E-07
mmu-miR-324-5p	1.466346	0.000442	mmu-miR-466a-5p	2.52089	0.000161
mmu-miR-329-3p	3.736309	2.27E-10	mmu-miR-466b-3p	2.45995	4.45E-09
mmu-miR-329-5p	3.622638	2.37E-12	mmu-miR-466b-5p	2.4044	6.79E-11
mmu-miR-337-3p	5.131488	3.60E-08	mmu-miR-466c-3p	2.18635	2.73E-06
mmu-miR-337-5p	4.033849	2.20E-22	mmu-miR-466c-5p	2.47573	2.17E-09
mmu-miR-341-3p	3.708818	1.16E-16	mmu-miR-466d-3p	2.78809	0.000102
mmu-miR-341-5p	2.819602	0.008834	mmu-miR-466d-5p	2.83411	1.43E-10
mmu-miR-367-3p	2.21524	0.000268	mmu-miR-466e-3p	2.09689	8.33E-05
mmu-miR-369-3p	4.125175	2.00E-24	mmu-miR-466e-5p	2.25502	0.000118
mmu-miR-369-5p	5.316992	6.83E-09	mmu-miR-466f	2.71802	2.78E-05
mmu-miR-370-3p	3.742474	2.76E-17	mmu-miR-466f-5p	2.67411	2.41E-06
mmu-miR-370-5p	2.668108	0.004692	mmu-miR-466h-3p	3.69355	0.001068
mmu-miR-376a-3p	4.589456	6.72E-20	mmu-miR-466h-5p	2.48919	1.83E-09
mmu-miR-376a-5p	3.399585	6.99E-05	mmu-miR-466k	2.34043	0.006553
mmu-miR-376b-3p	3.872204	2.61E-21	mmu-miR-466n-3p	2.46922	9.11E-07
mmu-miR-376b-5p	4.48077	5.51E-11	mmu-miR-466n-5p	2.61288	5.01E-09
mmu-miR-376c-3p	3.603797	3.68E-22	mmu-miR-466o-5p	2.18533	0.000515
mmu-miR-377-3p	5.328743	1.56E-12	mmu-miR-466p-3p	2.46707	0.00017
mmu-miR-379-3p	3.244779	4.25E-11	mmu-miR-466p-5p	2.95751	5.31E-05
mmu-miR-379-5p	3.591239	4.87E-16	mmu-miR-467a-3p	3.01199	2.93E-12
mmu-miR-380-3p	4.474326	2.03E-30	mmu-miR-467a-5p	2.15512	1.34E-07
mmu-miR-381-3p	3.32822	1.86E-13	mmu-miR-467b-5p	2.02111	0.000164
mmu-miR-382-3p	3.570635	6.42E-09	mmu-miR-467c-5p	3.16445	4.75E-16
mmu-miR-382-5p	4.298436	1.94E-15	mmu-miR-467d-3p	3.10989	1.27E-12

mmu-miR-409-3p	3.995605	2.48E-28	mmu-miR-467d-5p	2.60192	6.64E-12
mmu-miR-409-5p	3.609228	2.63E-12	mmu-miR-467e-3p	2.88294	8.68E-09
mmu-miR-410-3p	3.858489	4.31E-23	mmu-miR-467e-5p	2.62303	6.26E-10
mmu-miR-411-3p	3.893429	3.42E-13	mmu-miR-483-3p	3.90738	1.10E-08
mmu-miR-411-5p	3.796325	3.71E-23	mmu-miR-483-5p	4.2486	0.000928
mmu-miR-412-3p	4.300049	0.006055	mmu-miR-532-3p	1.44713	0.000942
mmu-miR-412-5p	3.474198	7.43E-06	mmu-miR-532-5p	1.55719	6.79E-11
mmu-miR-431-3p	5.14734	5.45E-16	mmu-miR-669a-3-3p	2.50769	0.000227
mmu-miR-431-5p	4.49795	8.73E-27	mmu-miR-669a-3p	2.77868	7.00E-17
mmu-miR-433-3p	4.197099	2.10E-15	mmu-miR-669a-5p	2.19413	2.08E-08
mmu-miR-433-5p	2.95281	0.005417	mmu-miR-669b-5p	2.51493	2.68E-09
mmu-miR-434-3p	3.633688	5.23E-33	mmu-miR-669c-5p	2.69534	6.17E-09
mmu-miR-434-5p	3.794216	1.52E-29	mmu-miR-669d-5p	2.82857	9.73E-10
mmu-miR-451a	3.866863	9.67E-05	mmu-miR-669e-3p	2.74839	0.000415
mmu-miR-470-5p	1.359331	0.002239	mmu-miR-669e-5p	3.06581	3.55E-11
mmu-miR-485-3p	3.243292	7.72E-18	mmu-miR-669f-3p	2.15046	6.76E-06
mmu-miR-485-5p	3.886438	2.57E-05	mmu-miR-669f-5p	2.23105	9.94E-07
mmu-miR-487b-3p	4.331313	2.22E-18	mmu-miR-669h-5p	2.67599	1.34E-06
mmu-miR-494-3p	4.105908	1.56E-07	mmu-miR-669l-3p	2.9387	0.006154
mmu-miR-495-3p	4.282929	1.75E-27	mmu-miR-669l-5p	2.86718	3.80E-09
mmu-miR-496a-3p	4.141768	3.78E-13	mmu-miR-669m-5p	2.55116	3.50E-10
mmu-miR-499-5p	3.08594	2.12E-14	mmu-miR-669o-3p	2.73991	1.46E-09
mmu-miR-505-3p	2.56491	0.001681	mmu-miR-669o-5p	2.18384	1.93E-09
mmu-miR-539-5p	5.241534	0.000221	mmu-miR-669p-3p	2.96495	7.74E-05
mmu-miR-540-3p	3.459006	1.18E-19	mmu-miR-669p-5p	2.21086	4.34E-08
mmu-miR-540-5p	3.925899	0.000189	mmu-miR-674-3p	1.9181	1.13E-08
mmu-miR-541-3p	4.375227	0.004831	mmu-miR-674-5p	1.64858	8.56E-06
mmu-miR-541-5p	3.784097	1.12E-33	mmu-miR-675-3p	4.11139	0.001253
mmu-miR-543-3p	4.233375	5.23E-33	mmu-miR-6944-3p	3.22073	2.08E-11
mmu-miR-665-3p	4.229401	8.98E-32	mmu-miR-92a-3p	1.14356	0.004217
mmu-miR-666-3p	5.330333	2.14E-06	mmu-miR-99b-3p	1.37392	0.001109
mmu-miR-666-5p	4.860263	1.56E-20	mmu-miR-99b-5p	1.35383	0.004856
mmu-miR-667-3p	3.98145	1.53E-07			
mmu-miR-668-3p	4.234536	7.74E-05			
mmu-miR-673-3p	4.259766	3.26E-07			
mmu-miR-673-5p	3.790264	5.95E-17			
mmu-miR-679-5p	3.014489	2.14E-08			
mmu-miR-743b-3p	1.798647	0.001278			
mmu-miR-758-3p	4.080903	5.22E-27			
mmu-miR-871-3p	1.247032	0.00108			
mmu-miR-874-3p	2.032867	0.006821			
mmu-miR-98-5p	1.050864	0.005209			

Table S7: List of predicted mRNA targets used for gene ontology and pathway analysis, related to Figures 3 and 5

Table S8. Potential biological pathways inhibited by miR-541-5p, miR-410-3p, and miR-381-3p (DAVID analysis), related to Figure 5

**miR-541-5p targets**	
**GO term**	**P value**
Chordate embryonic development	1.2E-3
Muscle tissue development	9.9E-2
Transcription regulation	3.9E-3
Embryonic limb morphogenesis	5.5E-2
Nucleotide-binding	1.5E-2
Adult locomotor behavior	2.4E-2
Chromatin binding	2.9E-3
Pattern specification process	9.9E-2
**miR-410-3p targets**	
Transcription regulation	1.7E-22
Vasculature development	7.8E-9
Respiratory system development	9.6E-9
Neuron differentiation	6.8E-9
Cell migration	1.0E-6
Wnt signaling pathway	6.5E-7
Sensory organ development	2.3E-7
Chordate embryonic development	2.0E-5
**miR-381-3p targets**	
Transcription regulation	4.9E-26
Pattern specification process	1.1E-8
Cell morphogenesis during differentiation	6.1E-10
Neuron differentiation	1.6E-7
Embryonic morphogenesis	2.7E-7
Nucleoplasm	1.6E-9
Sensory organ development	5.2E-8
Wnt signaling pathway	1.7E-5

II. Supplemental Experimental Procedures

Cell culture

RB18 and RB20 mouse ESC lines were maintained at 37˚C in 5% CO_2, cultivated on gelatinized tissue-culture plates and dishes (Sigma-Aldrich), and passaged every other day. Serum ESCs were cultured in Knockout™ DMEM (Invitrogen) supplemented with 15% ES-qualified fetal bovine serum (HyClone), 0.1 mM β-mercaptoethanol (Sigma-Aldrich), 0.1 mM non-essential amino acids (Invitrogen), 2 mM L-glutamine (Invitrogen), 100 U/ml penicillin, 100 μg/ml streptomycin (Invitrogen), and 1000 U/ml mouse LIF (mLIF, Royan Biotech). 2i and R2i cells were cultured in N2B27 media that consisted of Neurobasal® medium and DMEM/F-12 (both from Invitrogen) at a 1:1 ratio, 1% N2 supplement (Invitrogen), 1% B27 supplement (Invitrogen), 0.1 mM β-mercaptoethanol, 5 mg/ml BSA (Invitrogen), 2 mM L-glutamine, 0.1 mM non-essential amino acids, 100 U/ml penicillin, 100 μg/ml streptomycin, and 1000 U/ml mLIF. Small-molecule chemicals used in 2i and R2i cultures were added at the following concentrations: 1 μM PD0325901 (Stemgent), 10 μM SB431542 (Sigma-Aldrich), and 3 μM CHIR99021 (Stemgent).

For spontaneous differentiation through embryoid body (EB) formation, the ESCs were trypsinized and suspended in serum ESC medium without LIF on non-adherent bacterial petri dishes at 1.5×10^5 cells/ml. Five days after induction of EB formation, a fraction of the EBs was transferred onto gelatinized 24-well plates for alkaline phosphatase (AP) staining on day 7. Remaining EBs were allowed to differentiate in suspension and harvested on day 7 for qRT-PCR analysis of pluripotency- and differentiation-associated genes. Media were changed every other day.

Small RNA delivery

RB20 ESCs were seeded 24 h prior to transfection on gelatinized plates in ESC medium, then transfected with 100 nM of miRNA mimics (Dharmacon, miRIDIAN microRNA mimics, Thermo Fisher Scientific) or 50 nM of miRNA Power Inhibitors (Exiqon) according to the manufacturer's instructions. Prior to transfection, the miRNA mimics or the scrambled small RNA control were diluted in serum-free DMEM/F-12 (Invitrogen) and incubated with the diluted DharmaFECT1 transfection reagent (Dharmacon, Thermo Fisher Scientific) in serum-free DMEM/F-12 for 20 min at RT. miRNA inhibitors were directly added to the cell culture media without any reagent, as recommended by the manufacturer. After miRNA/anti-miRNA delivery, cells were incubated at 37°C in a humidified incubator containing 5% CO_2 prior to analysis. Cell-based and molecular assays were performed in triplicate (three biological replicates). Data are shown as the mean ± SD.

Cell viability assays

MTS proliferation assay

20 µl of the cell proliferation (MTS) reagent was directly added to the wells (96-well plate) according to the vendor's instructions (Promega) after removal of medium. Cells were incubated in a humidified incubator at 37°C for 1-3 h. Cell viability was measured by determining absorbance at 495 nm on a Multiskan MCC microplate reader (Thermo Fisher Scientific).

Live/dead viability assay

A working solution containing 0.1 µM calcein acetoxymethyl ester (calcein AM) and 0.1 µM ethidium homodimer-1 in PBS was prepared according to the instructions in the Live/Dead®

Viability/Cytotoxicity Kit for Mammalian Cells (Molecular Probes). Cells were incubated with the reagent in a humidified incubator at RT for 30-60 min. After removal of the reagent, cells were washed with PBS and viewed under fluorescence microscope (Olympus, IX71).

RNA isolation and qRT-PCR

Total RNA was extracted using either the miRVana™ miRNA Isolation Kit (Invitrogen) or miRNeasy Micro Kit (Qiagen) according to the manufacturer's instructions. NanoDrop was used to determine RNA concentration and purity. cDNA was generated from 1 μg of total RNA by reverse transcription with oligo-d T primers (Invitrogen Superscript Kit) in a 20 µl reaction. For quantitative real-time RT-PCR, the cDNA was diluted 1:10, and a total of 5 µl cDNA was used in a 25 µl PCR reaction with gene-specific primers (2.5 μM) and the Power SYBR® Green PCR Master Mix (Life Technologies). mRNA relative expression was normalized against *Gapdh* mRNA using the ΔΔC*t* method. Primer sequences are listed in the following table.

For miRNA qRT-PCR, total RNA was reverse-transcribed using specific TaqMan miRNA RT primers and amplified using miRNA-specific TaqMan® assays (Applied Biosystems). The amount of miRNA was normalized to snoRNA202. Data analysis was performed using the ΔΔC*t* method. Reactions were run on a StepOnePlus™ machine (Applied Biosystems) with three biological replicates for each of the qRT-PCR experiments.

Primers used for mRNA quantification using qRT-PCR.

Primer	Primer sequence: (5' to 3')
Oct4-F	GAAGCCGACAACAATGAGAAC
Oct4-R	ATCCTTCTCTAGCCCAAGCTG
Sox2-F	AGAACCCCAAGATGCACAAC
Sox2-R	CTCCGGGAAGCGTGTACTTA
Esrrb-F	GGCGTTCTTCAAGAGAACCA
Esrrb-R	CCCACTTTGAGGCATTTCAT
Nanog-F	CTGATTCTTCTACCAGTCCCA
Nanog-R	AAACCAGGTCTTAACCTGCTTAT
Klf5-F	CCGGAGACGATCTGAAACAC
Klf5-R	CAGATACTTCTCCATTTCACATCTTG
Gata4-F	GGTTCCCAGGCCTCTTGCAATGCGG
Gata4-R	AGTGGCATTGCTGGAGTTACCGCTG
T (Brachyury)-F	CGAGATGATTGTGACCAAGAAC
T (Brachyury)-R	GGCCTGACACATTTACCTTCA
αMHC-F	TGGTCACCAACAACCCATACGACT
αMHC-R	TGTCAGCTTGTAGACACCAGCCTT
Pax6-F	TGAATGGGCGGAGTTATGAT
Pax6-R	GGACGGGAACTGACACTC
Nestin-F	CACACCTCAAGATGTCCC
Nestin-R	GAAAGCCAAGAGAAGCCT
Map2-F	CTAAAGAACATCCGTCACAG
Map2-R	CTTCACATTACCACCTCCA
Tubb3-F	GCCTCCTCTCACAAGTATG
Tubb3-R	CCTCCGTATAGTGCCCTT
FoxA2-F	GCAGACACTTCCTACTACCAA
FoxA2-R	CTCCACTCAGCCTCTCATTT
Sox7-F	AAGGATGAGAGGAAACGTCTG
Sox7-R	ATCCACATAGGGTCTCTTCTG
Sox17-F	GATGTAAAGGTGAAAGGCGA
Sox17-R	AAGACTTGCCTAGCATCTTG
Gapdh-F	GACTTCAACAGCAACTCCCAC
Gapdh-R	TCCACCACCCTGTTGCTGTA
Nkx2.5-F	CCTCGGGCGGATAAAAAAGAGC
Nkx2.5-R	TAGCGACGGTTCTGGAACCA
CK18-F	ATTTCAGTCTCAACGATGCC
CK18-R	GAACTCTGGTGTCATTAGTCTC
Rex1-F	TAGCCGCCTAGATTTCCACT
Rex1-R	GTCCATTTCTCTAATGCCCAC
Fgf5-F	CAAGTGCCAAATTTACGGATGA

Fgf5-R	GAACAGTGACGGTGAAGGAA
Map4-F	GGAGTGGCTTTCTTCCCAAGCTG
Map4-R	GGCCCTAAGATCAACATCAAGGAGG
Rbm26-F	GATGCAGGCTGGAGAAGAAGTCAC
Rbm26-R	CCGTGAGCTGTACCTCGACCTC
Idh2-F	CCGCCATTACCGAGAACACCAG
Idh2-R	AGTGCTCGTTCAGCTTCACATTGC
Srsf1-F	ACCCATACTGACAGTTGATACCAC
Srsf1-R	CTGGGAGGAAGAGAGGAACATTGC
Pura-F	GGCACCTCCTTGACTGTGGAC
Pura-R	AGAAGGTGTGTCCGAACTTGG
Myl12b-F	GAAGCCACAGGCACCATCCAG
Myl12b-R	GCCTCTCTGTACAGCTCATCCAC
Cbx3-F	AGATGCTGCTGACAAACCAAGG
Cbx3-R	AAGGCAATGACAATCTGAGGACAC
Pcbp2-F	TCGGCAGGTTACCATCACTGGATC
Pcbp2-R	TGGATGGGTCTGCTCTGTTCTAGC
Srsf11-F	GCCTGCAGAATCGTCACCAGTACC
Srsf11-R	GCCACCCAACACTAGCCATCTACC
P4ha1-F	TGAGCGATGTGTCTGCTGGAG
P4ha1-R	CACGGCCTTCGAAATTCTTGTCC
Prkag1-F	CCGAGTCTCCGCCTTACCTGTAG
Prkag1-R	CCACCAGACGGTGAACCTCTGC
Acat1-F	CACGGAGGAGCTGTTTCTCTGG
Acat1-R	CAGCCGGTCACATGGAACTGTC
Cpt1a-F	AGCCATGGAGGTTGTCCACGAG
Cpt1a-R	GTCCATCATGGCTTGTCTCAAGTG
Myo1b-F	CCGAGGTCCCCCTGGTAGATG
Myo1b-R	GAGAGTCGTGCGATACAGCTTGG
Ero1l-F	AGCGGACCAAGTTATGAGTTCCAG
Ero1l-R	GCACATTCCAACCGTCCTCCTCAG
Mrpl15-F	GAGGGCAGACTCCATTTTACATACG
Mrpl15-R	GGCAGCAATGGCTAATTCTGAAGC
Sbno1-F	CCTCTAGCATCTTCAGTCAGTCAG
Sbno1-R	CCAGGCTATGAGCTACTGTCTACC
Cdv3-F	CCTGTCAGTTACCTACCTTGTCAC
Cdv3-R	CTCCACCTCCTGCTTCCTACACTG

Cell cycle analysis

ESCs (initially seeded at 2.0×10^5 cells/well in 6-well plates) were trypsinized and harvested on day 3 post-transfection, then washed once at RT in 1X PBS. While vortexing, ice-cold 70% ethanol was added to the cells drop by drop in order to fix the cells. Next, the cells were incubated at -20˚C for at least 2 h, then washed twice with ice-cold PBS in order to remove the ethanol. For propidium iodide (PI)/RNase staining, the cells were resuspended in PI/RNase Staining Buffer (100 µg/ml RNase and 12.5 µg/ml PI) and incubated at RT (or 37˚C) for 15-30 min in the dark. Flow cytometry was performed using a BD LSR II flow cytometer (BD Biosciences) and the data were analyzed with BD FACSDiva (BD Biosciences). The experiments were performed in triplicate.

Alkaline phosphatase (AP) staining and colony formation assay

AP staining was performed using a Leukocyte Alkaline Phosphatase Kit (Sigma-Aldrich) according to the vendor's instructions. Briefly, cells were washed with PBS, fixed with 4% paraformaldehyde, washed with deionized water, stained for 15 min at RT in the dark, washed again with PBS, and then suspended in PBS for storage.

miRNA mimics were transfected into ESCs 24 h after plating (6.0×10^4 cells/well of 12-well plates) and 3 days later the cells were trypsinized and replated at 5.0×10^3 cells/well on gelatin-coated 24-well plates. AP activity was determined 5 days after replating. Differentiated (AP-negative) and undifferentiated (AP-positive) colonies were counted to determine clonogenicity.

Analysis of DNA methylation

To analyze the high-throughput methylation data of serum and 2i cells, the pre-processed whole-genome bisulfite sequencing data of the mESCs cultured in 2i and serum conditions were

obtained from GSE56879 (Angermueller et al., 2016). From all CpGs, we kept only those with minimum sequencing coverage of 5 reads in both 2i and serum conditions. Methylation ratio of each condition and the difference between conditions were computed using custom R script. The results were visualized using IGV version 2.3.67 (Robinson et al., 2011).

To analyze DNA methylation using methylation-sensitive restriction enzyme digestion followed by qPCR (MSRE-qPCR), the methylation level of a selected set of CpG dinucleotides within the three DMRs of the *Dlk1-Dio3* locus was determined by restriction enzyme digestion and real-time PCR analysis using specific primers designed to amplify genomic regions flanking specific restriction sites within the *Dlk1-Dio3* locus. Two methylation-sensitive enzymes HhaI and HpaII (NEB, UK) were used to digest the DNA. Preparation of DNA templates, enzymatic digestion and real-time PCR analysis were performed according to Oakes and colleagues (Oakes et al., 2006). Briefly, DNA was extracted from 2i, R2i and serum cells using DNeasy Blood & Tissue Kit (Qiagen, USA). 2µg DNA (50 ng/µl) was digested by HhaI or HpaII. A mock digestion with no enzyme (sham) was performed for each sample. In order to perform qPCR, the *Dlk1-Dio3* region (GeneBank ID: AJ320506.1) was used as template and specific primer pairs were designed to amplify the flanking fragments of either HhaI or HpaII restriction sites (see Table below). A control primer pair (F: GGTGCCAGCAGAGACTTACACAG and R: CATGCCCTTTGACACTTAGTATGC) was designed for *β2m* gene to amplify a region that is devoid of the restriction sites of HpaII and HhaI. Real-time PCR was performed using a StepOnePlus instrument (Thermofisher, USA) in a final reaction volume of 20 µl with 12.5 ng of digested template, 10 µl SYBR green master mix 2X and 200 nM of each forward and reverse primers. The thermal reaction condition was as follows: an initial denaturation at 95 °C for 10

min, followed by 40 cycles of denaturation at 95 °C for 10 s, annealing at 60 °C for 20 s and extension at 72 °C for 20 s. All reactions were run in triplicate.

Primers used for DNA methylation analysis.

**DMR**	**CpG Position in DMR (AJ320506.1)**	**Primer pair**	**Enzyme**
	12062	F: 5'-AGCTGCAATGCTCATTCCTAGTG-3'<br>R: 5'-TAGTGGTCTATTCACCCAAGTGC-3'	HpaII
	12678	F: 5'-GGAAAGGGCATGGGAGAGGAC-3'<br>R: 5'-CCATCGTTCTCGCATGGGTTAGG-3'	HhaI
	13270	F: 5'-AGGCCATCTGCTTCACCATCC-3'<br>R: 5'-CGCTGTTATACTGCAACAGGAG-3'	HhaI
	14538	F: 5'-GCCCAAGACTCCACCTCATGC-3'<br>R: 5'-CACCCCACAAGCCATAGTGTC-3'	HpaII
	79652	F: 5'- GGATCCTGACCTATGTGTACCTCTG-3'<br>R: 5'- ACGGACCGTGTGTATGTGCTGTAG-3'	HhaI
	79723	F: 5'- GGATCCTGACCTATGTGTACCTCTG-3'<br>R: 5'- ACGGACCGTGTGTATGTGCTGTAG-3'	HpaII
	80437	F: 5'-TCTTGTGGCAAAGGTACGTGACTG-3'<br>R: 5'-GTATGCTATGCATTTGTGCTGAAGG-3'	HpaII
	81915	F: 5'-AGCTGACTTCCTTCAGCCACAGT-3'<br>R: 5'-TTGACCCTGTGAGAGATGCTCAG-3'	HpaII
	81915	F: 5'-AGCTGACTTCCTTCAGCCACAGT-3'<br>R: 5'-TTGACCCTGTGAGAGATGCTCAG-3'	HpaII
	85733	F: 5'- AGTTTCTGGGAAACGTACAGAAGG-3'<br>R: 5'- CACTTCTCTGCAAGGCCAAGTC-3'	HhaI
	95372	F: 5'-GGTCGGGAGCGAGATGGGTTG-3'<br>R: 5'-GCGTCCATGACACCCTAAATCAC-3'	HhaI
	97768	F: 5'-TCCCGTTCATGGCTCATGTGTCTC-3'<br>F: 5'-GCCCTGGAAATGACCGCACACTC-3'	HhaI
	97800	F: 5'-TCCCGTTCATGGCTCATGTGTCTC-3'<br>F: 5'-GCCCTGGAAATGACCGCACACTC-3'	HpaII
	99823	F: 5'-CCCTCTCAGTTTCCCAAACCTG-3'<br>R: 5'-CCAAGGTATCCTGGAAGAGCTGAC-3'	HpaII

Small RNA deep sequencing

Total RNA was extracted from the mESC samples and used to generate small RNA libraries from which cDNA libraries were prepared at LC Sciences. The purified cDNA libraries were

then used for cluster generation on Illumina's Cluster Station and then sequenced on Illumina GAIIx (Illumina HiSeq 1000/Illumina GAIIx) according to the vendor's instruction for running the instrument. Raw sequencing reads (up to 45 nt) were obtained using Illumina's Sequencing Control Studio software version 2.8 following real-time sequencing image analysis and base-calling by Illumina's Real-Time Analysis version 1.8.70 (LC Sciences).

Bioinformatics and statistical analyses

In order to determine the expression levels of miRNAs across the samples, we used miARma-Seq pipeline for raw data analysis. Briefly, the pre-processed sequences were subjected to quality assessment using FastQC v0.11.5. The minimum observed Phred score across all samples and all bases was 30 (Sanger/Illumina 1.9 encoding), which depicted the high quality of sequencing procedure. Cutadapt v1.3 (Creighton et al., 2008) was used to trim the adapter sequences (TruSeq small RNA 3' adapter TGGAATTCTCGGGTGCCAAGG and small RNA sequencing 5' primer TTCAGAGTTCTACAGTCCGACGATC according to Illumina Adapter Sequences manual v01, Feb 2016) and remove the processed sequences shorter than 15 or longer than 35 bases. A second quality control was performed after the adapter trimming using FastQC. We then used bowtie v1.1.2 (Langmead et al., 2009) to align the processed reads against *Mus musculus* genome assembly mm10 (GRCm38) obtained from the UCSC Genome Bioinformatics. The location of the miRNAs and other small non-coding RNAs was processed from the miRBase genome coordinates mmu.gff3 v21. The number of reads that were mapped to the genomic location of each mature miRNA was counted using the featureCounts v1.5.0-p1 (Liao et al., 2014).

Cross-sample normalization and differential expression analysis were performed based on a Negative Binomial distribution analysis by R/Bioconductor package DESeq2 (Love et al., 2014). The miRNAs with the absolute log2-fold change greater than 1 and the adjusted *p*-values less than 0.01 (Bonferroni-Hochberg) were considered significantly differentially expressed. Principal Components Analysis (PCA), pairwise Pearson correlation coefficients heatmaps, and visualization of the results was performed by a custom R script using several packages such as ggplot2, pheatmap, plyr, reshape2, and gplots. Target prediction analysis was performed by miRGate database (Andres-Leon et al., 2015). The predicted targets that were reported in the TargetScan database (Lewis et al., 2005) were considered for further analysis. The gene ontology (GO) analysis was done by the DAVID gene annotation tool v6.8 (Huang da et al., 2009) and Enrichr (Kuleshov et al., 2016). We also used GraphPad Prism v5.0 to visualize some of the results.

For all assays, n represents the number of independent experiments.

III. Supplemental References

Andres-Leon, E., Gonzalez Pena, D., Gomez-Lopez, G., and Pisano, D.G. (2015). miRGate: a curated database of human, mouse and rat miRNA-mRNA targets. Database (Oxford) bav035.

Angermueller, C., Clark, S.J., Lee, H.J., Macaulay, I.C., Teng, M.J., Hu, T.X., Krueger, F., Smallwood, S.A., Ponting, C.P., Voet, T., *et al.* (2016). Parallel single-cell sequencing links transcriptional and epigenetic heterogeneity. Nat. Methods *13*, 229-232.

Creighton, C.J., Nagaraja, A.K., Hanash, S.M., Matzuk, M.M., and Gunaratne, P.H. (2008). A bioinformatics tool for linking gene expression profiling results with public databases of microRNA target predictions. RNA *14*, 2290-2296.

Huang da, W., Sherman, B.T., and Lempicki, R.A. (2009). Systematic and integrative analysis of large gene lists using DAVID bioinformatics resources. Nat. Protocols *4*, 44-57.

Kuleshov, M.V., Jones, M.R., Rouillard, A.D., Fernandez, N.F., Duan, Q., Wang, Z., Koplev, S., Jenkins, S.L., Jagodnik, K.M., Lachmann, A., *et al.* (2016). Enrichr: a comprehensive gene set enrichment analysis web server 2016 update. Nucleic Acids Res. *44*, W90-97.

Langmead, B., Trapnell, C., Pop, M., and Salzberg, S.L. (2009). Ultrafast and memory-efficient alignment of short DNA sequences to the human genome. Genome Biol. *10*, R25.

Lewis, B.P., Burge, C.B., and Bartel, D.P. (2005). Conserved seed pairing, often flanked by adenosines, indicates that thousands of human genes are microRNA targets. Cell *120*, 15-20.

Liao, Y., Smyth, G.K., and Shi, W. (2014). featureCounts: an efficient general purpose program for assigning sequence reads to genomic features. Bioinformatics *30*, 923-930.

Love, M.I., Huber, W., and Anders, S. (2014). Moderated estimation of fold change and dispersion for RNA-seq data with DESeq2. Genome Biol. *15*, 550.

Oakes, C.C., La Salle, S., Robaire, B., and Trasler, J.M. (2006). Evaluation of a quantitative DNA methylation analysis technique using methylation-sensitive/dependent restriction enzymes and real-time PCR. Epigenetics *1*, 146-152.

Robinson, J.T., Thorvaldsdottir, H., Winckler, W., Guttman, M., Lander, E.S., Getz, G., and Mesirov, J.P. (2011). Integrative genomics viewer. Nat. Biotechnol. *29,* 24-26.
