# Supplementary material for: Small RNA Sequencing Reveals Dlk1-Dio3 Locus-Embedded MicroRNAs as Major Drivers of Ground-State Pluripotency
Source: Stem Cell Reports. 2017 Nov 9;9(6):2081–96. doi: 10.1016/j.stemcr.2017.10.009 (PMC5785679; doi:10.1016/j.stemcr.2017.10.009)
Supplement: Document S2. Article plus Supplemental Information [file mmc3.pdf]

Stem Cell Reports
Resource
OPEN ACCESS

Small RNA Sequencing Reveals *Dlk1-Dio3* Locus-Embedded MicroRNAs as Major Drivers of Ground-State Pluripotency

Sharif Moradi,[1,2] Ali Sharifi-Zarchi,[1,3] Amirhossein Ahmadi,[1] Sepideh Mollamohammadi,[1] Alexander Stubenvoll,[4] Stefan Günther,[4] Ghasem Hosseini Salekdeh,[1] Sassan Asgari,[5] Thomas Braun,[4,*] and Hossein Baharvand[1,2,*]
[1]Department of Stem Cells and Developmental Biology, Cell Science Research Center, Royan Institute for Stem Cell Biology and Technology, ACECR, Banihashem Square, Banihashem Street, Ressalat Highway, Tehran 1665659911, Iran
[2]Department of Developmental Biology, University of Science and Culture, Tehran, Iran
[3]Computer Engineering Department, Sharif University of Technology, Tehran, Iran
[4]Max-Planck Institute for Heart and Lung Research, Department of Cardiac Development and Remodelling, Ludwigstrasse 43, 61231 Bad Nauheim, Germany
[5]Australian Infectious Disease Research Centre, School of Biological Sciences, The University of Queensland, Brisbane, QLD, Australia
*Correspondence: thomas.braun@mpi-bn.mpg.de (T.B.), baharvand@royaninstitute.org (H.B.)
https://doi.org/10.1016/j.stemcr.2017.10.009

SUMMARY

Ground-state pluripotency is a cell state in which pluripotency is established and maintained through efficient repression of endogenous differentiation pathways. Self-renewal and pluripotency of embryonic stem cells (ESCs) are influenced by ESC-associated microRNAs (miRNAs). Here, we provide a comprehensive assessment of the “miRNome” of ESCs cultured under conditions favoring ground-state pluripotency. We found that ground-state ESCs express a distinct set of miRNAs compared with ESCs grown in serum. Interestingly, most “ground-state miRNAs” are encoded by an imprinted region on chromosome 12 within the *Dlk1-Dio3* locus. Functional analysis revealed that ground-state miRNAs embedded in the *Dlk1-Dio3* locus (miR-541-5p, miR-410-3p, and miR-381-3p) promoted pluripotency via inhibition of multi-lineage differentiation and stimulation of self-renewal. Overall, our results demonstrate that ground-state pluripotency is associated with a unique miRNA signature, which supports ground-state self-renewal by suppressing differentiation.

INTRODUCTION

Embryonic stem cells (ESCs) are derived from the inner cell mass of blastocyst-stage embryo and provide a perpetual cell source to investigate pluripotency and stem cell self-renewal *in vitro* (Evans and Kaufman, 1981; Hassani et al., 2014a; Martin, 1981). ESCs were originally derived and maintained in serum-containing media on feeder cells (Evans and Kaufman, 1981; Martin, 1981). Further studies revealed that feeder cells provide leukemia inhibitory factor (LIF) whereas serum provides bone morphogenetic protein (BMP) signals, which inhibit ESC differentiation into mesendoderm and neuroectoderm, respectively (Ying et al., 2003). Based on these findings, ESC cultures supplemented with BMP and LIF signals have been used to maintain ESCs in an undifferentiated state and to suppress endogenous differentiation-promoting signals (Ying et al., 2003). Notably, pharmacological inhibition of endogenous pro-differentiation ESC signals allows maintenance and establishment of ESCs from different mouse and rat strains. Such culture conditions are defined as 2i, whereby two small-molecule inhibitors are used to block the glycogen synthase kinase 3 (GSK3) and fibroblast growth factor-extracellular regulated kinase (FGF-ERK) pathways, allowing indefinite growth of ESCs without the need for exogenous signals. This so-called ground state of pluripotency displays robust pluripotency due to efficient repression of intrinsic differentiation signals and shows a remarkable homogeneity compared with ESCs kept in serum (Wray et al., 2010; Ying et al., 2008).

Recently, we devised alternative culture conditions, dubbed R2i, which allow ground-state cultivation and efficient generation of ESCs from pre-implantation embryos (Hassani et al., 2014b). R2i conditions feature inhibition of transforming growth factor β (TGF-β) and FGF-ERK signaling instead of GSK3 and FGF-ERK blockage used in the 2i approach. Compared with GSK3 inhibition, suppression of TGF-β signaling reduces genomic instability of ESCs and allows derivation of ESCs from single blastomeres at much higher efficiency (Hassani et al., 2014a, 2014b). Since 2i and R2i ESCs both represent the ground state of ESC pluripotency, a systematic comparison of similarities and differences might aid in the understanding of core mechanisms underlying ground-state pluripotency.

MicroRNAs (miRNAs) are ∼22-nt long non-coding RNAs that post-transcriptionally regulate a large number of genes in mammalian cells, thereby modulating virtually all biological pathways including cell-fate decisions and reprogramming (Baek et al., 2008; Bartel, 2009; Moradi et al., 2014; Sayed and Abdellatif, 2011). In ESCs, ablation of miRNA-processing enzymes impairs self-renewal, rendering ESCs unable to differentiate (Kanellopoulou et al., 2005; Wang et al., 2007). Individual miRNAs play important roles in ESC regulation. miR-290–295 cluster or

This is an open access article under the CC BY license (http://creativecommons.org/licenses/by/4.0/).

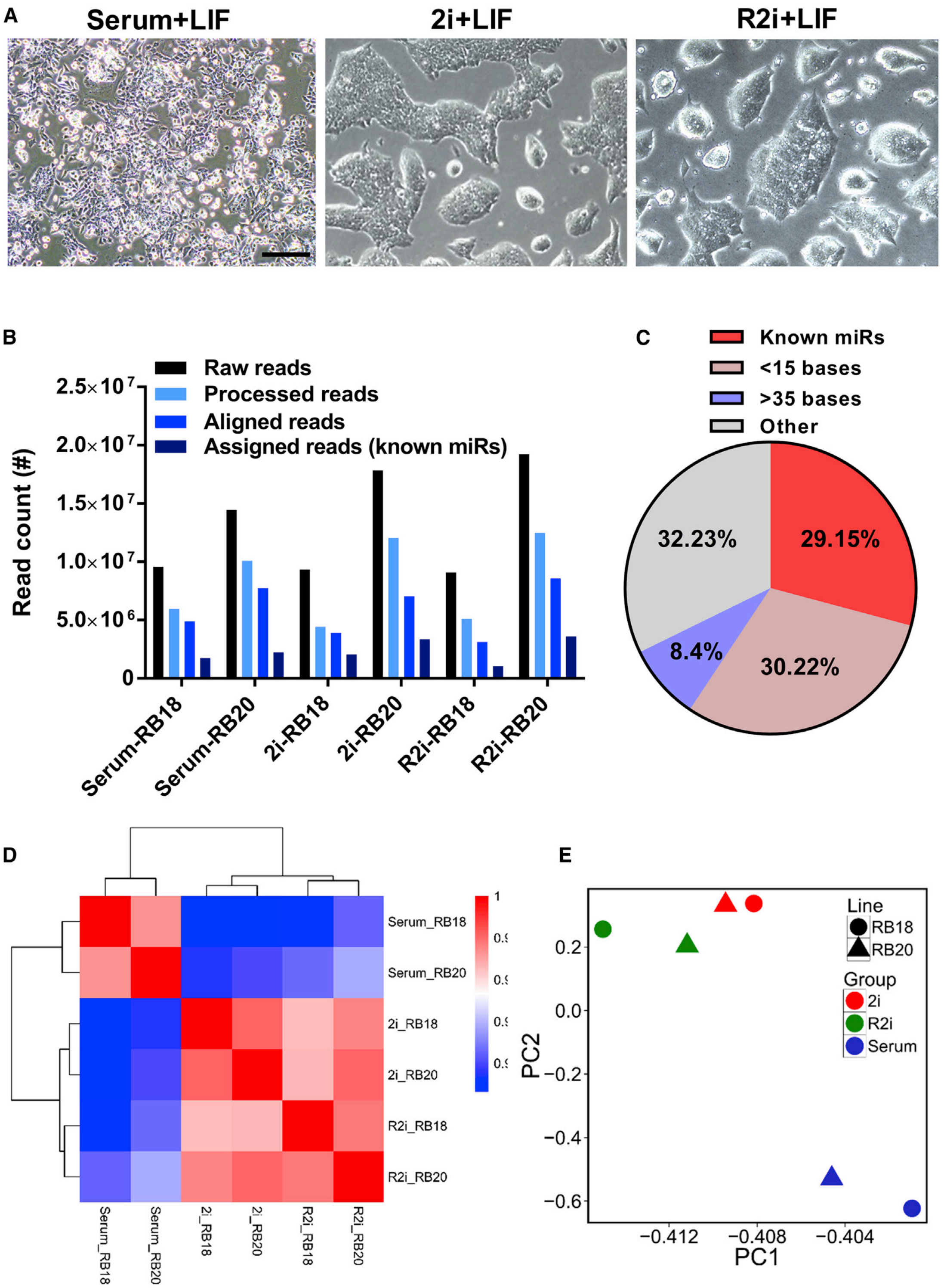

Figure 1. Small RNA Sequencing of ESCs Cultured under Serum, 2i, and R2i Conditions

(A) Phase-contrast images of ESCs cultured under serum, 2i, and R2i. Scale bar, 200 μm.

(B) Raw, processed, aligned, and assigned read counts of each ESC sample. Small RNA-sequencing data were obtained for two ESC lines (RB18 and EB20) each time using pools of three independently grown cultures (independent experiments).

(legend continued on next page)

let-7 family members, for example, promote or impair ESC self-renewal, respectively (Melton et al., 2010). Moreover, miRNAs enriched in ESCs promote de-differentiation of somatic cells into induced pluripotent stem cells (iPSCs) (Moradi et al., 2014). So far, most studies have focused on the expression and functional significance of miRNAs in ESCs kept in serum (Graham et al., 2016; Hadjimichael et al., 2016; Houbaviy et al., 2003; Liu et al., 2014; Marson et al., 2008; Melton et al., 2010; Parchem et al., 2015; Tay et al., 2008; Wang et al., 2008), which leaves a critical gap about the functional importance of miRNAs in ESCs cultured in ground-state conditions despite many insights into the transcriptome, epigenome, and proteome of ground-state pluripotency (Habibi et al., 2013; Marks et al., 2012; Taleahmad et al., 2015).

In the present study, we analyzed the global expression patterns of miRNAs in ESCs cultured in ground-state conditions of 2i and R2i compared with serum using small RNA sequencing. We provide a comprehensive report on the "miRNome" of ground-state pluripotency compared with serum cells, which enabled us to identify miRNAs specific to each cell state. Furthermore, we found that selected ground-state miRNAs contribute to the maintenance of ground-state pluripotency by promoting self-renewal and repressing differentiation.

RESULTS

Analysis of Small RNA Expression in Ground-State ESCs

To obtain a comprehensive expression profile of miRNAs in ground-state ESCs, we used the RB18 and RB20 ESC lines maintained under feeder-free conditions in serum, 2i, or R2i cultures. RB18 and RB20 ESC lines were initially derived from C57BL/6 mice using the R2i + LIF protocol (Hassani et al., 2014b). Isolated R2i cells were then transferred to 2i or serum-containing medium and passaged at least 10 times to derive stable 2i and serum cell lines (Figure 1A). Pluripotency of established cell lines was confirmed by chimera formation and germline contribution as previously reported (Hassani et al., 2014b). Small RNA-sequencing data were obtained for both the RB18 and the EB20 ESC lines each time using pools of three independently grown cultures.

Analysis of the small RNA profiles of ESC samples revealed a total of 79,520,099 raw reads. After removal of adaptors and reads with <15–35> bases, the processed reads were aligned to mouse genome, which yielded a total of 35,287,315 mappable reads (Figure 1B). We found that on average 29.15% of the processed reads were matched to known mouse miRNAs (Figure 1C). Analysis of the length distribution revealed two peaks (Figure S1A). The minor peak represented Piwi-interacting RNAs (piRNAs with a size range of 26–31 nt) (Yang et al., 2013), whereas the major peak represented mature miRNAs (~22 nt long) (Bartel, 2004).

A Pearson correlation coefficient heatmap (Figure 1D) revealed a remarkably high degree of correlation between miRNA profiles in serum, 2i, and the R2i cell lines, which was also supported by hierarchical co-clustering of the samples. The heatmap revealed that 2i and R2i cells showed greater similarity to each other compared with serum cells, which suggests that 2i and R2i cultures share a similar miRNA profile that might reflect the ESC ground state. Moreover, two-dimensional principal component analysis showed that 2i and R2i cells were located in close proximity to each other, but quite distinct from serum cells (Figure 1E), demonstrating that ground-state pluripotency features a unique signature of small RNA expression. Since both cell lines showed virtually identical results, we merged the data from both ESC lines for the subsequent analyses.

Expression Patterns of miRNAs Associated with Pluripotency and Differentiation

Bioinformatics analysis identified a set of 20 miRNAs, which were abundantly expressed under all conditions in ESCs (Figure S1B). Members of the miR-290–295 cluster represented the most highly expressed miRNAs, as previously reported (Marson et al., 2008). Interestingly, expression of most members of the miR-290–295 cluster did not change significantly in different culture conditions (Figure S2A). Likewise, we observed that several other pluripotency-associated miRNAs (e.g., miR-182-5p and miR-183-5p) were expressed at similarly high levels under all conditions (Figure S1C), suggesting that the most abundant miRNAs in ESCs did not undergo major expression changes under different culture conditions.

In contrast, members of the miR-302–367 cluster were mostly upregulated in 2i/R2i cells compared with serum ESCs (Figure S2B). This finding is in disagreement with results reported by Parchem et al. (2015), who suggested that miR-302b is expressed at higher levels in serum

(C) Pie chart indicating the proportion of processed reads accounting for miRNAs. Small RNA profiles were obtained for RB18 and EB20 ESC lines using pools of three independently grown cultures (independent experiments).
(D) Pearson heatmap of the ESC samples. Samples are shown as rows and columns. Each square represents the Pearson correlation coefficient between two samples. Each small RNA profile was obtained from three independently grown cultures (independent experiments).
(E) Principal component (PC) analysis of the ESC samples maintained under serum, 2i, and R2i culture conditions. Each small RNA-sequencing data was obtained from three independently grown cultures (independent experiments).

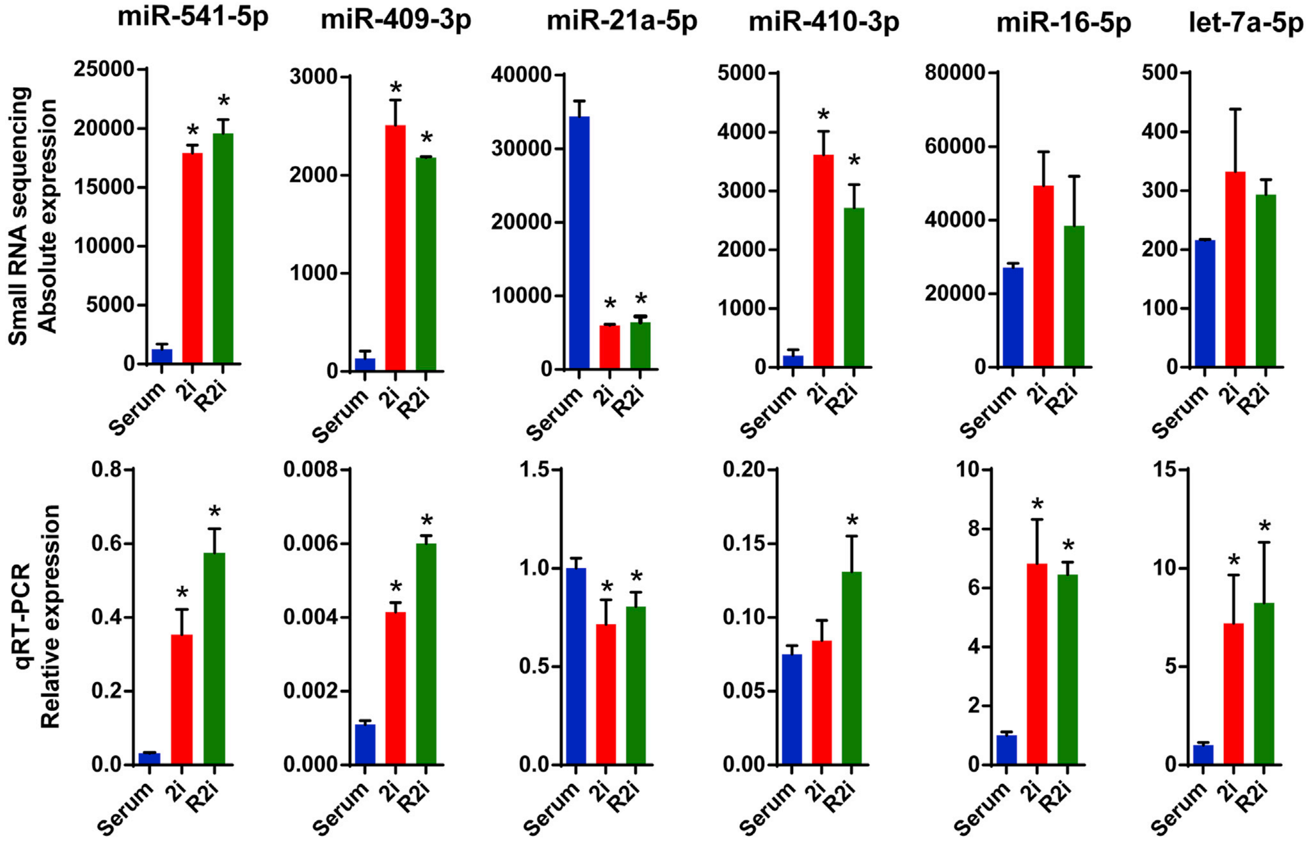

Figure 2. qRT-PCR Confirmation of the Small RNA-Sequencing Data

A selected set of miRNAs with differential expression in serum, 2i, and R2i conditions were chosen for qRT-PCR confirmation. Data are shown as mean ± SD, n = 3. *p < 0.05. Each small RNA-sequencing datum was obtained from three independently grown cultures (independent experiments).

compared with 2i cells. However, in the previous study 2i chemicals were added to serum-containing medium, whereas in the current study 2i (similar to R2i) cells were cultured serum free, which might explain the discrepancy. Analysis of the expression patterns of the pluripotency-associated miR-17 family (consisting of three clusters) revealed that most members of the miR-17–92 and miR-106b-25 clusters did not change their expression patterns in different ESC media (Figures S2C and S2D). In contrast, most members of the miR-106a~363 cluster were upregulated in serum ESCs compared with 2i and R2i (Figure S2E).

We next asked whether miRNAs associated with differentiation were differentially expressed (Figure S2F). We found that the majority of differentiation-affiliated miRNAs were upregulated in serum cells compared with 2i and R2i, suggesting that serum + LIF failed to completely suppress differentiation-associated processes. Interestingly, we found that a small number of other differentiation-associated miRNAs including let-7g-5p were more abundant in ground state compared with serum cells, although their read numbers were very small (data not shown). This finding is consistent with a previous study showing increased expression of let-7 miRNAs in 2i compared with serum (Kumar et al., 2014). We hypothesize that some differentiation-associated miRNAs might promote some features of ground-state pluripotency and/or render ground-state cells "poised" for rapid differentiation once 2i/R2i + LIF components of the ESC medium are removed from culture.

To validate the expression patterns of miRNAs obtained by small RNA sequencing, we performed TaqMan miRNA qRT-PCR assays of RNA isolated from serum, 2i, and R2i cells, and observed highly similar results compared with small RNA sequencing (Figure 2). However, we noted statistically significant differences in the expression of let-7a-5p and miR-16-5p between the two platforms. Nevertheless the differences were minor, indicating that the small RNA-sequencing data are suitable for further analysis.

Identification of Differentially Expressed miRNAs

Our small RNA-sequencing approach detected 1,233 miRNAs in each ESC state. Scatterplots indicated that

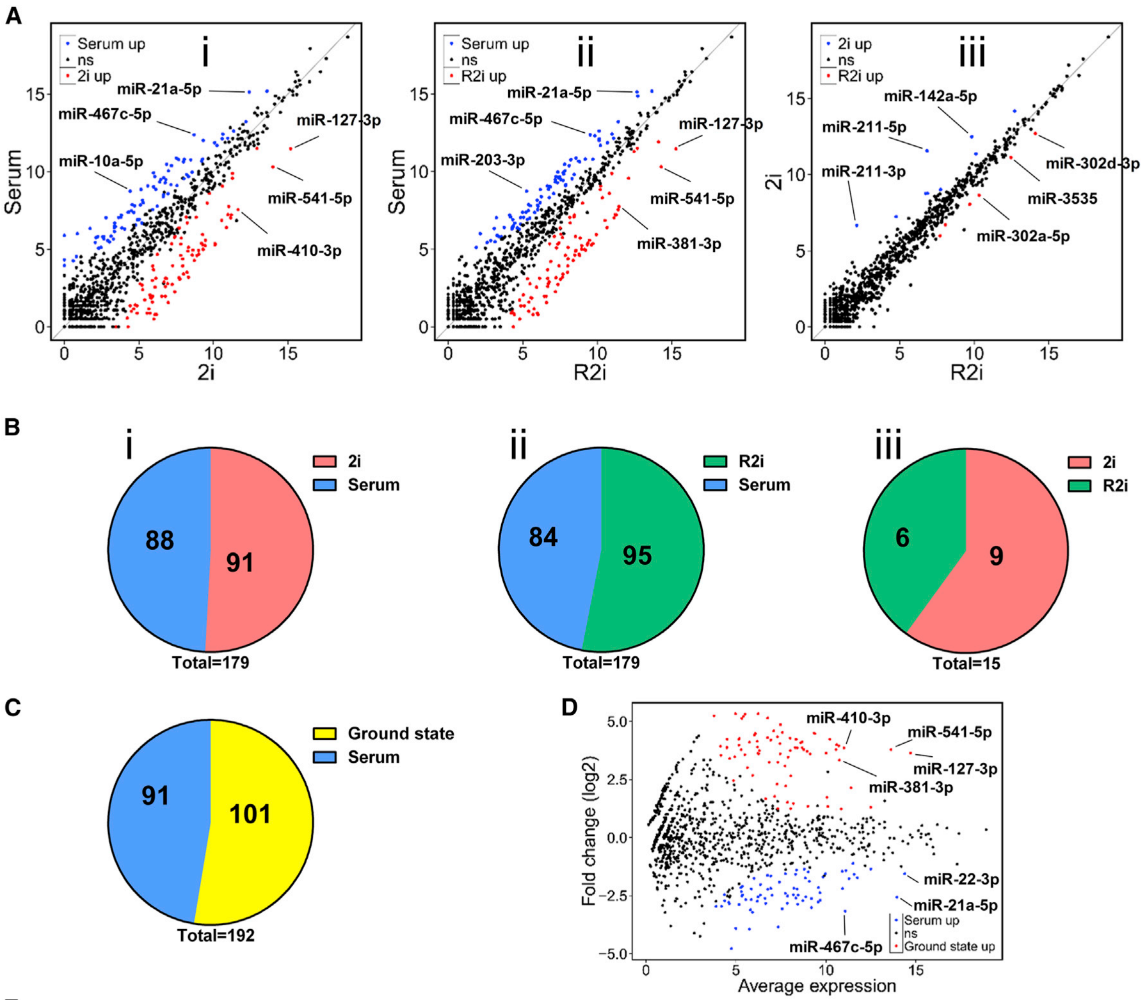

E

	# upregulated (p<0.01)	# predicted mRNA targets*	# predicted unique mRNA targets**
Serum ESCs	91	878	649
Ground state ESCs	101	1241	1012

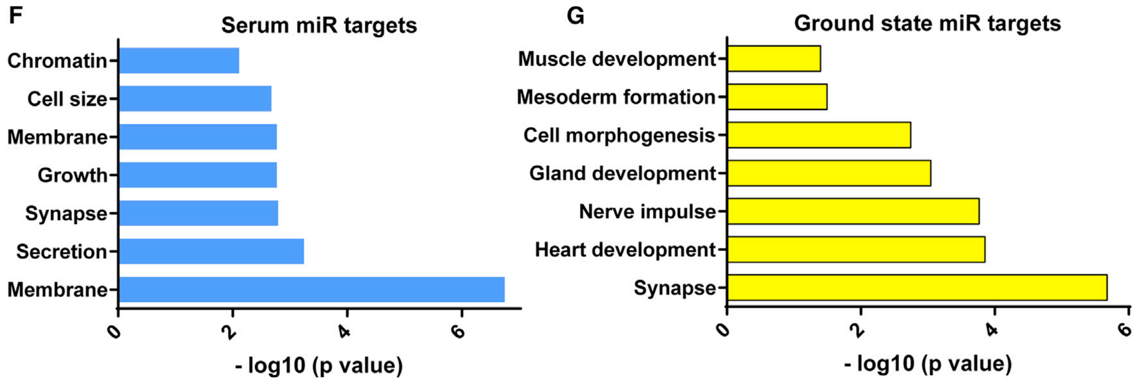

(legend on next page)

miRNAs were significantly differentially expressed under different conditions (Figures 3Ai–iii). Some miRNAs such as miR-127-3p and miR-410-3p were upregulated in 2i versus serum, whereas miR-467c-5p and miR-10a-5p were upregulated in serum (Figure 3Ai). We observed that miR-21a-5p and miR-203-3p were expressed significantly higher in serum than in R2i, whereas miR-381-3p and miR-127-3p were expressed much higher in R2i than in serum (Figure 3Aii). Moreover, although the miRNome of 2i and R2i states was strikingly similar, some miRNAs such as miR-211-5p and miR-142a-5p were differentially expressed between 2i and R2i (Figure 3Aiii). These data show that ESCs' miRNA expression changes dynamically in response to changes in extrinsic signals.

A global assessment of miRNAs exhibiting >2-fold differences in expression revealed that 179 miRNAs were differentially expressed between serum and 2i cells (Figure 3Bi; Tables S1 and S2). Additionally, when comparing R2i with serum cells, we found that 95 miRNAs were upregulated in R2i and 84 miRNAs were upregulated in serum (Figure 3Bii; Tables S3 and S4). In addition, 2i cells expressed nine miRNAs more abundantly than R2i cells, whereas R2i cells expressed six miRNAs more abundantly than 2i cells (Figure 3Biii and Table S5).

Next, we sought to pinpoint miRNAs associated with serum or ground-state pluripotency. We defined ground-state-associated miRNAs as miRNAs that are significantly upregulated in both 2i and R2i. Our data identified 91 miRNAs associated with serum and 101 miRNAs associated with the ground state (Figure 3C and Table S6). Top serum-enriched miRNAs included miR-21a-5p and miR-467c-5p, while top miRNAs upregulated in ground state included miR-381-3p and miR-541-5p (Figure 3D). To identify the biological pathways that are potentially modulated by serum and ground-state miRNAs in ESCs, we performed miRNA target prediction using TargetScan for the miRNAs enriched in each ESC state (Figure 3E). A total of 878 transcripts were predicted to be targeted by miRNAs under serum conditions and 1,241 transcripts were potentially targeted by miRNAs specific to ground state. To identify only pathways that are "specifically" regulated by state-specific miRNAs, we excluded all transcripts co-targeted by both sets of miRNAs. This rationale reduced the number of predicted targets to 649 (serum) and 1,012 (ground state) (Table S7). DAVID analysis revealed that miRNAs associated with the serum condition did not modulate critical pathways associated with fate decision in ESCs (Figure 3F), while miRNAs associated with ground-state pluripotency were predicted to control key developmental processes predominantly related to differentiation (Figure 3G). Our gene ontology (GO) analysis using Enrichr also indicated that in contrast to serum-up miRNAs, which did not seem to modulate ESC fate decisions, ground-state-up miRNAs targeted several crucial differentiation-associated pathways including neurogenesis and organ morphogenesis (Figure S3). We reasoned that miRNAs upregulated in ground-state cells might contribute to the inhibition of differentiation, which is in line with the concept that ground-state pluripotency is achieved by repression of differentiation processes (Wray et al., 2010; Ying et al., 2008).

Chromosomes Differentially Contribute to the Production of Mature miRNAs in ESCs

Next, we sought to profile the global chromosomal distribution of miRNAs detected in the different ESC samples. miRNAs were mapped to different chromosomes and the relative expression of mature miRNAs per chromosome was determined. miRNAs were encoded on and expressed in ESCs by all chromosomes other than the Y chromosome (Figures 4A and S4A). Four chromosomes (6, 7, 14, and X) were observed to transcribe miRNAs more actively than other chromosomes (Figure S4A) and, interestingly, these four chromosomes expressed the most abundant miRNAs in ESCs: chromosome 6 harbors miR-182, miR-183, and miR-148a species; chromosome 7 harbors the miR-290–295 cluster; chromosome 14 harbors the miR-17–92 cluster and miR-16; and chromosome X harbors the miR-106a~363 cluster. These observations indicate that chromosomes 6, 7, 14, and X code for and express the most abundant miRNAs in ESCs (see Figures S1B and S1C).

Figure 3. Differentially Expressed miRNAs

(A) Scatterplots indicating the global expression pattern of miRNAs in (i) 2i versus serum, (ii) R2i versus serum, and (iii) 2i versus R2i. Each small RNA-sequencing datum was obtained from three independently grown cultures (independent experiments).

(B) Pie charts indicating the number of differentially expressed miRNAs for (I) 2i versus serum, (ii) R2i versus serum, and (iii) 2i versus R2i. Each small RNA-sequencing datum was obtained from three independently grown cultures (independent experiments).

(C) Pie chart showing the number of differentially expressed miRNAs for ground-state ESCs versus serum. Each small RNA-sequencing datum was obtained from three independently grown cultures (independent experiments).

(D) Scatterplot indicating the global expression pattern of miRNAs in ground-state ESCs versus serum. Each small RNA-sequencing datum was obtained from three independently grown cultures (independent experiments).

(E) miRNAs upregulated in serum and ground-state cells plus the number of transcripts predicted to be targeted by these miRNAs before and after the exclusion of commonly targeted mRNAs.

(F and G) GO analysis of transcripts predicted to be targeted by ground-state (F) and serum-associated miRNAs (G).

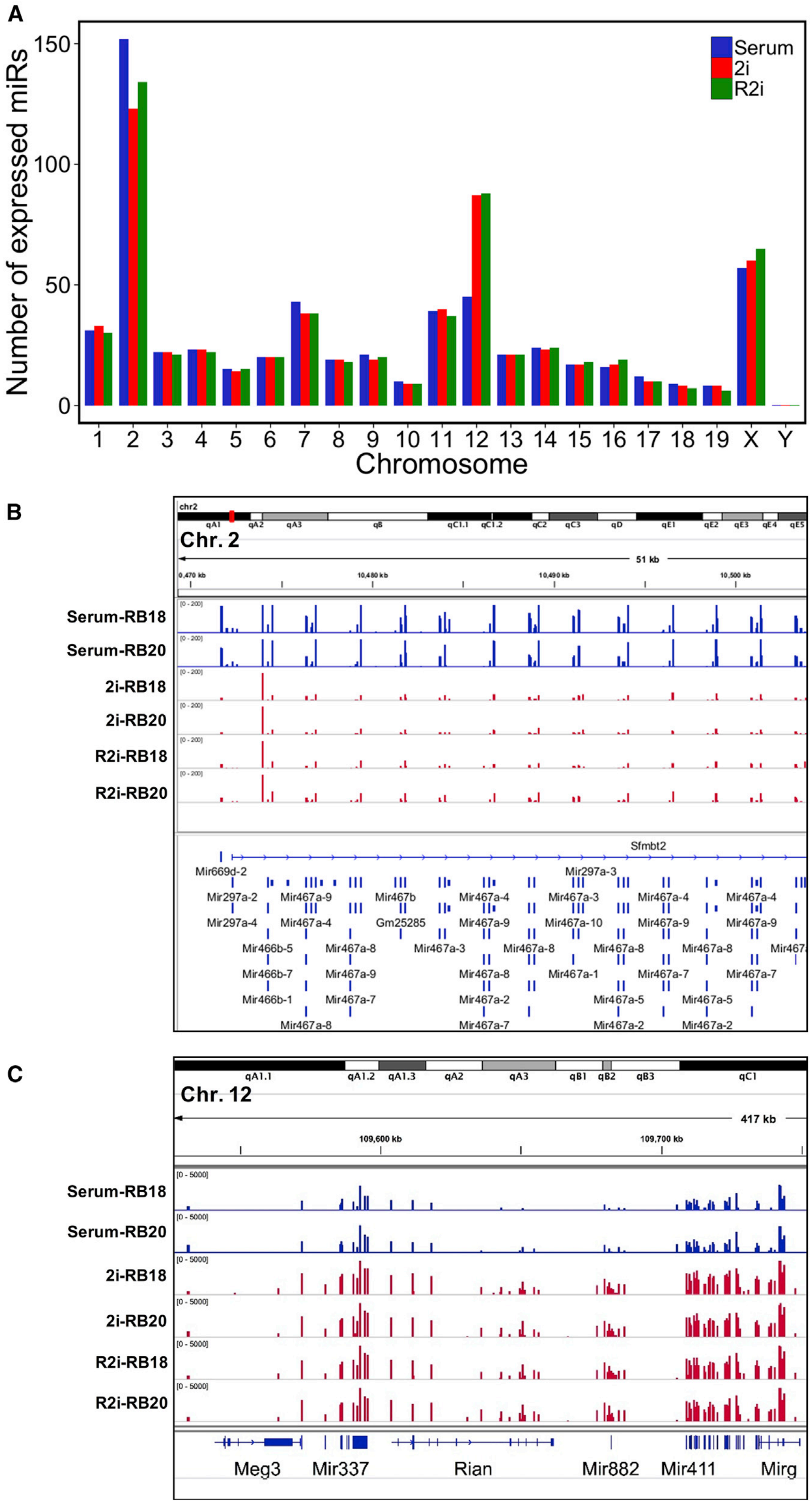

Figure 4. Contributions of Chromosomes to miRNA Generation in ESCs

(A) Chromosome pattern of miRNA expression in serum, 2i, and R2i cultures. The y axis indicates the number of miRNA-coding sequences within chromosomes which are transcribed as mature miRNAs. Each small RNA-sequencing datum was obtained from three independently grown cultures (independent experiments).

(B) Integrative Genomics Viewer (IGV) browser screenshot indicating miRNA read alignments in the 10th intron of *Sfmbt2* locus (chromosome 2). Small RNA-sequencing data were obtained for two ESC lines (RB18 and EB20) each time using pools of three independently grown cultures (independent experiments).

(C) IGV visualization of the read alignments of miRNAs embedded within the *Dlk1-Dio3* locus (chromosome 12). Small RNA-sequencing data were obtained for two ESC lines (RB18 and EB20) each time using pools of three independently grown cultures (independent experiments).

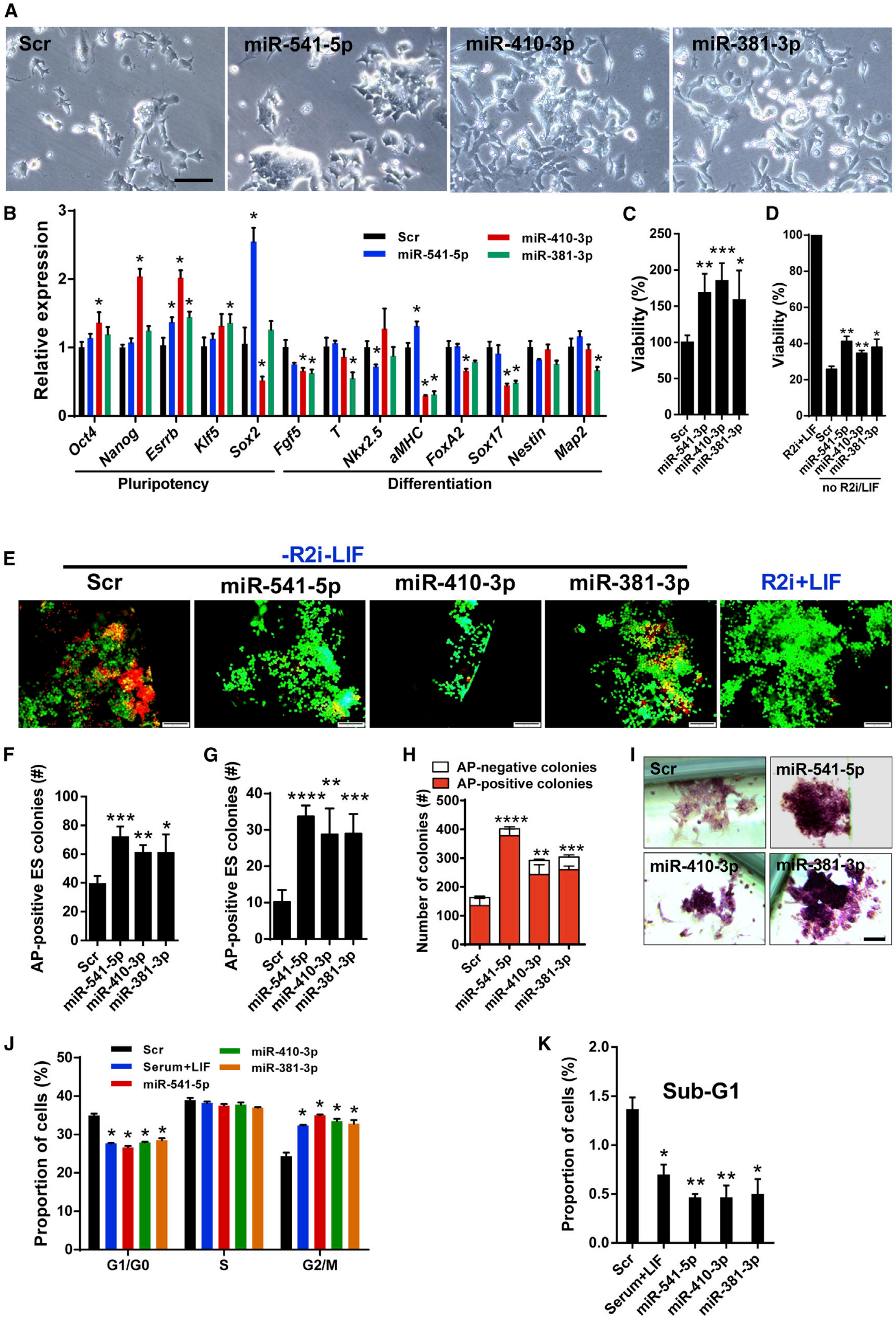

(legend on next page)

Next, we determined how many miRNA genes are active per chromosome in different ESCs. As shown in Figure 4A, two chromosomes (2 and X) in serum ESCs and three chromosomes (2, 12, and X) in 2i/R2i ESCs activated the largest number of miRNA genes. Since chromosomes 2 and 12 showed a significant difference in the number of miRNA genes they activated under different cultures, we focused on these two chromosomes and found, interestingly, that most members of a repetitive miRNA cluster within the 10th intron of the imprinted *Sfmbt2* gene located on chromosome 2 were expressed much higher in serum compared with 2i/R2i (Figure 4B). Moreover, we found that approximately all members of a large miRNA cluster embedded in an imprinted region overlapping with the developmentally important *Dlk1-Dio3* locus on chromosome 12 (12qF1) were expressed much higher in 2i/R2i than in serum (Figure 4C). Surprisingly, these *Dlk1-Dio3* locus-embedded miRNAs constituted the majority of miRNAs upregulated in ground-state cells (Figure S4B). In conclusion, we uncovered a ground-state-specific, imprinted genomic region coding for miRNAs that can serve as a signature for ground-state pluripotency and might be of functional importance.

Ground-State-Associated miRNAs Promote ESC Self-Renewal

The association of chromosome 12-located miRNA gene expression with ground-state ESCs raised the intriguing question of whether these miRNAs directly affect acquisition or maintenance of the ground state. To evaluate this possibility, we selected three ground-state-associated miRNAs located within the imprinted *Dlk1-Dio3* locus (miR-541-5p, miR-410-3p, and miR-381-3p) based on the following: (1) their high abundance compared with other ground-state-specific miRNAs (see Figure 3C) and (2) their putative ability to target diverse differentiation pathways (Tables S8). After confirmation of efficient delivery of the candidate miRNAs into cultured mouse embryonic fibroblasts (Figure S4C), we treated undifferentiated serum-grown ESCs with individual miRNAs and analyzed morphology and expression of different stemness and differentiation genes 3 days after transfection. ESCs treated with candidate miRNAs exhibited typical ESC colony formation with more compact morphology compared with the Scr control (Figure 5A). qRT-PCR analysis revealed that miRNA-transfected ESCs expressed higher levels of stemness genes and lower levels of differentiation genes (Figure 5B). In addition, we found that miR-541-5p, miR-410-3p, and miR-381-3p promoted the viability of ESCs 3 days post treatment as measured by the MTS assay (Figure 5C). Moreover, transfection of miR-541-5p, miR-410-3p, and miR-381-3p into ground-state R2i ESCs cultured for 3 days in the absence of R2i/LIF (a condition that considerably reduces ESC viability) significantly enhanced viability (Figure 5D). Additional assessments of viability using the Live/Dead Viability/Cytotoxicity Kit validated these results (Figure 5E). Furthermore, we observed an increase of the number of alkaline phosphatase (AP)-positive ESC colonies 5 days post transfection in both the presence (Figure 5F) and absence of LIF (Figure 5G). Notably, overexpression of miRNAs also enhanced AP activity in ESCs cultured without LIF for 5 days (Figure S4D), further

Figure 5. Effect of Ground-State-Associated miRNAs on ESC Self-Renewal

(A) Phase-contrast image of serum ESCs treated with representative ground-state miRNAs. Scale bar, 100 μm.

(B) qRT-PCR analysis of pluripotency- and differentiation-associated transcripts 3 days following serum ESC treatment with ground-state miRNAs. *Gapdh* was used as an internal normalization control. Data are shown as mean ± SD, n = 3. *p < 0.05.

(C) MTS assay of ESCs 3 days after transfection of miRNAs. Data are shown as mean ± SD, n = 3. *p = 0.0311, **p = 0.0029, ***p = 0.0007.

(D) MTS assay of ESCs 3 days after transfection with miRNAs in the presence or absence of R2i + LIF. Data are shown as mean ± SD, n = 3. *p = 0.0111, **p < 0.0025.

(E) Live/dead staining of ESCs 3 days after transfection with miRNAs in the presence or absence of R2i + LIF. Scale bars, 100 μm.

(F) Number of AP-positive ESC colonies grown in the presence of LIF 5 days after transfection of miRNAs. Data are shown as mean ± SD, n = 4. *p = 0.0295, **p = 0.0015, ***p = 0.0004.

(G) Number of AP-positive ESC colonies grown in the absence of LIF 5 days after transfection of miRNAs. Data are shown as mean ± SD, n = 4. **p = 0.0032, ***p = 0.001, ****p < 0.0001.

(H) ESC clonogenicity assay following serum ESC treatment with miRNAs. Cells were stained for AP activity 8 days after seeding. AP-positive and -negative colonies were counted under the microscope. Data are shown as mean ± SD, n = 3. **p = 0.0017, ***p = 0.0002, ****p < 0.0001.

(I) AP staining of serum ESCs transfected with miRNAs on day 8. Scale bar, 50 μm.

(J) Cell-cycle profiling of serum ESCs treated with miRNAs using flow cytometry 3 days after transfection. Scr (serum ESCs without LIF at the presence of a control scrambled oligonucleotide) is the negative control and serum + LIF is the positive control. miRNA mimics were added to LIF-withdrawn ESCs, which allowed us to score the effects of miRNA mimics relative to the positive and negative controls. Data are shown as mean ± SD, n = 3. *p < 0.05.

(K) Ratio of cells in sub-G_1 phase of the cell cycle 3 days following serum ESC treatment with miRNAs based on flow-cytometry analysis of the cell cycle. Data are shown as mean ± SD, n = 3. *p < 0.05, **p < 0.0065.

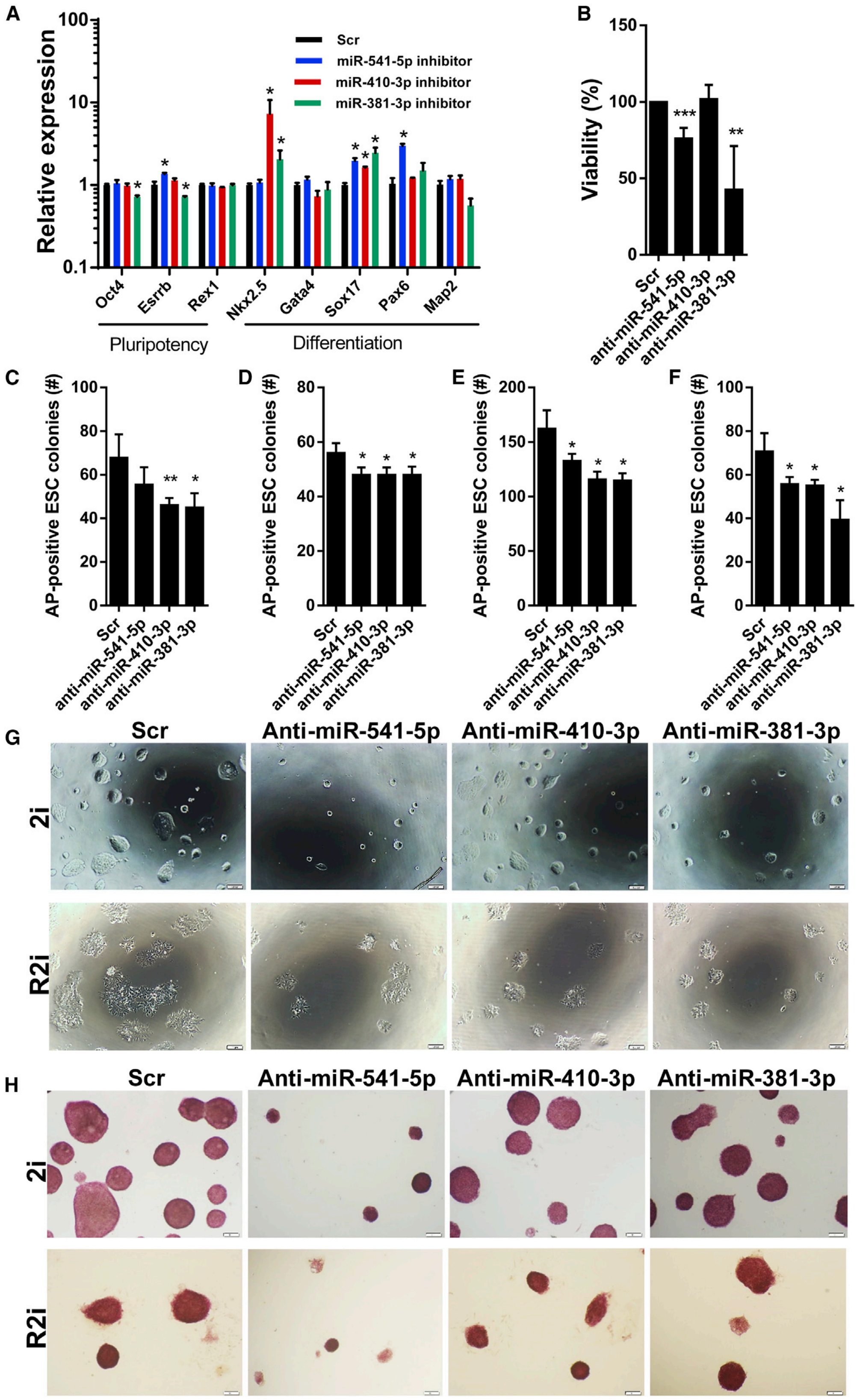

(legend on next page)

supporting our conclusion that ground-state-associated miRNAs promote pluripotency and partially "rescue" LIF-free ESCs from differentiation.

Since ground-state-specific miRNAs promoted ESC viability and 2i and R2i cells exhibit a much higher cloning efficiency than serum cells (Hassani et al., 2014b), we wanted to determine the colony-forming ability of ESCs transfected with miR-541-5p, miR-410-3p, and miR-381-3p. Five days after reseeding of day-3 transfected serum ESCs, we analyzed AP activity by counting AP-positive and -negative colonies and observed a significant increase of the number of AP-positive colonies by all three miRNAs but particularly by miR-541-5p (Figure 5H), as well as a general enhancement of AP staining intensity (Figure 5I). These results indicated that ground-state-associated miRNAs boosted AP activity and markedly promoted ESC clonogenicity.

Ground-state miRNAs might influence ESC viability and clonogenicity by modulating the ESC cycle. We focused specifically on the G_1 phase, which is shortened in ESCs compared with differentiated cells and the extension of G_1 phase upon differentiation (Becker et al., 2006, 2010; Calder et al., 2013; Coronado et al., 2013; Li et al., 2012). To induce ESC differentiation, we removed LIF from the cultures and concomitantly added miRNAs. Three days after transfection, differentiating ESCs were analyzed by flow cytometry. As expected, we observed prolonged G_0/G_1 phases in Scr-treated control cells after LIF withdrawal, indicative of the exit from pluripotency. In contrast, transfection with either miR-541-5p, miR-410-3p, or miR-381-3p shortened the prolonged G_1 phase of differentiating ESCs, which reached values characteristic for LIF-grown ESCs (Figure 5J). Similarly, all three miRNAs reduced the sub-G_1 phase under LIF-free conditions (Figure 5K), which implicates improved survival upon initiation of differentiation.

To corroborate our results, we asked whether inhibition of identified miRNAs will disrupt the ESC ground state. Transfection of ground-state ESCs with miR-541-5p, miR-410-3p, and miR-381-3p antagomirs induced upregulation of differentiation-associated genes 3 days after treatment (Figure 6A). Inhibition of miR-541-5p and miR-381-3p also reduced cellular viability in MTS assays (Figure 6B). Moreover, inhibition of ground-state miRNAs not only reduced the number of AP-positive 2i/R2i ESC colonies 3 days after miRNA inhibitor delivery (Figures 6C and 6D) but also the clonogenicity of the cells 8 days post transfection (Figures 6E and 6F). Of note, we also observed that inhibition of miR-541-5p remarkably decreased the colony size in both 2i and R2i ESCs 3 days (Figure 6G) and 8 days post transfection (Figure 6H). Taken together, our data indicate that miR-541-5p, miR-410-3p, and miR-381-3p promote ESC self-renewal.

Ground-State-Associated miRNAs Inhibit Multi-lineage Differentiation of ESCs

We hypothesized that miR-541-5p, miR-410-3p, and miR-381-3p might contribute to the maintenance of ground-state ESCs by inhibiting differentiation, which would fit into the observed downregulation of these miRNAs 7 days after initiation of differentiation (Figure 7A). For analysis of the effects of miR-541-5p, miR-410-3p, and miR-381-3p on ESC differentiation, undifferentiated serum ESCs were seeded 1 day prior to miRNA transfection and ESCs were induced to form embryoid bodies (EBs) in LIF-free medium 1 day after transfection. EBs were harvested at day 7 and subjected to qRT-PCR expression analysis of key genes representing pluripotency and differentiation.

Interestingly, we found that all candidate miRNAs repressed the majority of genes characteristic for ESC differentiation into different germ layers (Figure 7B), suggesting that they block multi-lineage differentiation of ESCs. We also detected increased numbers of AP-positive EBs compared with the Scr control 7 days after differentiation (Figure 7C). Similar to our previous results with undifferentiated ESCs cultured in monolayers, we observed increased AP activity of miRNA-treated EBs (Figure 7D). Next, we examined whether inhibition of ground-state miRNAs stimulates multi-lineage differentiation of ground-state ESCs. To this end, we treated the cells with miRNA inhibitors 2 days prior to EB induction and then harvested the cells for qRT-PCR analysis 10 days post treatment. We

Figure 6. Effect of Ground-State-Associated miRNA Inhibitors on ESC Self-Renewal

(A) qRT-PCR analysis of pluripotency- and differentiation-associated transcripts 3 days following 2i ESC treatment with the inhibitors of ground-state miRNAs. *Gapdh* was used as an internal normalization control. Data are shown as mean ± SD, n = 3. *p < 0.05.

(B) MTS assay of 2i ESCs 3 days after transfection of miRNA inhibitors. Data are shown as mean ± SD, n = 3. **p = 0.0029, ***p = 0.0007.

(C) Number of AP-positive 2i ESC colonies 3 days after transfection of miRNA inhibitors. Data are shown as mean ± SD, n = 3. *p < 0.05, **p = 0.0015.

(D) Number of AP-positive R2i ESC colonies 3 days after transfection of miRNA inhibitors. Data are shown as mean ± SD, n = 3. *p < 0.05.

(E) 2i ESC clonogenicity assay 8 days following treatment with miRNA inhibitors. Data are shown as mean ± SD, n = 3. *p < 0.05.

(F) R2i ESC clonogenicity assay 8 days following treatment with miRNA inhibitors. Data are shown as mean ± SD, n = 3. *p < 0.05.

(G) Phase-contrast image of 2i and R2i ESC colonies 3 days after treatment with miRNA inhibitors. Scale bars, 100 μm.

(H) AP staining of 2i and R2i ESC colonies 8 days after treatment with miRNA inhibitors. Scale bars, 100 μm.

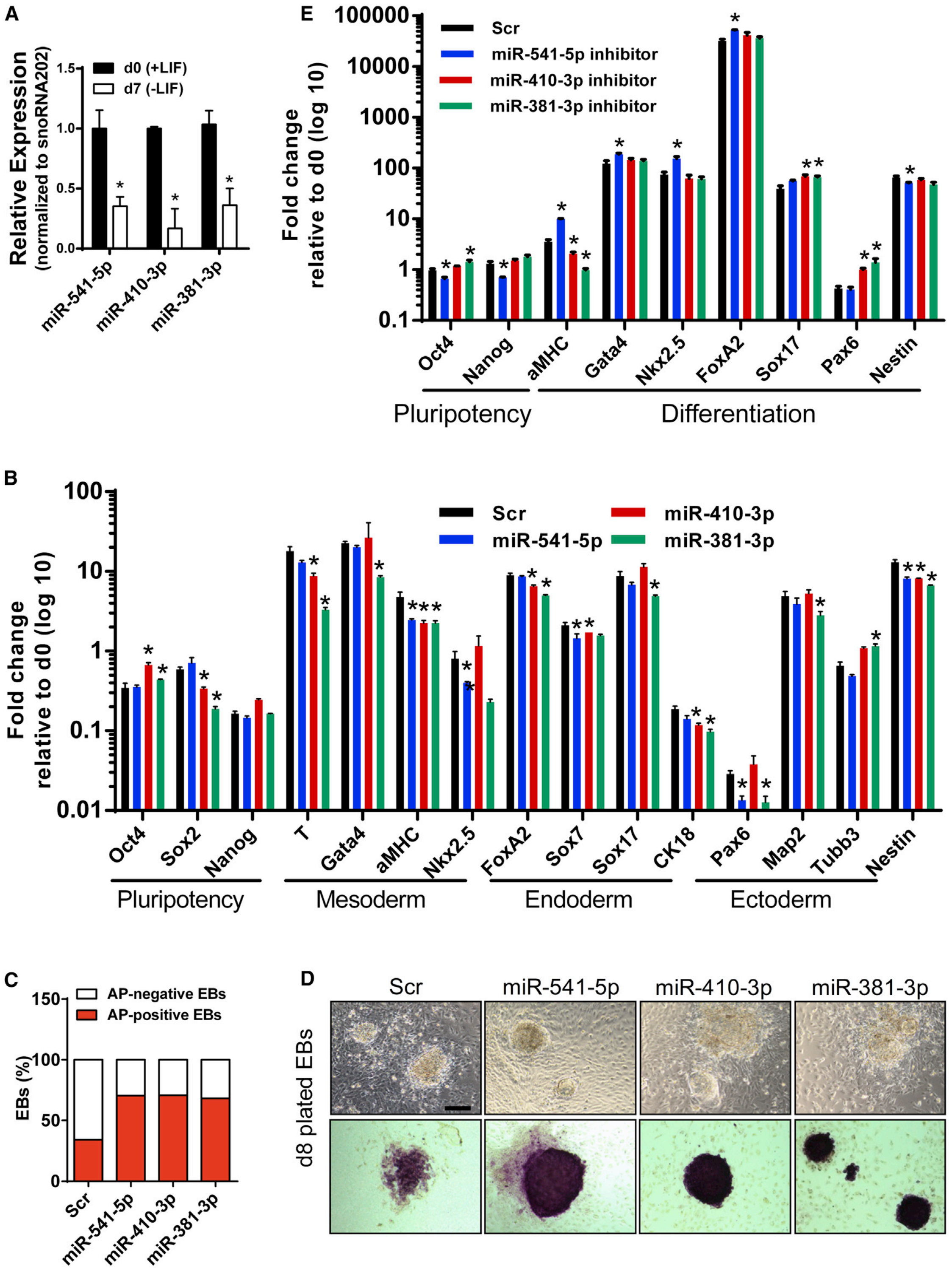

Figure 7. Effect of Ground-State-Associated miRNAs on ESC Differentiation

(A) qRT-PCR analysis of miRNAs 7 days after induction of EB formation. snoRNA202 was used as an internal normalization control. Data are shown as mean ± SD, n = 3. *p < 0.0001.

(B) qRT-PCR analysis of transcripts associated with pluripotency and differentiation 7 days following serum ESC treatment with miRNAs in the absence of LIF. *Gapdh* was used as an internal normalization control. Data are shown as mean ± SD, n = 3. *p < 0.05.

(legend continued on next page)

observed a significant upregulation of most of the tested differentiation-associated genes (Figure 7E), which is consistent with the data obtained using miRNA mimics. Taken together, our results indicate that miR-541-5p, miR-410-3p, and miR-381-3p contribute to the maintenance of ESC pluripotency and self-renewal by blocking differentiation.

DNA Methylation Status of the *Dlk1-Dio3* Locus

Since (1) most differentially expressed miRNA genes are located in imprinted regions of the genome and (2) serum cells own higher DNA methylation levels than 2i (Ficz et al., 2013; Habibi et al., 2013; Yamaji et al., 2013), we reasoned that differences in DNA methylation might contribute to differential miRNA gene expression from the *Dlk1-Dio3* locus in ESCs. To test this hypothesis, we reanalyzed previously published DNA methylome data of serum and 2i cells (Angermueller et al., 2016). The average DNA methylation ratio in serum and 2i ESCs at the *Dlk1-Dio3* locus was accounted for 73.2% and 37.6%, respectively (Figure S5A), indicating that the *Dlk1-Dio3* locus is hypomethylated in 2i compared with serum. To confirm this result, we subjected DNA from serum, 2i, and R2i ESCs to methylation-sensitive restriction enzyme digestion. The resulting genomic fragments flanking specific restriction sites within the *Dlk1-Dio3* locus were analyzed by qPCR using specific primers. We found that ground-state ESCs compared with serum cells exhibited a significantly lower DNA methylation level at the three differentially methylated regions present in the *Dlk1-Dio3* locus (Figure S5B). We hypothesize that the higher DNA methylation levels at the *Dlk1-Dio3* locus in serum cells explain the significantly lower expression of miRNAs embedded in this imprinted region.

Putative Target Genes of miR-541-5p, miR-410-3p, and miR-381-3p

We next wanted to determine potential target genes of the candidate miRNAs. To achieve this goal, we intersected putative miRNA targets predicted by TargetScan (Table S7) with proteins that were downregulated in 2i and R2i ESCs relative to serum cells (Taleahmad et al., 2015; H.B., unpublished data). This approach narrowed the putative target list down to 10, 4, and 5 genes for miR-541-5p, miR-410-3p, and miR-381-3p, respectively (Figure S6A). Using qRT-PCR, we confirmed that miR-541-5p overexpression reduced prolyl 4-hydroxylase (*P4ha1*) (Figure S6B), miR-410-3p overexpression reduced serine-arginine rich splicing factor 11 (*Srsf11*) and poly(C)-binding protein 2 (*Pcbp2*) (Figure S6C), and miR-381-3p overexpression downregulated microtubule-associated protein 4 (*Map4*) (Figure S6D). *P4ha1* codes for a key enzyme implicated in the synthesis of collagen, which has been shown to function as a barrier in iPSC generation (Jiao et al., 2013). The extracellular matrix (ECM) is of critical importance to ESC maintenance, and the expression of collagens and other ECM components have been reported to be increased upon exit from ground-state pluripotency (Veluscek et al., 2016). *Srsf11* is a nuclear protein involved in pre-mRNA splicing (Shepard and Hertel, 2009; Zhang and Wu, 1996). *Pcbp2* encodes an RNA binding protein that regulates pre-mRNA splicing in the nucleus and mRNA stabilization in the cytoplasm (Ji et al., 2007, 2011; Makeyev and Liebhaber, 2002) and is implicated in the regulation of collagen synthesis (Stefanovic et al., 1997). *Map4* is a non-neuronal microtubule-interacting protein promoting tubulin polymerization (Nguyen et al., 1999). Map4 phosphorylation modulates microtubule assembly and dynamics, and cell cycling (Chang et al., 2002; Illenberger et al., 1996; Kitazawa et al., 2000). Although further investigation is required to uncover the exact function of these genes in serum and ground-state pluripotency, we can speculate that the *Dlk1-Dio3* locus-embedded miRNAs contribute to the maintenance of ground-state pluripotency by regulating ECM, cytoskeletal dynamics, RNA processing, and cell cycling.

DISCUSSION

Small RNA sequencing has been used to profile miRNAs in different plant and animal species, diverse cell types, and human tissue fluids (Bellingham et al., 2012; Chiang et al., 2010; Glazov et al., 2008; Landgraf et al., 2007; Sunkar et al., 2005; Vojtech et al., 2014), but similar datasets for ground-state ESCs have been missing so far. In the present study, we used small RNA sequencing to obtain expression profiles of miRNAs in ground-state (2i and R2i) ESCs versus serum ESCs. Several miRNAs were differentially expressed between ground-state ESCs and serum ESCs. Most miRNAs upregulated in both 2i and R2i ESCs are located within the *Dlk1-Dio3* locus. Interestingly, activity of the *Dlk1-Dio3* locus seems crucial to maintain pluripotency of iPSCs, and its reduced expression is associated with incomplete iPSC reprogramming and poor contribution to mouse chimeras (Liu et al., 2010; Stadtfeld et al., 2010). We assume

(C) Percentages of AP-positive and -negative EBs 7 days after transfection of ESCs in the absence of LIF.
(D) AP staining of EBs treated with miRNAs 7 days post differentiation. Scale bar, 200 μm.
(E) qRT-PCR analysis of gene transcripts associated with pluripotency and differentiation 7 days following 2i ESC treatment with miRNA inhibitors in the absence of 2i/LIF. *Gapdh* was used as an internal normalization control. Data are shown as mean ± SD, n = 3. *p < 0.05.

that ESCs undergo a gradual decline of *Dlk1-Dio3* locus activity during the transition from ground to serum state and differentiation.

Interestingly, the majority of miRNAs that are preferentially expressed in serum compared with 2i and R2i ESCs are located in the imprinted locus *Sfmbt2*. Our study, therefore, identifies large sets of differentially expressed miRNAs at imprinted loci, which can serve as specific markers of ground-state pluripotency versus serum state. The lower DNA methylation level observed at the *Dlk1-Dio3* locus in ground-state cells might lead to a more accessible chromatin for pluripotency factors to bind and activate the miRNAs embedded in the locus. Of note, the imprinted *Sfmbt2* locus, which harbors a large miRNA cluster, does not seem to carry a classic DMR (Wang et al., 2011), suggesting that different mechanisms regulate the expression of miRNAs embedded in the *Sfmbt2* locus.

Results from a previous study indicated that miRNAs from the *Sfmbt2* locus are expressed at higher levels in serum ESCs compared with epiblast stem cells (EpiSCs) (Jouneau et al., 2012). Since we found higher levels of *Sfmbt2*-embedded miRNAs in serum ESCs versus 2i/R2i, it is tempting to speculate that expression of miRNAs embedded within *Sfmbt2* locus presents a key miRNA signature of naive pluripotency allowing distinction of different mouse pluripotent states.

We demonstrated using miRNA gain- and loss-of-function analyses that ground-state miRNAs within the *Dlk1-Dio3* locus contribute to the maintenance of ground-state pluripotency by enhancing viability, clonogenicity, AP activity, and ESC cycling as well as inhibiting differentiation. Our analysis revealed that the putative targets of miR-541-5p, miR-410-3p, and miR-381-3p are involved in cytoskeletal organization, ECM dynamics, control of RNA processing/decay, and cell cycling. These processes have previously been found to be important for pluripotency in general and ground-state pluripotency in particular (Chowdhury et al., 2010; Dalton, 2015; Ji and Tian, 2009; Kalkan et al., 2017; Salomonis et al., 2010; Taleahmad et al., 2015; Veluscek et al., 2016). Our findings provide a useful perspective to temporarily lock ESCs in the ground state either by exogenous introduction of cell-permeable miRNAs or by the identification of small molecules that increase ground-state miRNA expression.

EXPERIMENTAL PROCEDURES

Mouse ESC lines were cultivated on gelatinized tissue-culture plates and dishes (Sigma-Aldrich) and passaged every other day. Serum ESCs were cultured in the presence of 15% ES-qualified fetal bovine serum (HyClone), and 2i/R2i cells were cultured in serum-free N2B27 medium.

Additional experimental procedures are detailed in Supplemental Experimental Procedures.

ACCESSION NUMBERS

The processed data of small RNA sequencing is deposited in the NCBI Gene Expression Omnibus (GEO: GSE87174).

SUPPLEMENTAL INFORMATION

Supplemental Information includes Supplemental Experimental Procedures, six figures, and eight tables and can be found with this article online at https://doi.org/10.1016/j.stemcr.2017.10.009.

AUTHOR CONTRIBUTIONS

H.B. and S. Moradi conceived and designed the study. S. Moradi, H.B., and T.B. designed experiments and analyzed and interpreted the data. S. Moradi performed most of the experiments and wrote the manuscript. H.B., T.B., and S.A. provided financial and administrative support, discussed the results, and approved the manuscript. S. Mollamohammadi and A.S. contributed to cell-culture and cell-cycle analysis. A.S.-Z. performed bioinformatics analysis. A.A. contributed to qRT-PCR and DNA methylation analysis. S.A., G.H.S., and S.G. contributed to data analysis and interpretation. All authors reviewed and confirmed the manuscript before submission.

ACKNOWLEDGMENTS

This work was supported by a grant from the Royan Institute, the Iranian Council of Stem Cell Research and Technology, the Iran National Science Foundation (INSF), and the Iran Science Elites Federation to H.B. and a grant from National Health and Medical Research (APP1062983) to S.A. T.B. was supported by grants from the Deutsche Forschungsgemeinschaft (ExcellenceCluster Cardio-pulmonary System, SFB TRR81 TP A02 and SFB 1213 TP A02 and TP B02), the LOEWE Center for Cell and Gene Therapy, and the German Center for Cardiovascular Research. We thank Dr. Hamidreza Chitsaz, Colorado State University, for the computational facility. We also thank the Institute for Research in Fundamental Sciences (IPM), School of Biological Sciences, Tehran for data analysis.

Received: October 8, 2016
Revised: October 11, 2017
Accepted: October 11, 2017
Published: November 9, 2017

REFERENCES

Angermueller, C., Clark, S.J., Lee, H.J., Macaulay, I.C., Teng, M.J., Hu, T.X., Krueger, F., Smallwood, S.A., Ponting, C.P., Voet, T., et al. (2016). Parallel single-cell sequencing links transcriptional and epigenetic heterogeneity. Nat. Methods *13*, 229–232.

Baek, D., Villen, J., Shin, C., Camargo, F.D., Gygi, S.P., and Bartel, D.P. (2008). The impact of microRNAs on protein output. Nature *455*, 64–71.

Bartel, D.P. (2004). MicroRNAs: genomics, biogenesis, mechanism, and function. Cell *116*, 281–297.

Bartel, D.P. (2009). MicroRNAs: target recognition and regulatory functions. Cell *136*, 215–233.

Becker, K.A., Ghule, P.N., Therrien, J.A., Lian, J.B., Stein, J.L., van Wijnen, A.J., and Stein, G.S. (2006). Self-renewal of human embryonic stem cells is supported by a shortened G1 cell cycle phase. J. Cell. Physiol. *209*, 883–893.

Becker, K.A., Stein, J.L., Lian, J.B., van Wijnen, A.J., and Stein, G.S. (2010). Human embryonic stem cells are pre-mitotically committed to self-renewal and acquire a lengthened G1 phase upon lineage programming. J. Cell. Physiol. *222*, 103–110.

Bellingham, S.A., Coleman, B.M., and Hill, A.F. (2012). Small RNA deep sequencing reveals a distinct miRNA signature released in exosomes from prion-infected neuronal cells. Nucleic Acids Res. *40*, 10937–10949.

Calder, A., Roth-Albin, I., Bhatia, S., Pilquil, C., Lee, J.H., Bhatia, M., Levadoux-Martin, M., McNicol, J., Russell, J., Collins, T., et al. (2013). Lengthened G1 phase indicates differentiation status in human embryonic stem cells. Stem Cells Dev. *22*, 279–295.

Chang, S.Y., Di, A., Naren, A.P., Palfrey, H.C., Kirk, K.L., and Nelson, D.J. (2002). Mechanisms of CFTR regulation by syntaxin 1A and PKA. J. Cell Sci. *115*, 783–791.

Chiang, H.R., Schoenfeld, L.W., Ruby, J.G., Auyeung, V.C., Spies, N., Baek, D., Johnston, W.K., Russ, C., Luo, S., Babiarz, J.E., et al. (2010). Mammalian microRNAs: experimental evaluation of novel and previously annotated genes. Genes Dev. *24*, 992–1009.

Chowdhury, F., Li, Y., Poh, Y.C., Yokohama-Tamaki, T., Wang, N., and Tanaka, T.S. (2010). Soft substrates promote homogeneous self-renewal of embryonic stem cells via downregulating cell-matrix tractions. PLoS One *5*, e15655.

Coronado, D., Godet, M., Bourillot, P.Y., Tapponnier, Y., Bernat, A., Petit, M., Afanassieff, M., Markossian, S., Malashicheva, A., Iacone, R., et al. (2013). A short G1 phase is an intrinsic determinant of naive embryonic stem cell pluripotency. Stem Cell Res. *10*, 118–131.

Dalton, S. (2015). Linking the cell cycle to cell fate decisions. Trends Cell Biol. *25*, 592–600.

Evans, M.J., and Kaufman, M.H. (1981). Establishment in culture of pluripotential cells from mouse embryos. Nature *292*, 154–156.

Ficz, G., Hore, T.A., Santos, F., Lee, H.J., Dean, W., Arand, J., Krueger, F., Oxley, D., Paul, Y.L., Walter, J., et al. (2013). FGF signaling inhibition in ESCs drives rapid genome-wide demethylation to the epigenetic ground state of pluripotency. Cell Stem Cell *13*, 351–359.

Glazov, E.A., Cottee, P.A., Barris, W.C., Moore, R.J., Dalrymple, B.P., and Tizard, M.L. (2008). A microRNA catalog of the developing chicken embryo identified by a deep sequencing approach. Genome Res. *18*, 957–964.

Graham, B., Marcais, A., Dharmalingam, G., Carroll, T., Kanellopoulou, C., Graumann, J., Nesterova, T.B., Bermange, A., Brazauskas, P., Xella, B., et al. (2016). MicroRNAs of the miR-290-295 family maintain bivalency in mouse embryonic stem cells. Stem Cell Rep. *6*, 635–642.

Habibi, E., Brinkman, A.B., Arand, J., Kroeze, L.I., Kerstens, H.H., Matarese, F., Lepikhov, K., Gut, M., Brun-Heath, I., Hubner, N.C., et al. (2013). Whole-genome bisulfite sequencing of two distinct interconvertible DNA methylomes of mouse embryonic stem cells. Cell Stem Cell *13*, 360–369.

Hadjimichael, C., Nikolaou, C., Papamatheakis, J., and Kretsovali, A. (2016). MicroRNAs for fine-tuning of mouse embryonic stem cell fate decision through regulation of TGF-beta signaling. Stem Cell Rep. *6*, 292–301.

Hassani, S.N., Pakzad, M., Asgari, B., Taei, A., and Baharvand, H. (2014a). Suppression of transforming growth factor beta signaling promotes ground state pluripotency from single blastomeres. Hum. Reprod. *29*, 1739–1748.

Hassani, S.N., Totonchi, M., Sharifi-Zarchi, A., Mollamohammadi, S., Pakzad, M., Moradi, S., Samadian, A., Masoudi, N., Mirshahvaladi, S., Farrokhi, A., et al. (2014b). Inhibition of TGFbeta signaling promotes ground state pluripotency. Stem Cell Rev. *10*, 16–30.

Houbaviy, H.B., Murray, M.F., and Sharp, P.A. (2003). Embryonic stem cell-specific MicroRNAs. Dev. Cell *5*, 351–358.

Illenberger, S., Drewes, G., Trinczek, B., Biernat, J., Meyer, H.E., Olmsted, J.B., Mandelkow, E.M., and Mandelkow, E. (1996). Phosphorylation of microtubule-associated proteins MAP2 and MAP4 by the protein kinase p110mark. Phosphorylation sites and regulation of microtubule dynamics. J. Biol. Chem. *271*, 10834–10843.

Ji, X., Kong, J., Carstens, R.P., and Liebhaber, S.A. (2007). The 3′ untranslated region complex involved in stabilization of human alpha-globin mRNA assembles in the nucleus and serves an independent role as a splice enhancer. Mol. Cell. Biol. *27*, 3290–3302.

Ji, X., Kong, J., and Liebhaber, S.A. (2011). An RNA-protein complex links enhanced nuclear 3′ processing with cytoplasmic mRNA stabilization. EMBO J. *30*, 2622–2633.

Ji, Z., and Tian, B. (2009). Reprogramming of 3′ untranslated regions of mRNAs by alternative polyadenylation in generation of pluripotent stem cells from different cell types. PLoS One *4*, e8419.

Jiao, J., Dang, Y., Yang, Y., Gao, R., Zhang, Y., Kou, Z., Sun, X.F., and Gao, S. (2013). Promoting reprogramming by FGF2 reveals that the extracellular matrix is a barrier for reprogramming fibroblasts to pluripotency. Stem Cells *31*, 729–740.

Jouneau, A., Ciaudo, C., Sismeiro, O., Brochard, V., Jouneau, L., Vandormael-Pournin, S., Coppee, J.Y., Zhou, Q., Heard, E., Antoniewski, C., et al. (2012). Naive and primed murine pluripotent stem cells have distinct miRNA expression profiles. RNA *18*, 253–264.

Kalkan, T., Olova, N., Roode, M., Mulas, C., Lee, H.J., Nett, I., Marks, H., Walker, R., Stunnenberg, H.G., Lilley, K.S., et al. (2017). Tracking the embryonic stem cell transition from ground state pluripotency. Development *144*, 1221–1234.

Kanellopoulou, C., Muljo, S.A., Kung, A.L., Ganesan, S., Drapkin, R., Jenuwein, T., Livingston, D.M., and Rajewsky, K. (2005). Dicer-deficient mouse embryonic stem cells are defective in differentiation and centromeric silencing. Genes Dev. *19*, 489–501.

Kitazawa, H., Iida, J., Uchida, A., Haino-Fukushima, K., Itoh, T.J., Hotani, H., Ookata, K., Murofushi, H., Bulinski, J.C., Kishimoto, T., et al. (2000). Ser787 in the proline-rich region of human MAP4 is a critical phosphorylation site that reduces its activity to promote tubulin polymerization. Cell Struct. Funct. *25*, 33–39.

Kumar, R.M., Cahan, P., Shalek, A.K., Satija, R., DaleyKeyser, A.J., Li, H., Zhang, J., Pardee, K., Gennert, D., Trombetta, J.J., et al. (2014). Deconstructing transcriptional heterogeneity in pluripotent stem cells. Nature *516*, 56–61.

Landgraf, P., Rusu, M., Sheridan, R., Sewer, A., Iovino, N., Aravin, A., Pfeffer, S., Rice, A., Kamphorst, A.O., Landthaler, M., et al. (2007). A mammalian microRNA expression atlas based on small RNA library sequencing. Cell *129*, 1401–1414.

Li, V.C., Ballabeni, A., and Kirschner, M.W. (2012). Gap 1 phase length and mouse embryonic stem cell self-renewal. Proc. Natl. Acad. Sci. USA *109*, 12550–12555.

Liu, L., Luo, G.Z., Yang, W., Zhao, X., Zheng, Q., Lv, Z., Li, W., Wu, H.J., Wang, L., Wang, X.J., et al. (2010). Activation of the imprinted Dlk1-Dio3 region correlates with pluripotency levels of mouse stem cells. J. Biol. Chem. *285*, 19483–19490.

Liu, Q., Wang, G., Chen, Y., Li, G., Yang, D., and Kang, J. (2014). A miR-590/Acvr2a/Rad51b axis regulates DNA damage repair during mESC proliferation. Stem Cell Rep. *3*, 1103–1117.

Makeyev, A.V., and Liebhaber, S.A. (2002). The poly(C)-binding proteins: a multiplicity of functions and a search for mechanisms. RNA *8*, 265–278.

Marks, H., Kalkan, T., Menafra, R., Denissov, S., Jones, K., Hofemeister, H., Nichols, J., Kranz, A., Stewart, A.F., Smith, A., et al. (2012). The transcriptional and epigenomic foundations of ground state pluripotency. Cell *149*, 590–604.

Marson, A., Levine, S.S., Cole, M.F., Frampton, G.M., Brambrink, T., Johnstone, S., Guenther, M.G., Johnston, W.K., Wernig, M., Newman, J., et al. (2008). Connecting microRNA genes to the core transcriptional regulatory circuitry of embryonic stem cells. Cell *134*, 521–533.

Martin, G.R. (1981). Isolation of a pluripotent cell line from early mouse embryos cultured in medium conditioned by teratocarcinoma stem cells. Proc. Natl. Acad. Sci. USA *78*, 7634–7638.

Melton, C., Judson, R.L., and Blelloch, R. (2010). Opposing microRNA families regulate self-renewal in mouse embryonic stem cells. Nature *463*, 621–626.

Moradi, S., Asgari, S., and Baharvand, H. (2014). Concise review: harmonies played by microRNAs in cell fate reprogramming. Stem Cells *32*, 3–15.

Nguyen, H.L., Gruber, D., and Bulinski, J.C. (1999). Microtubule-associated protein 4 (MAP4) regulates assembly, protomer-polymer partitioning and synthesis of tubulin in cultured cells. J. Cell Sci. *112* (Pt 12), 1813–1824.

Parchem, R.J., Moore, N., Fish, J.L., Parchem, J.G., Braga, T.T., Shenoy, A., Oldham, M.C., Rubenstein, J.L., Schneider, R.A., and Blelloch, R. (2015). miR-302 is required for timing of neural differentiation, neural tube closure, and embryonic viability. Cell Rep. *12*, 760–773.

Salomonis, N., Schlieve, C.R., Pereira, L., Wahlquist, C., Colas, A., Zambon, A.C., Vranizan, K., Spindler, M.J., Pico, A.R., Cline, M.S., et al. (2010). Alternative splicing regulates mouse embryonic stem cell pluripotency and differentiation. Proc. Natl. Acad. Sci. USA *107*, 10514–10519.

Sayed, D., and Abdellatif, M. (2011). MicroRNAs in development and disease. Physiol. Rev. *91*, 827–887.

Shepard, P.J., and Hertel, K.J. (2009). The SR protein family. Genome Biol. *10*, 242.

Stadtfeld, M., Apostolou, E., Akutsu, H., Fukuda, A., Follett, P., Natesan, S., Kono, T., Shioda, T., and Hochedlinger, K. (2010). Aberrant silencing of imprinted genes on chromosome 12qF1 in mouse induced pluripotent stem cells. Nature *465*, 175–181.

Stefanovic, B., Hellerbrand, C., Holcik, M., Briendl, M., Aliebhaber, S., and Brenner, D.A. (1997). Posttranscriptional regulation of collagen alpha1(I) mRNA in hepatic stellate cells. Mol. Cell. Biol. *17*, 5201–5209.

Sunkar, R., Girke, T., Jain, P.K., and Zhu, J.K. (2005). Cloning and characterization of microRNAs from rice. Plant Cell *17*, 1397–1411.

Taleahmad, S., Mirzaei, M., Parker, L.M., Hassani, S.N., Mollamohammadi, S., Sharifi-Zarchi, A., Haynes, P.A., Baharvand, H., and Salekdeh, G.H. (2015). Proteome analysis of ground state pluripotency. Sci. Rep. *5*, 17985.

Tay, Y., Zhang, J., Thomson, A.M., Lim, B., and Rigoutsos, I. (2008). MicroRNAs to Nanog, Oct4 and Sox2 coding regions modulate embryonic stem cell differentiation. Nature *455*, 1124–1128.

Veluscek, G., Li, Y., Yang, S.H., and Sharrocks, A.D. (2016). Jun-mediated changes in cell adhesion contribute to mouse embryonic stem cell exit from ground state pluripotency. Stem Cells *34*, 1213–1224.

Vojtech, L., Woo, S., Hughes, S., Levy, C., Ballweber, L., Sauteraud, R.P., Strobl, J., Westerberg, K., Gottardo, R., Tewari, M., et al. (2014). Exosomes in human semen carry a distinctive repertoire of small non-coding RNAs with potential regulatory functions. Nucleic Acids Res. *42*, 7290–7304.

Wang, Q., Chow, J., Hong, J., Smith, A.F., Moreno, C., Seaby, P., Vrana, P., Miri, K., Tak, J., Chung, E.D., et al. (2011). Recent acquisition of imprinting at the rodent Sfmbt2 locus correlates with insertion of a large block of miRNAs. BMC Genomics *12*, 204.

Wang, Y., Baskerville, S., Shenoy, A., Babiarz, J.E., Baehner, L., and Blelloch, R. (2008). Embryonic stem cell-specific microRNAs regulate the G1-S transition and promote rapid proliferation. Nat. Genet. *40*, 1478–1483.

Wang, Y., Medvid, R., Melton, C., Jaenisch, R., and Blelloch, R. (2007). DGCR8 is essential for microRNA biogenesis and silencing of embryonic stem cell self-renewal. Nat. Genet. *39*, 380–385.

Wray, J., Kalkan, T., and Smith, A.G. (2010). The ground state of pluripotency. Biochem. Soc. Trans. *38*, 1027–1032.

Yamaji, M., Ueda, J., Hayashi, K., Ohta, H., Yabuta, Y., Kurimoto, K., Nakato, R., Yamada, Y., Shirahige, K., and Saitou, M. (2013). PRDM14 ensures naive pluripotency through dual regulation of signaling and epigenetic pathways in mouse embryonic stem cells. Cell Stem Cell *12*, 368–382.

Yang, Q., Hua, J., Wang, L., Xu, B., Zhang, H., Ye, N., Zhang, Z., Yu, D., Cooke, H.J., Zhang, Y., et al. (2013). MicroRNA and piRNA profiles in normal human testis detected by next generation sequencing. PLoS One *8*, e66809.

Ying, Q.L., Nichols, J., Chambers, I., and Smith, A. (2003). BMP induction of Id proteins suppresses differentiation and sustains embryonic stem cell self-renewal in collaboration with STAT3. Cell *115*, 281–292.

Ying, Q.L., Wray, J., Nichols, J., Batlle-Morera, L., Doble, B., Woodgett, J., Cohen, P., and Smith, A. (2008). The ground state of embryonic stem cell self-renewal. Nature *453*, 519–523.

Zhang, W.J., and Wu, J.Y. (1996). Functional properties of p54, a novel SR protein active in constitutive and alternative splicing. Mol. Cell. Biol. *16*, 5400–5408.

Stem Cell Reports, Volume *9*

Supplemental Information

Small RNA Sequencing Reveals *Dlk1-Dio3* Locus-Embedded MicroRNAs as Major Drivers of Ground-State Pluripotency

Sharif Moradi, Ali Sharifi-Zarchi, Amirhossein Ahmadi, Sepideh Mollamohammadi, Alexander Stubenvoll, Stefan Günther, Ghasem Hosseini Salekdeh, Sassan Asgari, Thomas Braun, and Hossein Baharvand

Supplemental Information

Small RNA Sequencing Reveals *Dlk1-Dio3* Locus-Embedded MicroRNAs as Major Drivers of Ground State Pluripotency

Sharif Moradi, Ali Sharifi-Zarchi, Amirhossein Ahmadi, Sepideh Mollamohammadi, Alexander Stubenvoll, Stefan Günther, Ghasem Hosseini Salekdeh, Sassan Asgari, Thomas Braun, Hossein Baharvand

INVENTORY OF SUPPLEMENTARY INFORMATION

I. Supplementary Data

Figure S1, related to Figures 1 and 3

Figure S2, related to Figure 3

Figure S3, related to Figure 3

Figure S4, related to Figures 4 and 5

Figure S5, related to Figure 4

Figure S6, related to Figures 5, 6, and 7

Table S1, related to Figure 3

Table S2, related to Figure 3

Table S3, related to Figure 3

Table S4, related to Figure 3

Table S5, related to Figure 3

Table S6, related to Figure 3

Table S7, related to Figures 3 and 5

Table S8, related to Figure 5

II. Supplemental Experimental Procedure

III. Supplemental References

I. Supplemental Data

A

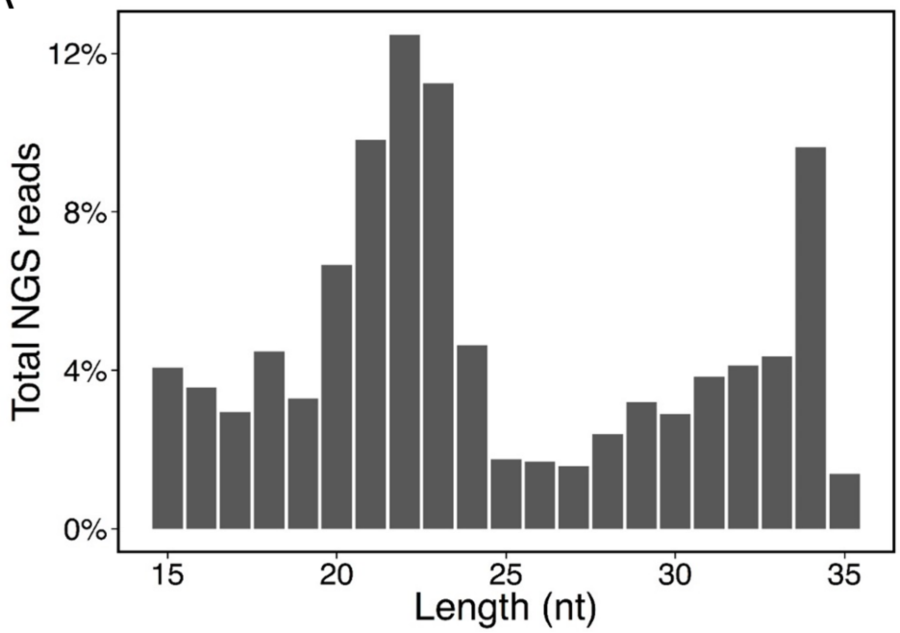

B Top 20: with miR-290 cluster

Serum	2i	R2i
mmu-miR-295-3p	mmu-miR-295-3p	mmu-miR-295-3p
mmu-miR-92a-3p	mmu-miR-182-5p	mmu-miR-182-5p
mmu-miR-182-5p	mmu-miR-183-5p	mmu-miR-92a-3p
mmu-miR-183-5p	mmu-miR-92a-3p	mmu-miR-183-5p
mmu-miR-292a-5p	mmu-miR-16-5p	mmu-miR-16-5p
mmu-miR-292a-3p	mmu-miR-26a-5p	mmu-miR-292a-3p
mmu-miR-291a-5p	mmu-miR-291a-5p	mmu-miR-292a-5p
mmu-miR-294-3p	mmu-miR-292a-3p	mmu-miR-291a-5p
mmu-miR-16-5p	mmu-miR-293-3p	mmu-miR-26a-5p
mmu-miR-26a-5p	mmu-miR-27b-3p	mmu-miR-293-3p
mmu-miR-293-3p	mmu-miR-292a-5p	mmu-miR-294-3p
mmu-miR-30e-5p	mmu-miR-294-3p	mmu-miR-30e-5p
mmu-miR-191-5p	mmu-miR-93-5p	mmu-miR-191-5p
mmu-miR-22-3p	mmu-miR-191-5p	mmu-miR-27b-3p
mmu-miR-21a-5p	mmu-miR-127-3p	mmu-miR-127-3p
mmu-miR-27b-3p	mmu-miR-30e-5p	mmu-miR-93-5p
mmu-miR-93-5p	mmu-miR-291a-3p	mmu-miR-291a-3p
mmu-miR-181c-5p	mmu-miR-148a-3p	mmu-miR-148a-3p
mmu-miR-291a-3p	mmu-miR-186-5p	mmu-miR-293-5p
mmu-miR-293-5p	mmu-miR-293-5p	mmu-miR-25-3p

C Top 20: without miR-290 cluster

Serum	2i	R2i
mmu-miR-92a-3p	mmu-miR-182-5p	mmu-miR-182-5p
mmu-miR-182-5p	mmu-miR-183-5p	mmu-miR-92a-3p
mmu-miR-183-5p	mmu-miR-92a-3p	mmu-miR-183-5p
mmu-miR-16-5p	mmu-miR-16-5p	mmu-miR-16-5p
mmu-miR-26a-5p	mmu-miR-26a-5p	mmu-miR-26a-5p
mmu-miR-30e-5p	mmu-miR-27b-3p	mmu-miR-30e-5p
mmu-miR-191-5p	mmu-miR-93-5p	mmu-miR-191-5p
mmu-miR-22-3p	mmu-miR-191-5p	mmu-miR-27b-3p
mmu-miR-21a-5p	mmu-miR-127-3p	mmu-miR-127-3p
mmu-miR-27b-3p	mmu-miR-30e-5p	mmu-miR-93-5p
mmu-miR-93-5p	mmu-miR-148a-3p	mmu-miR-148a-3p
mmu-miR-181c-5p	mmu-miR-186-5p	mmu-miR-25-3p
mmu-miR-148a-3p	mmu-miR-181c-5p	mmu-miR-541-5p
mmu-miR-25-3p	mmu-miR-25-3p	mmu-miR-302d-3p
mmu-miR-5099	mmu-miR-541-5p	mmu-miR-186-5p
mmu-miR-186-5p	mmu-miR-30d-5p	mmu-miR-130a-3p
mmu-miR-130a-3p	mmu-miR-22-3p	mmu-miR-5099
mmu-miR-7a-5p	mmu-miR-298-5p	mmu-miR-22-3p
mmu-miR-30a-5p	mmu-miR-130a-3p	mmu-miR-30d-5p
mmu-miR-26b-5p	mmu-miR-30c-5p	mmu-miR-30a-5p

Figure S1: Assessment of miRNA expression in serum, 2i, and R2i cultures, related to Figures 1 and 3

(A) Size distribution of small RNA sequences identified by small RNA sequencing

(B) Top 20 abundantly expressed miRNAs in three ESC culture conditions.

(C) Top 20 abundantly expressed miRNAs in three ESC culture conditions excluding the miR-290~295 cluster.

Small RNA sequencing data were obtained for two ESC lines (RB18 and EB20) each time using pools of three independently grown cultures.

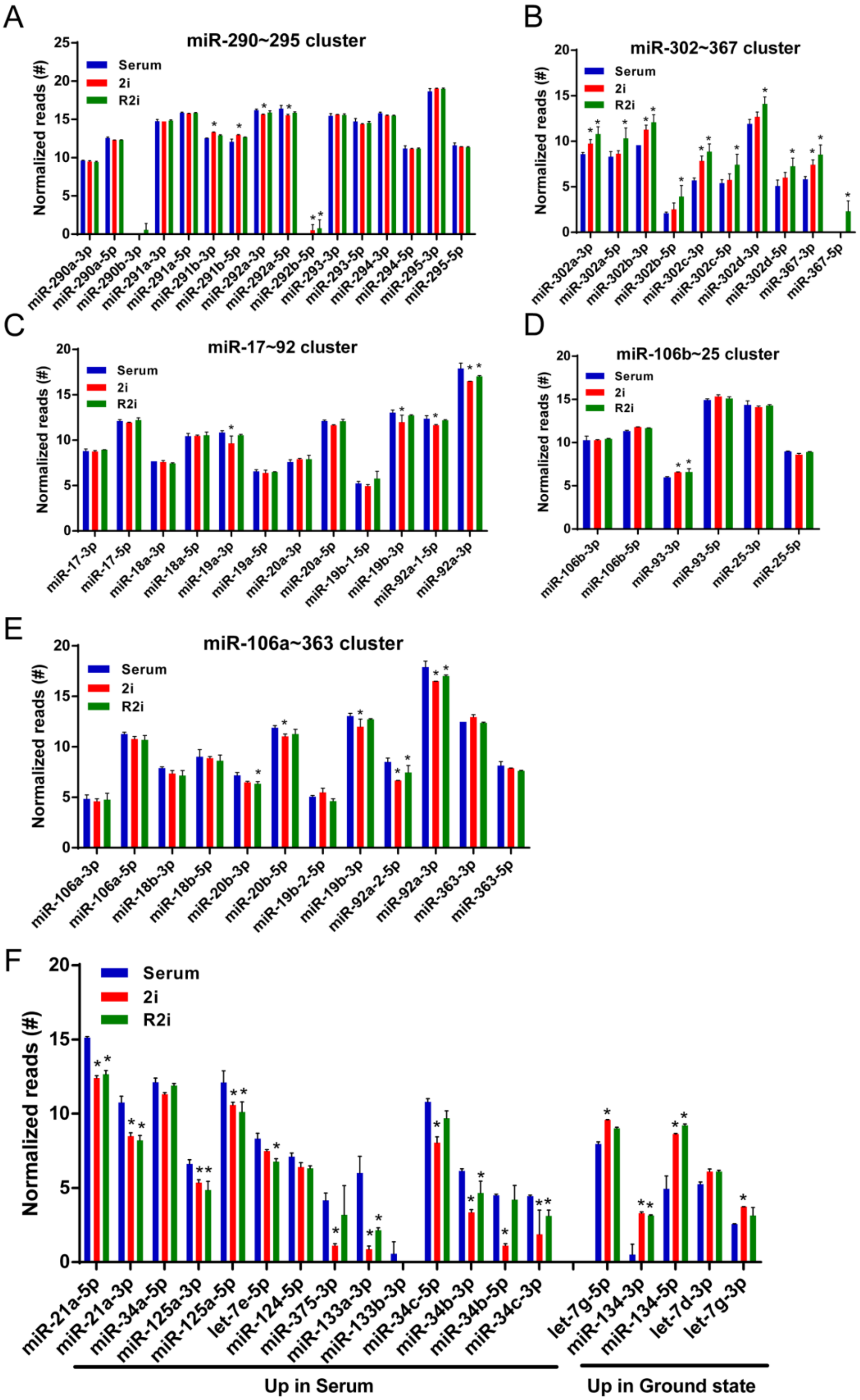
A
miR-290~295 cluster
B
miR-302~367 cluster
C
miR-17~92 cluster
D
miR-106b~25 cluster
E
miR-106a~363 cluster
F
Serum
2i
R2i
Normalized reads (#)
Up in Serum
Up in Ground state

Figure S2: Expression status of miRNAs associated with pluripotency and differentiation, related to Figure 3

(A) Bar plot depicting the expression pattern of members of the miR-290~295 cluster.

(B) Bar plot depicting the expression pattern of the miR-302~367 cluster.

(C-E) Bar plots depicting the expression patterns of the miR-17~92 cluster (E), miR-106b~25 cluster (F), and miR-106a-363 cluster (G).

(F) Bar plot depicting the expression pattern of miRNAs associated with differentiation.

We used two different ESC lines (RB18 and RB20). Each cell line was a pool of three independently grown cultures. Data are shown as mean ± SD. *$p<0.05$.

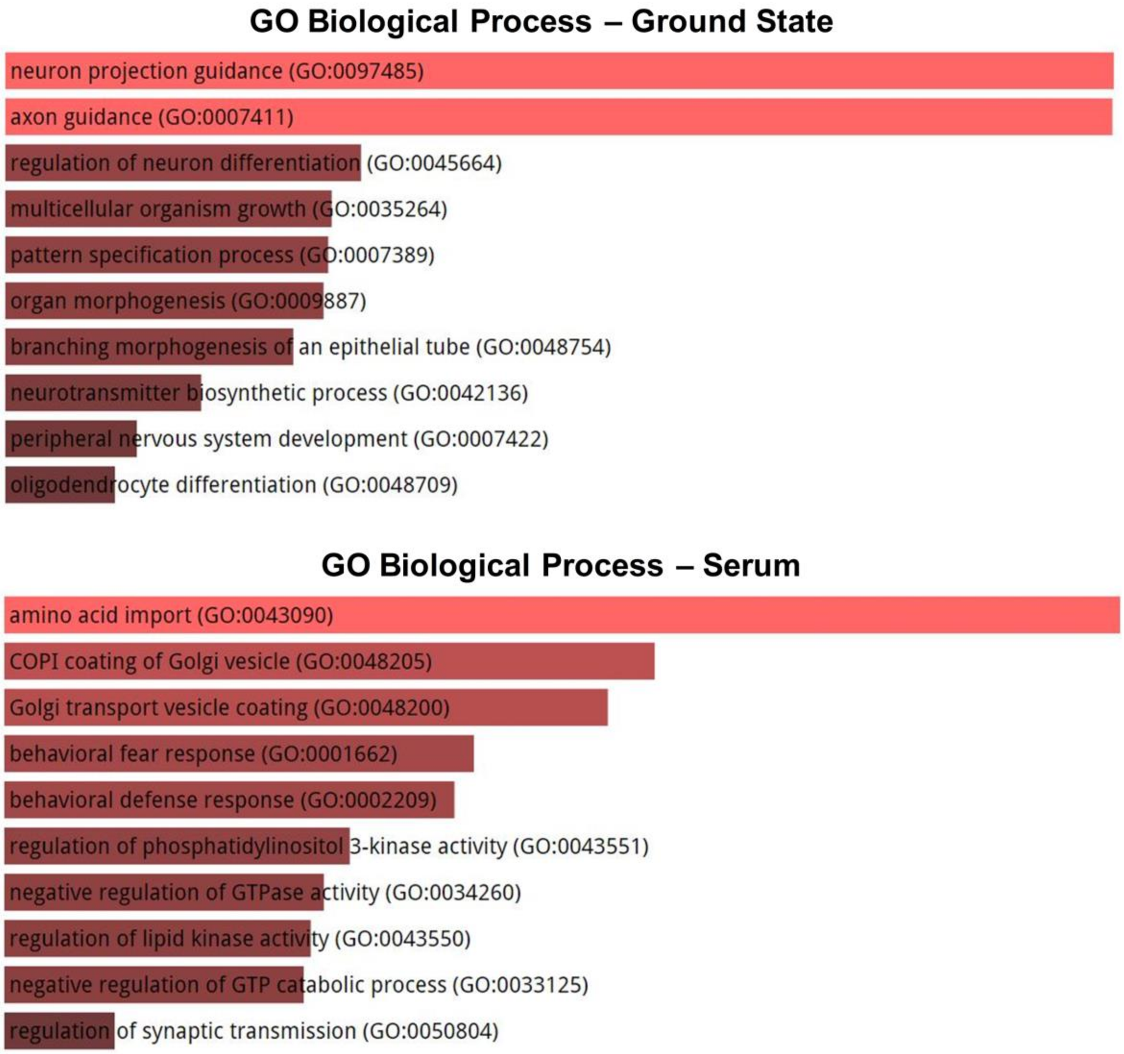

Figure S3: GO analysis of predicted targets of miRNAs associated with serum or ground state using Enrichr, related to Figure 3

A

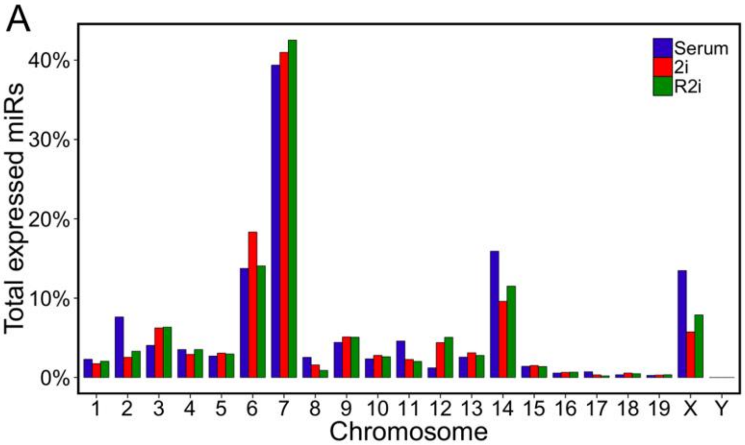

B

Ground state up miRs			
mmu-let-7g-5p	mmu-miR-301a-3p	mmu-miR-376b-5p	mmu-miR-485-5p
mmu-miR-1193-3p	mmu-miR-301a-5p	mmu-miR-376c-3p	mmu-miR-487b-3p
mmu-miR-1197-3p	mmu-miR-301b-3p	mmu-miR-377-3p	mmu-miR-494-3p
mmu-miR-1247-3p	mmu-miR-301b-5p	mmu-miR-379-3p	mmu-miR-495-3p
mmu-miR-127-3p	mmu-miR-302a-3p	mmu-miR-379-5p	mmu-miR-496a-3p
mmu-miR-127-5p	mmu-miR-302b-3p	mmu-miR-380-3p	mmu-miR-499-5p
mmu-miR-134-5p	mmu-miR-302c-3p	mmu-miR-381-3p	mmu-miR-505-3p
mmu-miR-136-3p	mmu-miR-3072-3p	mmu-miR-382-3p	mmu-miR-539-5p
mmu-miR-136-5p	mmu-miR-323-3p	mmu-miR-382-5p	mmu-miR-540-3p
mmu-miR-147-3p	mmu-miR-324-3p	mmu-miR-409-3p	mmu-miR-540-5p
mmu-miR-147-5p	mmu-miR-324-5p	mmu-miR-409-5p	mmu-miR-541-3p
mmu-miR-154-3p	mmu-miR-329-3p	mmu-miR-410-3p	mmu-miR-541-5p
mmu-miR-154-5p	mmu-miR-329-5p	mmu-miR-411-3p	mmu-miR-543-3p
mmu-miR-15a-3p	mmu-miR-337-3p	mmu-miR-411-5p	mmu-miR-665-3p
mmu-miR-15a-5p	mmu-miR-337-5p	mmu-miR-412-3p	mmu-miR-666-3p
mmu-miR-16-1-3p	mmu-miR-341-3p	mmu-miR-412-5p	mmu-miR-666-5p
mmu-miR-184-3p	mmu-miR-341-5p	mmu-miR-431-3p	mmu-miR-667-3p
mmu-miR-187-3p	mmu-miR-367-3p	mmu-miR-431-5p	mmu-miR-668-3p
mmu-miR-1981-5p	mmu-miR-369-3p	mmu-miR-433-3p	mmu-miR-673-3p
mmu-miR-199a-3p	mmu-miR-369-5p	mmu-miR-433-5p	mmu-miR-673-5p
mmu-miR-218-5p	mmu-miR-370-3p	mmu-miR-434-3p	mmu-miR-679-5p
mmu-miR-299a-3p	mmu-miR-370-5p	mmu-miR-434-5p	mmu-miR-743b-3p
mmu-miR-299a-5p	mmu-miR-376a-3p	mmu-miR-451a	mmu-miR-758-3p
mmu-miR-300-3p	mmu-miR-376a-5p	mmu-miR-470-5p	mmu-miR-871-3p
mmu-miR-300-5p	mmu-miR-376b-3p	mmu-miR-485-3p	mmu-miR-874-3p
mmu-miR-98-5p			
72 out of 101 ground state up miRs are embedded in Dlk-1-Dio3 locus			

C

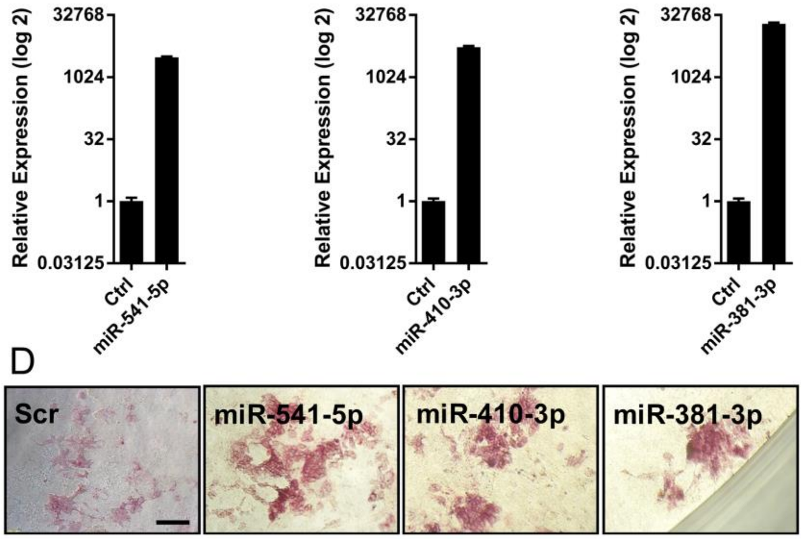

Figure S4: Expression of miRNA genes relative to chromosomal location and functional analysis of ground state-associated miRNAs, related to Figures 4 and 5

(A) Expression of miRNA genes relative to chromosomal location in (i) serum, (ii) 2i, and (iii) R2i cultures. Y-axis indicates the total read numbers of miRNAs expressed from different chromosomes. Small RNA sequencing data were obtained for two ESC lines (RB18 and EB20) each time using pools of three independently grown cultures.

(B) The majority of ground state up miRNAs are embedded in the *Dlk1-Dio3* locus. Ground state up miRNAs that are embedded in *Dlk1-Dio3* locus are shown in red font.

(C) qRT-PCR analysis indicating expression levels of miRNAs delivered into MEFs 1 day after transfection. snoRNA202 was used as an internal normalization control. Data are shown as mean ± SD, n=3.

(D) AP staining of LIF-deprived ESCs treated with miRNA mimics 5 days after transfection. Scale bar: 50 μm.

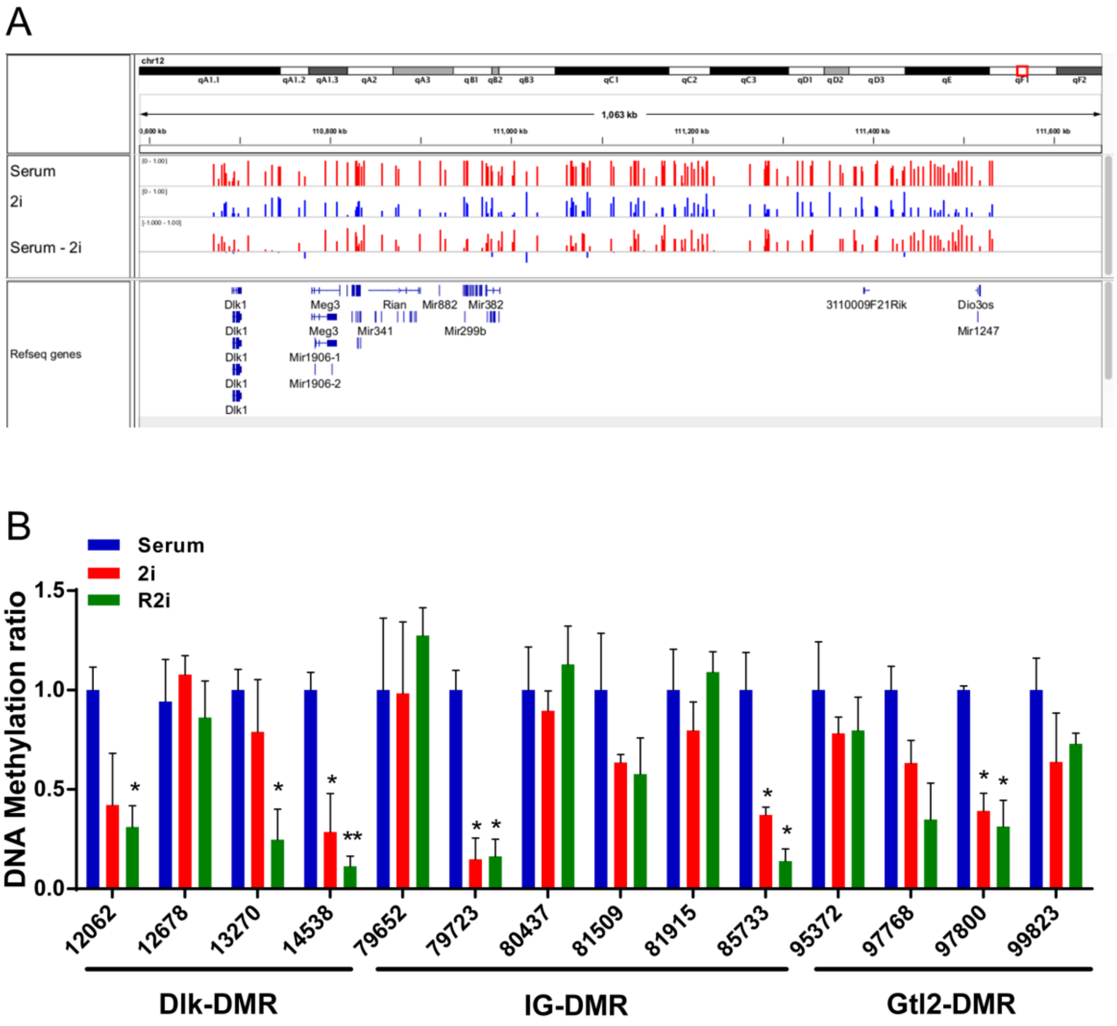

Figure S5: DNA methylation analysis of the *Dlk1-Dio3* locus, related to Figure 4

(A) IGV visualization of the methylation level of CpG dinucleotides at the *Dlk1-Dio3* locus for serum and 2i ESCs.

(B) Bar plots indicating the ratio of DNA methylation of the three DMRs at the *Dlk1-Dio3* locus in serum, 2i, and R2i cells as determined by qPCR. The X-axis numbers refer to the nucleotide positions in the *Dlk1-Dio3* locus corresponding to the site of enzymatic digestion. Data are shown as mean ± SD, n=3. *p<0.05.

A

miRNA	miR-541-5p	miR-410-3p	miR-381-3p
Putative gene targets	P4ha1<br>Prkag1<br>Acat1<br>Cpt1a<br>Myo1b<br>Ero1l<br>Mrpl15<br>Sbno1<br>Cdv3<br>Map4	Myl12b<br>Cbx3<br>Pcbp2<br>Srsf11	Map4<br>Rbm26<br>Idh2<br>Srsf1<br>Pura

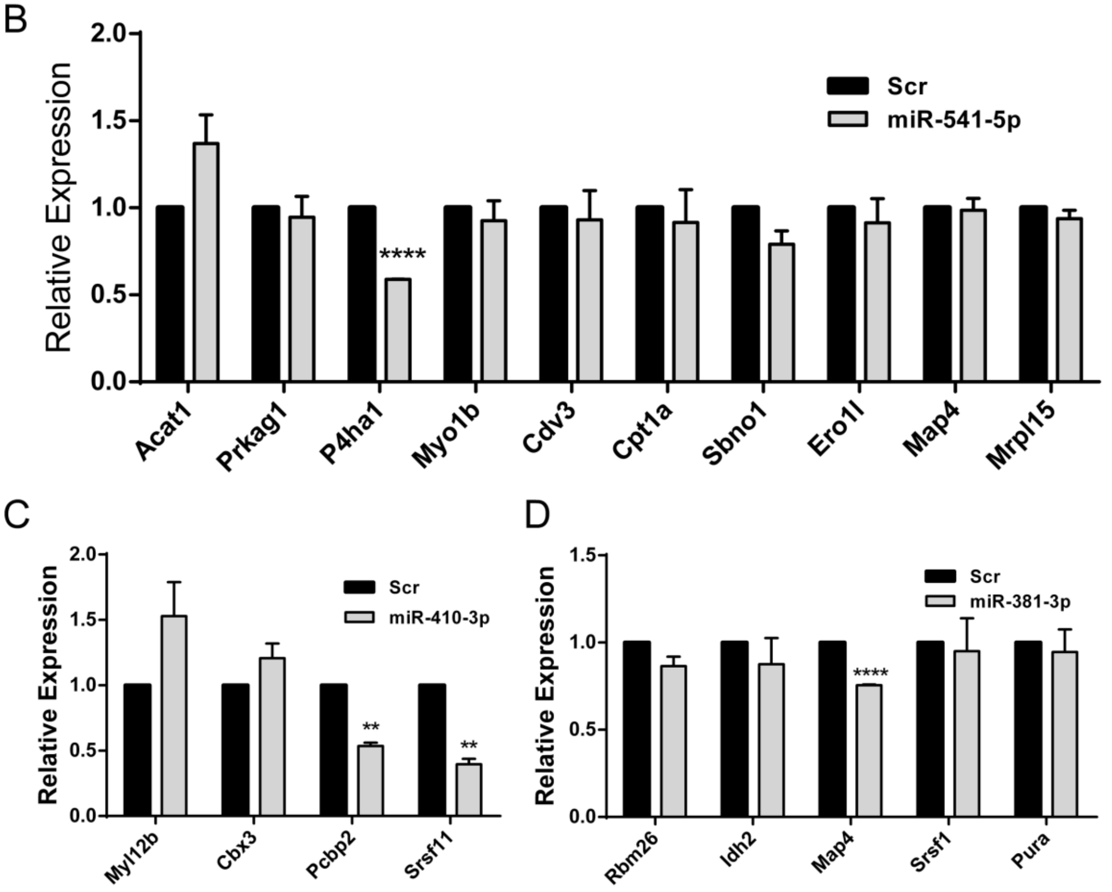

Figure S6: Putative gene targets of miR-541-5p, miR-410-3p, and miR-381-3p, related to Figures 5, 6, and 7

(A) List of genes that are both predicted to be targeted by the candidate miRNAs and downregulated at the protein level.

(B) Bar plot indicating expression of genes that are potentially regulated by miR-541-5p. Data are shown as mean ± SD, n=3. ****p<0.0001

(C) Bar plot indicating the expression of genes that are potentially regulated by miR-410-5p. Data are shown as mean ± SD, n=3. **p<0.01

(D) Bar plot indicating the expression of genes that are potentially regulated by miR-381-5p. Data are shown as mean ± SD, n=3. ****p<0.0001

Table S1. List of 2i-associated miRNAs (versus serum), related to Figure 3

miRNA ID	Log2 fold change	P value	Adjusted P value
mmu-let-7g-5p	1.60052	8.23E-08	5.08E-07
mmu-miR-1193-3p	4.177641	2.62E-08	1.68E-07
mmu-miR-1197-3p	5.020111	5.88E-10	4.37E-09
mmu-miR-127-3p	3.583421	3.93E-25	8.99E-24
mmu-miR-127-5p	4.201075	1.31E-10	1.06E-09
mmu-miR-134-5p	3.515577	3.96E-19	6.11E-18
mmu-miR-136-3p	3.884938	3.00E-31	2.05E-29
mmu-miR-136-5p	4.061845	4.40E-27	1.36E-25
mmu-miR-147-3p	1.535454	8.18E-06	3.85E-05
mmu-miR-147-5p	1.60728	2.46E-05	0.000105
mmu-miR-154-3p	3.66622	5.04E-07	2.83E-06
mmu-miR-154-5p	2.991621	0.000712	0.002363
mmu-miR-15a-3p	1.537571	0.002305	0.006524
mmu-miR-15a-5p	1.510485	6.02E-07	3.32E-06
mmu-miR-184-3p	2.532587	1.07E-08	7.10E-08
mmu-miR-199a-3p	1.23427	0.001959	0.005858
mmu-miR-218-5p	1.336842	0.00029	0.001045
mmu-miR-299a-3p	2.266181	2.80E-05	0.000119
mmu-miR-299a-5p	2.123809	0.002128	0.006253
mmu-miR-300-3p	4.318177	9.93E-34	1.02E-31
mmu-miR-301a-3p	1.41271	1.50E-07	8.88E-07
mmu-miR-301a-5p	1.8322	1.42E-05	6.39E-05
mmu-miR-301b-3p	1.367345	3.86E-06	1.89E-05
mmu-miR-301b-5p	2.461023	0.000719	0.002374
mmu-miR-302a-3p	1.087048	0.00168	0.005107
mmu-miR-302b-3p	1.661023	2.77E-07	1.61E-06
mmu-miR-302c-3p	2.050203	4.35E-07	2.46E-06
mmu-miR-3085-3p	2.79865	5.87E-05	0.00024
mmu-miR-323-3p	4.547108	1.65E-22	3.18E-21
mmu-miR-324-5p	1.766364	8.94E-08	5.46E-07
mmu-miR-329-3p	3.722546	9.44E-11	7.87E-10
mmu-miR-329-5p	3.579605	4.23E-11	3.63E-10
mmu-miR-337-3p	4.599271	1.73E-08	1.13E-07
mmu-miR-337-5p	3.924626	3.28E-19	5.18E-18
mmu-miR-341-3p	3.656685	6.23E-19	9.38E-18
mmu-miR-341-5p	2.958018	0.001512	0.004689
mmu-miR-367-3p	1.516567	0.000476	0.001631
mmu-miR-369-3p	4.156381	3.05E-26	7.53E-25
mmu-miR-369-5p	4.914904	1.02E-09	7.23E-09
mmu-miR-370-3p	3.742272	1.99E-18	2.73E-17
mmu-miR-370-5p	2.679494	0.00211	0.00623
mmu-miR-376a-3p	4.580739	1.14E-19	1.89E-18
mmu-miR-376a-5p	3.429957	1.52E-05	6.79E-05
mmu-miR-376b-3p	4.023984	2.28E-25	5.40E-24
mmu-miR-376b-5p	4.44467	5.64E-12	5.27E-11
mmu-miR-376c-3p	3.50518	1.27E-17	1.63E-16
mmu-miR-377-3p	5.239542	2.05E-14	2.18E-13
mmu-miR-379-3p	3.098211	2.40E-10	1.90E-09
mmu-miR-379-5p	3.211695	1.81E-18	2.56E-17
mmu-miR-380-3p	4.617464	9.99E-38	2.05E-35
mmu-miR-381-3p	3.087566	1.07E-15	1.22E-14
mmu-miR-382-3p	3.47989	4.67E-08	2.97E-07
mmu-miR-382-5p	4.122065	2.23E-14	2.34E-13
mmu-miR-409-3p	3.982859	1.75E-30	1.08E-28
mmu-miR-409-5p	3.59994	2.13E-11	1.97E-10
mmu-miR-410-3p	3.94237	2.77E-28	1.01E-26

mmu-miR-411-3p	3.736336	2.36E-11	2.14E-10
mmu-miR-411-5p	3.679751	1.41E-22	2.81E-21
mmu-miR-412-3p	3.425334	0.003376	0.009342
mmu-miR-412-5p	3.58605	8.97E-07	4.77E-06
mmu-miR-431-3p	5.073442	3.44E-17	4.24E-16
mmu-miR-431-5p	4.681326	2.70E-36	4.17E-34
mmu-miR-433-3p	4.323864	7.97E-18	1.07E-16
mmu-miR-434-3p	3.627581	2.45E-30	1.26E-28
mmu-miR-434-5p	3.652803	2.44E-29	1.07E-27
mmu-miR-451a	3.084706	0.000384	0.001347
mmu-miR-485-3p	3.173984	1.42E-14	1.54E-13
mmu-miR-485-5p	3.455305	5.24E-05	0.000218
mmu-miR-487b-3p	4.388037	8.64E-20	1.48E-18
mmu-miR-493-3p	3.385705	0.00217	0.006342
mmu-miR-494-3p	3.652028	2.64E-07	1.55E-06
mmu-miR-495-3p	4.388145	1.71E-28	7.03E-27
mmu-miR-496a-3p	4.051567	3.33E-12	3.21E-11
mmu-miR-499-5p	3.1575	5.88E-15	6.60E-14
mmu-miR-505-3p	2.738654	0.000355	0.00126
mmu-miR-539-5p	4.224067	7.66E-05	0.000305
mmu-miR-540-3p	3.293543	4.12E-17	4.99E-16
mmu-miR-540-5p	3.734214	5.37E-05	0.000222
mmu-miR-541-5p	3.620427	3.74E-34	4.62E-32
mmu-miR-543-3p	4.300728	2.78E-31	2.05E-29
mmu-miR-665-3p	4.217966	2.00E-28	7.72E-27
mmu-miR-666-3p	5.317512	2.55E-09	1.77E-08
mmu-miR-666-5p	5.06793	1.72E-26	5.04E-25
mmu-miR-667-3p	3.87041	1.19E-07	7.22E-07
mmu-miR-668-3p	4.3836	7.17E-07	3.85E-06
mmu-miR-673-3p	3.94442	3.60E-07	2.07E-06
mmu-miR-673-5p	3.728312	1.53E-19	2.49E-18
mmu-miR-679-5p	2.917934	5.74E-07	3.19E-06
mmu-miR-743b-3p	1.971061	0.000579	0.001942
mmu-miR-758-3p	4.209685	2.20E-30	1.23E-28
mmu-miR-871-3p	1.104186	0.002272	0.006461

Table S2. List of serum-associated miRNAs (versus 2i), related to Figure 3

miRNA ID	Log2 fold change	P value	Adjusted P value
mmu-miR-10a-5p	3.87726	1.98E-31	1.74E-29
mmu-miR-125a-5p	1.57999	5.80E-05	0.000239
mmu-miR-133a-3p	5.43483	6.30E-10	4.63E-09
mmu-miR-155-5p	1.60141	2.01E-06	1.01E-05
mmu-miR-181c-3p	1.39042	8.04E-05	0.000318
mmu-miR-181d-5p	1.24856	1.92E-05	8.36E-05
mmu-miR-1a-3p	6.16233	9.40E-10	6.74E-09
mmu-miR-200a-3p	1.16794	1.17E-05	5.38E-05
mmu-miR-200a-5p	1.64397	0.0001	0.000388
mmu-miR-200b-3p	1.75556	3.51E-10	2.70E-09
mmu-miR-203-3p	4.27738	2.95E-26	7.53E-25
mmu-miR-208b-3p	3.94806	2.92E-11	2.54E-10
mmu-miR-21a-3p	2.23079	4.51E-12	4.28E-11
mmu-miR-21a-5p	2.68639	1.95E-24	4.29E-23
mmu-miR-22-3p	1.58509	4.32E-07	2.46E-06
mmu-miR-23a-3p	1.46251	1.28E-05	5.81E-05
mmu-miR-24-2-5p	1.38923	0.000363	0.001279
mmu-miR-27a-3p	1.4175	1.87E-06	9.56E-06
mmu-miR-27a-5p	1.91032	0.000226	0.000829
mmu-miR-28a-3p	1.95939	3.01E-09	2.06E-08
mmu-miR-28a-5p	1.53412	5.09E-06	2.45E-05
mmu-miR-297a-3p	2.92385	9.40E-07	4.96E-06
mmu-miR-297a-5p	3.03081	6.31E-11	5.33E-10
mmu-miR-297b-3p	3.13206	6.90E-06	3.29E-05
mmu-miR-297b-5p	3.17505	1.62E-05	7.18E-05
mmu-miR-297c-3p	2.39433	0.000852	0.002752
mmu-miR-335-3p	1.73001	3.21E-09	2.18E-08
mmu-miR-34b-3p	2.73207	6.94E-06	3.29E-05
mmu-miR-34c-5p	2.72307	8.57E-17	1.02E-15
mmu-miR-362-3p	1.86082	6.34E-05	0.000258
mmu-miR-374b-3p	1.75378	0.000606	0.002022
mmu-miR-374b-5p	1.48947	1.95E-06	9.86E-06
mmu-miR-421-3p	1.59994	9.82E-07	5.13E-06
mmu-miR-466a-3p	2.4662	1.21E-05	5.55E-05
mmu-miR-466a-5p	2.96415	3.32E-05	0.00014
mmu-miR-466b-3p	2.90333	8.45E-18	1.11E-16
mmu-miR-466b-5p	2.70187	1.34E-14	1.47E-13
mmu-miR-466c-3p	2.67511	2.46E-11	2.20E-10
mmu-miR-466c-5p	2.87989	2.30E-17	2.89E-16
mmu-miR-466d-3p	3.48829	1.73E-06	8.92E-06
mmu-miR-466d-5p	3.25446	4.67E-14	4.72E-13
mmu-miR-466e-3p	2.47321	1.04E-05	4.84E-05
mmu-miR-466e-5p	2.3323	0.000535	0.001805
mmu-miR-466f	2.55241	0.000511	0.001744
mmu-miR-466f-5p	3.34727	1.71E-10	1.37E-09
mmu-miR-466h-3p	3.56794	0.000868	0.00279
mmu-miR-466h-5p	2.87567	2.23E-13	2.21E-12
mmu-miR-466n-3p	2.93058	3.55E-10	2.71E-09
mmu-miR-466n-5p	3.02528	2.61E-12	2.55E-11
mmu-miR-466o-5p	2.66791	4.64E-05	0.000195
mmu-miR-466p-3p	3.02355	6.31E-07	3.44E-06
mmu-miR-466p-5p	2.90491	0.000295	0.001058
mmu-miR-467a-3p	3.54528	1.81E-29	8.58E-28
mmu-miR-467a-5p	2.6155	9.87E-19	1.45E-17
mmu-miR-467b-5p	2.66652	2.09E-09	1.46E-08
mmu-miR-467c-5p	3.63232	4.89E-39	1.51E-36

mmu-miR-467d-3p	3.21852	5.72E-10	4.31E-09
mmu-miR-467d-5p	2.98514	2.25E-20	3.97E-19
mmu-miR-467e-3p	2.97482	6.90E-07	3.74E-06
mmu-miR-467e-5p	3.16111	2.84E-26	7.53E-25
mmu-miR-483-3p	4.62733	3.29E-10	2.57E-09
mmu-miR-483-5p	4.67976	6.90E-05	0.000278
mmu-miR-532-3p	1.45958	0.00263	0.007376
mmu-miR-532-5p	1.50469	5.91E-08	3.72E-07
mmu-miR-669a-3-3p	3.02171	1.83E-05	8.02E-05
mmu-miR-669a-3p	3.08277	1.80E-26	5.04E-25
mmu-miR-669a-5p	2.64805	3.30E-22	6.17E-21
mmu-miR-669b-5p	3.00143	1.83E-18	2.56E-17
mmu-miR-669c-5p	3.32342	1.02E-27	3.32E-26
mmu-miR-669d-5p	3.4231	3.75E-22	6.81E-21
mmu-miR-669e-3p	2.58227	0.002179	0.006342
mmu-miR-669e-5p	3.66885	2.05E-23	4.36E-22
mmu-miR-669f-3p	2.20867	9.07E-05	0.000356
mmu-miR-669f-5p	2.71234	2.67E-11	2.36E-10
mmu-miR-669h-5p	3.24075	6.30E-09	4.23E-08
mmu-miR-669l-3p	3.5162	0.000971	0.003088
mmu-miR-669l-5p	3.57413	8.02E-28	2.75E-26
mmu-miR-669m-5p	3.02974	3.38E-23	6.95E-22
mmu-miR-669o-3p	3.07816	1.31E-10	1.06E-09
mmu-miR-669o-5p	2.48472	4.53E-14	4.66E-13
mmu-miR-669p-3p	2.5773	0.001892	0.005696
mmu-miR-669p-5p	2.62983	5.78E-16	6.73E-15
mmu-miR-674-3p	1.72256	1.16E-06	6.00E-06
mmu-miR-674-5p	1.53656	0.000121	0.000459
mmu-miR-675-3p	4.28965	0.000343	0.001222
mmu-miR-6944-3p	3.30547	6.42E-10	4.66E-09
mmu-miR-92a-2-5p	1.85015	4.20E-06	2.04E-05
mmu-miR-99b-5p	1.04178	0.002262	0.00646

Table S3. List of R2i-associated miRNAs (versus serum), related to Figure 3

miRNA ID	Log2 Fold Change	P value	Adjusted p value
mmu-let-7g-5p	1.026505	0.000639	0.002435
mmu-miR-1193-3p	3.682127	1.18E-06	6.81E-06
mmu-miR-1197-3p	4.557951	2.29E-08	1.63E-07
mmu-miR-1247-3p	2.023308	0.002368	0.008161
mmu-miR-127-3p	3.660928	3.71E-26	2.00E-24
mmu-miR-127-5p	4.250293	9.30E-11	9.25E-10
mmu-miR-134-5p	4.09155	2.44E-25	1.08E-23
mmu-miR-136-3p	3.962237	2.14E-32	4.39E-30
mmu-miR-136-5p	3.563506	4.94E-21	1.53E-19
mmu-miR-147-3p	1.469419	2.21E-05	0.000107
mmu-miR-147-5p	1.87855	9.10E-07	5.35E-06
mmu-miR-153-3p	2.129974	5.11E-06	2.72E-05
mmu-miR-154-3p	3.47848	2.18E-06	1.22E-05
mmu-miR-154-5p	3.404436	0.00012	0.000529
mmu-miR-15a-5p	1.125982	0.000207	0.000882
mmu-miR-184-3p	1.974559	1.02E-05	5.27E-05
mmu-miR-187-3p	1.262837	0.00274	0.009379
mmu-miR-218-5p	1.115704	0.002751	0.009379
mmu-miR-299a-3p	2.915455	6.74E-08	4.57E-07
mmu-miR-299a-5p	2.502315	0.000304	0.001259
mmu-miR-300-3p	4.095046	2.00E-30	2.46E-28
mmu-miR-300-5p	3.300759	0.000978	0.003553
mmu-miR-301a-3p	1.169306	1.39E-05	7.03E-05
mmu-miR-301a-5p	1.428146	0.000847	0.00315
mmu-miR-301b-3p	1.056701	0.000367	0.001499
mmu-miR-302a-3p	2.172296	3.26E-10	2.96E-09
mmu-miR-302a-5p	2.010583	2.38E-06	1.32E-05
mmu-miR-302b-3p	2.494541	1.17E-14	1.68E-13
mmu-miR-302c-3p	3.070571	3.19E-14	4.38E-13
mmu-miR-302c-5p	1.992109	6.53E-05	0.000301
mmu-miR-302d-3p	2.123171	1.07E-08	7.87E-08
mmu-miR-302d-5p	2.053181	6.84E-05	0.000313
mmu-miR-3072-3p	3.127403	0.00189	0.006625
mmu-miR-323-3p	4.322572	2.48E-20	6.64E-19
mmu-miR-324-5p	1.074954	0.001285	0.004584
mmu-miR-329-3p	3.510127	1.31E-09	1.08E-08
mmu-miR-329-5p	3.44769	2.83E-10	2.61E-09
mmu-miR-337-3p	4.820792	3.72E-09	2.98E-08
mmu-miR-337-5p	4.003505	8.28E-20	1.89E-18
mmu-miR-341-3p	3.683565	3.93E-19	8.35E-18

mmu-miR-3535	1.127635	0.000149	0.000646
mmu-miR-367-3p	2.672808	6.26E-10	5.37E-09
mmu-miR-369-3p	3.991549	3.37E-24	1.09E-22
mmu-miR-369-5p	4.880154	1.52E-09	1.23E-08
mmu-miR-370-3p	3.634605	2.37E-17	4.29E-16
mmu-miR-376a-3p	4.364697	7.54E-18	1.41E-16
mmu-miR-376a-5p	2.857094	0.000374	0.001519
mmu-miR-376b-3p	3.611718	1.45E-20	4.26E-19
mmu-miR-376b-5p	4.130688	2.00E-10	1.93E-09
mmu-miR-376c-3p	3.589962	2.77E-18	5.51E-17
mmu-miR-377-3p	4.845733	2.00E-12	2.29E-11
mmu-miR-379-3p	3.257469	3.24E-11	3.27E-10
mmu-miR-379-5p	3.872263	3.89E-26	2.00E-24
mmu-miR-380-3p	4.230026	9.08E-32	1.40E-29
mmu-miR-381-3p	3.516498	6.79E-20	1.66E-18
mmu-miR-382-3p	3.336479	2.02E-07	1.31E-06
mmu-miR-382-5p	4.237989	5.05E-15	7.98E-14
mmu-miR-409-3p	3.950542	5.72E-30	5.88E-28
mmu-miR-409-5p	3.403733	3.37E-10	3.01E-09
mmu-miR-410-3p	3.716682	2.78E-25	1.14E-23
mmu-miR-411-3p	3.801019	1.32E-11	1.40E-10
mmu-miR-411-5p	3.831339	2.92E-24	1.06E-22
mmu-miR-412-5p	2.884534	9.87E-05	0.000444
mmu-miR-431-3p	4.841877	1.12E-15	1.82E-14
mmu-miR-431-5p	4.202259	2.05E-29	1.58E-27
mmu-miR-433-3p	3.853132	2.86E-14	4.01E-13
mmu-miR-434-3p	3.584975	1.39E-29	1.23E-27
mmu-miR-434-5p	3.880914	7.24E-33	2.23E-30
mmu-miR-451a	3.925137	5.86E-06	3.09E-05
mmu-miR-470-5p	1.584688	9.93E-05	0.000444
mmu-miR-485-3p	3.212024	9.05E-15	1.36E-13
mmu-miR-485-5p	3.642984	2.15E-05	0.000104
mmu-miR-487b-3p	4.08367	3.53E-17	5.88E-16
mmu-miR-494-3p	4.175452	4.02E-09	3.18E-08
mmu-miR-495-3p	4.045337	3.15E-24	1.08E-22
mmu-miR-496a-3p	3.940805	1.65E-11	1.73E-10
mmu-miR-499-5p	2.932804	5.69E-13	6.75E-12
mmu-miR-5121	1.247815	0.000174	0.000751
mmu-miR-539-5p	4.292943	6.19E-05	0.000287
mmu-miR-540-3p	3.529823	2.44E-19	5.37E-18
mmu-miR-540-5p	3.211803	0.000588	0.002254
mmu-miR-541-5p	3.900606	2.31E-39	1.43E-36

mmu-miR-543-3p	4.055485	8.85E-28	6.07E-26
mmu-miR-665-3p	4.128172	3.84E-27	2.37E-25
mmu-miR-666-3p	4.152546	4.29E-06	2.32E-05
mmu-miR-666-5p	4.418284	2.93E-20	7.53E-19
mmu-miR-667-3p	3.599379	1.04E-06	6.08E-06
mmu-miR-668-3p	3.157516	0.000451	0.001795
mmu-miR-673-3p	4.02305	2.34E-07	1.49E-06
mmu-miR-673-5p	3.770622	7.01E-20	1.66E-18
mmu-miR-677-5p	1.303209	2.06E-05	0.000102
mmu-miR-679-5p	2.899979	8.11E-07	4.81E-06
mmu-miR-758-3p	3.85022	1.74E-25	8.26E-24
mmu-miR-871-3p	1.360755	0.000175	0.000751
mmu-miR-874-3p	2.629872	2.56E-07	1.61E-06

Table S4. List of serum-associated miRNAs (versus R2i), related to Figure 3

miRNA ID	Log2 Fold Change	P value	Adjusted p value
mmu-let-7e-5p	1.52341	3.01E-05	0.000144
mmu-miR-10a-5p	2.15682	2.14E-11	2.20E-10
mmu-miR-125a-3p	1.65385	0.000949	0.003484
mmu-miR-125a-5p	1.9674	5.77E-07	3.49E-06
mmu-miR-125b-1-3p	1.79227	0.000477	0.001888
mmu-miR-133a-3p	3.97152	2.97E-07	1.85E-06
mmu-miR-142a-3p	2.19719	7.19E-08	4.82E-07
mmu-miR-142a-5p	2.43938	6.36E-12	7.01E-11
mmu-miR-146a-5p	2.04349	1.76E-10	1.72E-09
mmu-miR-152-5p	1.53467	0.000979	0.003553
mmu-miR-155-5p	1.48648	1.25E-05	6.38E-05
mmu-miR-181c-3p	2.60936	4.29E-13	5.29E-12
mmu-miR-181c-5p	2.14744	6.17E-15	9.52E-14
mmu-miR-181d-5p	2.4829	2.76E-17	4.87E-16
mmu-miR-1a-3p	2.57797	0.000411	0.001645
mmu-miR-200a-3p	1.05069	8.20E-05	0.000372
mmu-miR-200a-5p	1.5657	0.00026	0.001086
mmu-miR-200b-3p	1.40789	4.94E-07	3.05E-06
mmu-miR-203-3p	3.46253	1.70E-18	3.51E-17
mmu-miR-208b-3p	3.73695	6.26E-10	5.37E-09
mmu-miR-21a-3p	2.51088	1.17E-14	1.68E-13
mmu-miR-21a-5p	2.43637	2.25E-20	6.30E-19
mmu-miR-22-3p	1.50959	1.49E-06	8.49E-06
mmu-miR-23a-3p	1.94247	8.39E-09	6.31E-08
mmu-miR-24-2-5p	2.21037	2.10E-08	1.51E-07
mmu-miR-27a-3p	1.4747	7.51E-07	4.50E-06
mmu-miR-27a-5p	2.53934	2.99E-06	1.63E-05
mmu-miR-28a-3p	1.25755	0.000136	0.000596
mmu-miR-28a-5p	1.23602	0.00025	0.00105
mmu-miR-297a-3p	2.07167	0.0004	0.001613
mmu-miR-297a-5p	2.12757	2.79E-06	1.54E-05
mmu-miR-297b-3p	2.38195	0.000488	0.001916
mmu-miR-297c-3p	2.26393	0.001958	0.006826
mmu-miR-31-5p	1.68844	7.88E-08	5.23E-07
mmu-miR-335-3p	1.1739	5.87E-05	0.000274
mmu-miR-34c-5p	1.08604	0.000826	0.003087
mmu-miR-362-3p	1.58077	0.000761	0.002879
mmu-miR-374b-5p	1.2173	0.000106	0.000469
mmu-miR-421-3p	1.34879	3.90E-05	0.000185
mmu-miR-466a-3p	2.07749	0.000245	0.001034

mmu-miR-466b-3p	2.10253	3.74E-10	3.29E-09
mmu-miR-466b-5p	2.12545	1.31E-09	1.08E-08
mmu-miR-466c-3p	1.78562	6.25E-06	3.27E-05
mmu-miR-466c-5p	2.14839	2.18E-10	2.07E-09
mmu-miR-466d-3p	2.1528	0.001796	0.006333
mmu-miR-466d-5p	2.43989	9.38E-09	6.98E-08
mmu-miR-466e-3p	1.69531	0.002196	0.00761
mmu-miR-466f	2.60037	0.000581	0.00224
mmu-miR-466f-5p	2.15427	1.83E-05	9.08E-05
mmu-miR-466h-5p	2.13618	3.93E-08	2.72E-07
mmu-miR-466n-3p	2.053	7.97E-06	4.13E-05
mmu-miR-466n-5p	2.22737	1.79E-07	1.17E-06
mmu-miR-466p-3p	1.9775	0.000809	0.003044
mmu-miR-466p-5p	2.64913	0.001106	0.003992
mmu-miR-467a-3p	2.63471	3.16E-17	5.42E-16
mmu-miR-467a-5p	1.80709	9.20E-10	7.77E-09
mmu-miR-467b-5p	1.55055	0.000349	0.001435
mmu-miR-467c-5p	2.82503	2.37E-24	9.15E-23
mmu-miR-467d-3p	2.8617	4.48E-08	3.07E-07
mmu-miR-467d-5p	2.28022	1.24E-12	1.45E-11
mmu-miR-467e-3p	2.60333	1.58E-05	7.92E-05
mmu-miR-467e-5p	2.24406	3.47E-14	4.65E-13
mmu-miR-483-3p	3.21729	1.57E-06	8.86E-06
mmu-miR-532-5p	1.59101	1.19E-08	8.62E-08
mmu-miR-669a-3p	2.51499	3.64E-18	7.03E-17
mmu-miR-669a-5p	1.85917	9.65E-12	1.04E-10
mmu-miR-669b-5p	2.13996	2.67E-10	2.50E-09
mmu-miR-669c-5p	2.28157	4.73E-14	6.21E-13
mmu-miR-669d-5p	2.40782	4.06E-12	4.56E-11
mmu-miR-669e-5p	2.62906	2.32E-13	2.92E-12
mmu-miR-669f-3p	1.96794	0.000567	0.002206
mmu-miR-669f-5p	1.83346	4.83E-06	2.59E-05
mmu-miR-669h-5p	2.16641	5.79E-05	0.000273
mmu-miR-669l-5p	2.41132	5.44E-14	7.00E-13
mmu-miR-669m-5p	2.1922	5.20E-13	6.29E-12
mmu-miR-669o-3p	2.37317	5.37E-07	3.28E-06
mmu-miR-669o-5p	1.90913	6.70E-09	5.17E-08
mmu-miR-669p-3p	3.00953	0.000568	0.002206
mmu-miR-669p-5p	1.8738	6.99E-09	5.32E-08
mmu-miR-674-3p	2.11798	5.96E-09	4.66E-08
mmu-miR-674-5p	1.73585	2.09E-05	0.000102
mmu-miR-6944-3p	2.98412	3.18E-08	2.23E-07

mmu-miR-99b-3p	1.5231	0.000867	0.003202
mmu-miR-99b-5p	1.77098	2.16E-07	1.39E-06

Table S5. List of 2i- and R2i-associated miRNAs, related to Figure 3

miRNA ID	Log2 Fold Change	P value	Adjusted p value
**2i-associated miRNAs (versus R2i)**			
mmu-miR-142a-3p	1.92114	2.45E-06	0.000134
mmu-miR-142a-5p	2.62459	1.41E-13	3.48E-11
mmu-miR-146a-5p	1.23012	0.000126	0.004782
mmu-miR-152-3p	1.86974	2.18E-08	2.68E-06
mmu-miR-152-5p	2.44772	8.70E-08	8.57E-06
mmu-miR-181c-5p	1.44296	1.60E-07	1.31E-05
mmu-miR-181d-5p	1.23435	2.67E-05	0.001187
mmu-miR-211-3p	4.5873	2.63E-10	4.32E-08
mmu-miR-211-5p	4.56645	2.23E-39	1.10E-36
**R2i-associated miRNAs (versus 2i)**			
mmu-miR-10a-5p	1.720439	4.07E-07	2.87E-05
mmu-miR-302a-5p	1.776923	2.89E-05	0.001187
mmu-miR-302d-3p	1.412517	0.000141	0.00496
mmu-miR-34c-5p	1.637035	6.20E-07	3.82E-05
mmu-miR-3535	1.325008	8.27E-06	0.000408
mmu-miR-6240	1.335964	0.000265	0.00872

Table S6. List of serum- and ground state-associated miRNAs, related to Figure 3

Ground state-associated miRNAs (versus serum)			Serum-associated miRNAs (versus ground state)		
**miRNA ID**	**Log2 Fold Change**	**Adjusted p value**	**miRNA ID**	**Log2 Fold Change**	**Adjusted p value**
mmu-let-7g-5p	1.346466	0.000227	mmu-miR-10a-5p	2.71438	1.40E-05
mmu-miR-1193-3p	4.223692	1.53E-07	mmu-miR-125a-3p	1.45761	0.001066
mmu-miR-1197-3p	5.213232	2.83E-08	mmu-miR-125a-5p	1.76216	0.000277
mmu-miR-1247-3p	2.047209	0.001109	mmu-miR-133a-3p	4.77846	1.16E-09
mmu-miR-127-3p	3.641083	1.23E-22	mmu-miR-149-5p	1.0969	0.003174
mmu-miR-127-5p	4.411212	1.05E-10	mmu-miR-155-5p	1.55942	6.57E-08
mmu-miR-134-5p	3.863615	9.43E-18	mmu-miR-181a-2-3p	2.07394	0.006915
mmu-miR-136-3p	3.950312	8.35E-31	mmu-miR-181c-3p	1.87785	0.000973
mmu-miR-136-5p	3.868125	1.29E-20	mmu-miR-181d-5p	1.72886	0.001278
mmu-miR-147-3p	1.519911	7.51E-07	mmu-miR-200a-3p	1.11462	3.42E-06
mmu-miR-147-5p	1.769462	3.53E-07	mmu-miR-200a-5p	1.62601	4.12E-05
mmu-miR-154-3p	3.77435	1.04E-06	mmu-miR-200b-3p	1.57816	1.68E-07
mmu-miR-154-5p	3.540456	0.00019	mmu-miR-203-3p	3.85143	2.67E-20
mmu-miR-15a-3p	1.40228	0.003559	mmu-miR-208b-3p	3.93947	8.45E-13
mmu-miR-15a-5p	1.336776	9.95E-05	mmu-miR-21a-3p	2.37758	1.72E-12
mmu-miR-16-1-3p	1.007219	0.009531	mmu-miR-21a-5p	2.56769	2.36E-24
mmu-miR-184-3p	2.2992	3.49E-06	mmu-miR-22-3p	1.55562	3.42E-06

mmu-miR-187-3p	1.175675	0.00399	mmu-miR-23a-3p	1.69033	2.39E-05
mmu-miR-1981-5p	1.555043	0.002766	mmu-miR-24-2-5p	1.74394	0.000958
mmu-miR-199a-3p	1.20635	0.003041	mmu-miR-27a-3p	1.45499	8.59E-07
mmu-miR-218-5p	1.246712	0.000324	mmu-miR-27a-5p	2.20878	3.72E-05
mmu-miR-299a-3p	2.684175	1.88E-06	mmu-miR-28a-3p	1.57158	0.000144
mmu-miR-299a-5p	2.447515	0.000307	mmu-miR-28a-5p	1.38864	6.54E-05
mmu-miR-300-3p	4.242033	1.42E-28	mmu-miR-297a-3p	2.51336	1.56E-05
mmu-miR-300-5p	3.341582	0.004116	mmu-miR-297a-5p	2.55356	9.07E-08
mmu-miR-301a-3p	1.302725	1.28E-06	mmu-miR-297b-3p	2.84516	2.98E-06
mmu-miR-301a-5p	1.672406	5.31E-05	mmu-miR-297b-5p	2.5682	0.000793
mmu-miR-301b-3p	1.227613	0.000166	mmu-miR-297c-3p	2.45532	5.69E-05
mmu-miR-301b-5p	2.362827	0.001111	mmu-miR-335-3p	1.42225	0.000136
mmu-miR-302a-3p	1.729754	0.001358	mmu-miR-34b-3p	1.99459	0.003108
mmu-miR-302b-3p	2.140033	2.14E-06	mmu-miR-362-3p	1.75341	1.11E-05
mmu-miR-302c-3p	2.657353	1.20E-06	mmu-miR-374b-3p	1.68092	0.000199
mmu-miR-3072-3p	3.133091	0.00741	mmu-miR-374b-5p	1.35723	1.36E-05
mmu-miR-323-3p	4.52449	9.71E-22	mmu-miR-421-3p	1.47989	1.38E-05
mmu-miR-324-3p	1.247244	0.004731	mmu-miR-466a-3p	2.33663	9.17E-07
mmu-miR-324-5p	1.466346	0.000442	mmu-miR-466a-5p	2.52089	0.000161
mmu-miR-329-3p	3.736309	2.27E-10	mmu-miR-466b-3p	2.45995	4.45E-09
mmu-miR-329-5p	3.622638	2.37E-12	mmu-miR-466b-5p	2.4044	6.79E-11
mmu-miR-337-3p	5.131488	3.60E-08	mmu-miR-466c-3p	2.18635	2.73E-06
mmu-miR-337-5p	4.033849	2.20E-22	mmu-miR-466c-5p	2.47573	2.17E-09
mmu-miR-341-3p	3.708818	1.16E-16	mmu-miR-466d-3p	2.78809	0.000102
mmu-miR-341-5p	2.819602	0.008834	mmu-miR-466d-5p	2.83411	1.43E-10
mmu-miR-367-3p	2.21524	0.000268	mmu-miR-466e-3p	2.09689	8.33E-05
mmu-miR-369-3p	4.125175	2.00E-24	mmu-miR-466e-5p	2.25502	0.000118
mmu-miR-369-5p	5.316992	6.83E-09	mmu-miR-466f	2.71802	2.78E-05
mmu-miR-370-3p	3.742474	2.76E-17	mmu-miR-466f-5p	2.67411	2.41E-06
mmu-miR-370-5p	2.668108	0.004692	mmu-miR-466h-3p	3.69355	0.001068
mmu-miR-376a-3p	4.589456	6.72E-20	mmu-miR-466h-5p	2.48919	1.83E-09
mmu-miR-376a-5p	3.399585	6.99E-05	mmu-miR-466k	2.34043	0.006553
mmu-miR-376b-3p	3.872204	2.61E-21	mmu-miR-466n-3p	2.46922	9.11E-07
mmu-miR-376b-5p	4.48077	5.51E-11	mmu-miR-466n-5p	2.61288	5.01E-09
mmu-miR-376c-3p	3.603797	3.68E-22	mmu-miR-466o-5p	2.18533	0.000515
mmu-miR-377-3p	5.328743	1.56E-12	mmu-miR-466p-3p	2.46707	0.00017
mmu-miR-379-3p	3.244779	4.25E-11	mmu-miR-466p-5p	2.95751	5.31E-05
mmu-miR-379-5p	3.591239	4.87E-16	mmu-miR-467a-3p	3.01199	2.93E-12
mmu-miR-380-3p	4.474326	2.03E-30	mmu-miR-467a-5p	2.15512	1.34E-07
mmu-miR-381-3p	3.32822	1.86E-13	mmu-miR-467b-5p	2.02111	0.000164
mmu-miR-382-3p	3.570635	6.42E-09	mmu-miR-467c-5p	3.16445	4.75E-16
mmu-miR-382-5p	4.298436	1.94E-15	mmu-miR-467d-3p	3.10989	1.27E-12

mmu-miR-409-3p	3.995605	2.48E-28	mmu-miR-467d-5p	2.60192	6.64E-12
mmu-miR-409-5p	3.609228	2.63E-12	mmu-miR-467e-3p	2.88294	8.68E-09
mmu-miR-410-3p	3.858489	4.31E-23	mmu-miR-467e-5p	2.62303	6.26E-10
mmu-miR-411-3p	3.893429	3.42E-13	mmu-miR-483-3p	3.90738	1.10E-08
mmu-miR-411-5p	3.796325	3.71E-23	mmu-miR-483-5p	4.2486	0.000928
mmu-miR-412-3p	4.300049	0.006055	mmu-miR-532-3p	1.44713	0.000942
mmu-miR-412-5p	3.474198	7.43E-06	mmu-miR-532-5p	1.55719	6.79E-11
mmu-miR-431-3p	5.14734	5.45E-16	mmu-miR-669a-3-3p	2.50769	0.000227
mmu-miR-431-5p	4.49795	8.73E-27	mmu-miR-669a-3p	2.77868	7.00E-17
mmu-miR-433-3p	4.197099	2.10E-15	mmu-miR-669a-5p	2.19413	2.08E-08
mmu-miR-433-5p	2.95281	0.005417	mmu-miR-669b-5p	2.51493	2.68E-09
mmu-miR-434-3p	3.633688	5.23E-33	mmu-miR-669c-5p	2.69534	6.17E-09
mmu-miR-434-5p	3.794216	1.52E-29	mmu-miR-669d-5p	2.82857	9.73E-10
mmu-miR-451a	3.866863	9.67E-05	mmu-miR-669e-3p	2.74839	0.000415
mmu-miR-470-5p	1.359331	0.002239	mmu-miR-669e-5p	3.06581	3.55E-11
mmu-miR-485-3p	3.243292	7.72E-18	mmu-miR-669f-3p	2.15046	6.76E-06
mmu-miR-485-5p	3.886438	2.57E-05	mmu-miR-669f-5p	2.23105	9.94E-07
mmu-miR-487b-3p	4.331313	2.22E-18	mmu-miR-669h-5p	2.67599	1.34E-06
mmu-miR-494-3p	4.105908	1.56E-07	mmu-miR-669l-3p	2.9387	0.006154
mmu-miR-495-3p	4.282929	1.75E-27	mmu-miR-669l-5p	2.86718	3.80E-09
mmu-miR-496a-3p	4.141768	3.78E-13	mmu-miR-669m-5p	2.55116	3.50E-10
mmu-miR-499-5p	3.08594	2.12E-14	mmu-miR-669o-3p	2.73991	1.46E-09
mmu-miR-505-3p	2.56491	0.001681	mmu-miR-669o-5p	2.18384	1.93E-09
mmu-miR-539-5p	5.241534	0.000221	mmu-miR-669p-3p	2.96495	7.74E-05
mmu-miR-540-3p	3.459006	1.18E-19	mmu-miR-669p-5p	2.21086	4.34E-08
mmu-miR-540-5p	3.925899	0.000189	mmu-miR-674-3p	1.9181	1.13E-08
mmu-miR-541-3p	4.375227	0.004831	mmu-miR-674-5p	1.64858	8.56E-06
mmu-miR-541-5p	3.784097	1.12E-33	mmu-miR-675-3p	4.11139	0.001253
mmu-miR-543-3p	4.233375	5.23E-33	mmu-miR-6944-3p	3.22073	2.08E-11
mmu-miR-665-3p	4.229401	8.98E-32	mmu-miR-92a-3p	1.14356	0.004217
mmu-miR-666-3p	5.330333	2.14E-06	mmu-miR-99b-3p	1.37392	0.001109
mmu-miR-666-5p	4.860263	1.56E-20	mmu-miR-99b-5p	1.35383	0.004856
mmu-miR-667-3p	3.98145	1.53E-07			
mmu-miR-668-3p	4.234536	7.74E-05			
mmu-miR-673-3p	4.259766	3.26E-07			
mmu-miR-673-5p	3.790264	5.95E-17			
mmu-miR-679-5p	3.014489	2.14E-08			
mmu-miR-743b-3p	1.798647	0.001278			
mmu-miR-758-3p	4.080903	5.22E-27			
mmu-miR-871-3p	1.247032	0.00108			
mmu-miR-874-3p	2.032867	0.006821			
mmu-miR-98-5p	1.050864	0.005209			

Table S7: List of predicted mRNA targets used for gene ontology and pathway analysis, related to Figures 3 and 5

Table S8. Potential biological pathways inhibited by miR-541-5p, miR-410-3p, and miR-381-3p (DAVID analysis), related to Figure 5

**miR-541-5p targets**	
**GO term**	**P value**
Chordate embryonic development	1.2E-3
Muscle tissue development	9.9E-2
Transcription regulation	3.9E-3
Embryonic limb morphogenesis	5.5E-2
Nucleotide-binding	1.5E-2
Adult locomotor behavior	2.4E-2
Chromatin binding	2.9E-3
Pattern specification process	9.9E-2
**miR-410-3p targets**	
Transcription regulation	1.7E-22
Vasculature development	7.8E-9
Respiratory system development	9.6E-9
Neuron differentiation	6.8E-9
Cell migration	1.0E-6
Wnt signaling pathway	6.5E-7
Sensory organ development	2.3E-7
Chordate embryonic development	2.0E-5
**miR-381-3p targets**	
Transcription regulation	4.9E-26
Pattern specification process	1.1E-8
Cell morphogenesis during differentiation	6.1E-10
Neuron differentiation	1.6E-7
Embryonic morphogenesis	2.7E-7
Nucleoplasm	1.6E-9
Sensory organ development	5.2E-8
Wnt signaling pathway	1.7E-5

II. Supplemental Experimental Procedures

Cell culture

RB18 and RB20 mouse ESC lines were maintained at 37˚C in 5% CO_2, cultivated on gelatinized tissue-culture plates and dishes (Sigma-Aldrich), and passaged every other day. Serum ESCs were cultured in Knockout™ DMEM (Invitrogen) supplemented with 15% ES-qualified fetal bovine serum (HyClone), 0.1 mM β-mercaptoethanol (Sigma-Aldrich), 0.1 mM non-essential amino acids (Invitrogen), 2 mM L-glutamine (Invitrogen), 100 U/ml penicillin, 100 μg/ml streptomycin (Invitrogen), and 1000 U/ml mouse LIF (mLIF, Royan Biotech). 2i and R2i cells were cultured in N2B27 media that consisted of Neurobasal® medium and DMEM/F-12 (both from Invitrogen) at a 1:1 ratio, 1% N2 supplement (Invitrogen), 1% B27 supplement (Invitrogen), 0.1 mM β-mercaptoethanol, 5 mg/ml BSA (Invitrogen), 2 mM L-glutamine, 0.1 mM non-essential amino acids, 100 U/ml penicillin, 100 μg/ml streptomycin, and 1000 U/ml mLIF. Small-molecule chemicals used in 2i and R2i cultures were added at the following concentrations: 1 μM PD0325901 (Stemgent), 10 μM SB431542 (Sigma-Aldrich), and 3 μM CHIR99021 (Stemgent).

For spontaneous differentiation through embryoid body (EB) formation, the ESCs were trypsinized and suspended in serum ESC medium without LIF on non-adherent bacterial petri dishes at 1.5×10^5 cells/ml. Five days after induction of EB formation, a fraction of the EBs was transferred onto gelatinized 24-well plates for alkaline phosphatase (AP) staining on day 7. Remaining EBs were allowed to differentiate in suspension and harvested on day 7 for qRT-PCR analysis of pluripotency- and differentiation-associated genes. Media were changed every other day.

Small RNA delivery

RB20 ESCs were seeded 24 h prior to transfection on gelatinized plates in ESC medium, then transfected with 100 nM of miRNA mimics (Dharmacon, miRIDIAN microRNA mimics, Thermo Fisher Scientific) or 50 nM of miRNA Power Inhibitors (Exiqon) according to the manufacturer's instructions. Prior to transfection, the miRNA mimics or the scrambled small RNA control were diluted in serum-free DMEM/F-12 (Invitrogen) and incubated with the diluted DharmaFECT1 transfection reagent (Dharmacon, Thermo Fisher Scientific) in serum-free DMEM/F-12 for 20 min at RT. miRNA inhibitors were directly added to the cell culture media without any reagent, as recommended by the manufacturer. After miRNA/anti-miRNA delivery, cells were incubated at 37°C in a humidified incubator containing 5% CO_2 prior to analysis. Cell-based and molecular assays were performed in triplicate (three biological replicates). Data are shown as the mean ± SD.

Cell viability assays

MTS proliferation assay

20 µl of the cell proliferation (MTS) reagent was directly added to the wells (96-well plate) according to the vendor's instructions (Promega) after removal of medium. Cells were incubated in a humidified incubator at 37°C for 1-3 h. Cell viability was measured by determining absorbance at 495 nm on a Multiskan MCC microplate reader (Thermo Fisher Scientific).

Live/dead viability assay

A working solution containing 0.1 µM calcein acetoxymethyl ester (calcein AM) and 0.1 µM ethidium homodimer-1 in PBS was prepared according to the instructions in the Live/Dead®

Viability/Cytotoxicity Kit for Mammalian Cells (Molecular Probes). Cells were incubated with the reagent in a humidified incubator at RT for 30-60 min. After removal of the reagent, cells were washed with PBS and viewed under fluorescence microscope (Olympus, IX71).

RNA isolation and qRT-PCR

Total RNA was extracted using either the miRVana™ miRNA Isolation Kit (Invitrogen) or miRNeasy Micro Kit (Qiagen) according to the manufacturer's instructions. NanoDrop was used to determine RNA concentration and purity. cDNA was generated from 1 μg of total RNA by reverse transcription with oligo-d T primers (Invitrogen Superscript Kit) in a 20 µl reaction. For quantitative real-time RT-PCR, the cDNA was diluted 1:10, and a total of 5 µl cDNA was used in a 25 µl PCR reaction with gene-specific primers (2.5 μM) and the Power SYBR® Green PCR Master Mix (Life Technologies). mRNA relative expression was normalized against *Gapdh* mRNA using the ΔΔC*t* method. Primer sequences are listed in the following table.

For miRNA qRT-PCR, total RNA was reverse-transcribed using specific TaqMan miRNA RT primers and amplified using miRNA-specific TaqMan® assays (Applied Biosystems). The amount of miRNA was normalized to snoRNA202. Data analysis was performed using the ΔΔC*t* method. Reactions were run on a StepOnePlus™ machine (Applied Biosystems) with three biological replicates for each of the qRT-PCR experiments.

Primers used for mRNA quantification using qRT-PCR.

**Primer**	**Primer sequence: (5' to 3')**
Oct4-F	GAAGCCGACAACAATGAGAAC
Oct4-R	ATCCTTCTCTAGCCCAAGCTG
Sox2-F	AGAACCCCAAGATGCACAAC
Sox2-R	CTCCGGGAAGCGTGTACTTA
Esrrb-F	GGCGTTCTTCAAGAGAACCA
Esrrb-R	CCCACTTTGAGGCATTTCAT
Nanog-F	CTGATTCTTCTACCAGTCCCA
Nanog-R	AAACCAGGTCTTAACCTGCTTAT
Klf5-F	CCGGAGACGATCTGAAACAC
Klf5-R	CAGATACTTCTCCATTTCACATCTTG
Gata4-F	GGTTCCCAGGCCTCTTGCAATGCGG
Gata4-R	AGTGGCATTGCTGGAGTTACCGCTG
T (Brachyury)-F	CGAGATGATTGTGACCAAGAAC
T (Brachyury)-R	GGCCTGACACATTTACCTTCA
αMHC-F	TGGTCACCAACAACCCATACGACT
αMHC-R	TGTCAGCTTGTAGACACCAGCCTT
Pax6-F	TGAATGGGCGGAGTTATGAT
Pax6-R	GGACGGGAACTGACACTC
Nestin-F	CACACCTCAAGATGTCCC
Nestin-R	GAAAGCCAAGAGAAGCCT
Map2-F	CTAAAGAACATCCGTCACAG
Map2-R	CTTCACATTACCACCTCCA
Tubb3-F	GCCTCCTCTCACAAGTATG
Tubb3-R	CCTCCGTATAGTGCCCTT
FoxA2-F	GCAGACACTTCCTACTACCAA
FoxA2-R	CTCCACTCAGCCTCTCATTT
Sox7-F	AAGGATGAGAGGAAACGTCTG
Sox7-R	ATCCACATAGGGTCTCTTCTG
Sox17-F	GATGTAAAGGTGAAAGGCGA
Sox17-R	AAGACTTGCCTAGCATCTTG
Gapdh-F	GACTTCAACAGCAACTCCCAC
Gapdh-R	TCCACCACCCTGTTGCTGTA
Nkx2.5-F	CCTCGGGCGGATAAAAAAGAGC
Nkx2.5-R	TAGCGACGGTTCTGGAACCA
CK18-F	ATTTCAGTCTCAACGATGCC
CK18-R	GAACTCTGGTGTCATTAGTCTC
Rex1-F	TAGCCGCCTAGATTTCCACT
Rex1-R	GTCCATTTCTCTAATGCCCAC
Fgf5-F	CAAGTGCCAAATTTACGGATGA

Fgf5-R	GAACAGTGACGGTGAAGGAA
Map4-F	GGAGTGGCTTTCTTCCCAAGCTG
Map4-R	GGCCCTAAGATCAACATCAAGGAGG
Rbm26-F	GATGCAGGCTGGAGAAGAAGTCAC
Rbm26-R	CCGTGAGCTGTACCTCGACCTC
Idh2-F	CCGCCATTACCGAGAACACCAG
Idh2-R	AGTGCTCGTTCAGCTTCACATTGC
Srsf1-F	ACCCATACTGACAGTTGATACCAC
Srsf1-R	CTGGGAGGAAGAGAGGAACATTGC
Pura-F	GGCACCTCCTTGACTGTGGAC
Pura-R	AGAAGGTGTGTCCGAACTTGG
Myl12b-F	GAAGCCACAGGCACCATCCAG
Myl12b-R	GCCTCTCTGTACAGCTCATCCAC
Cbx3-F	AGATGCTGCTGACAAACCAAGG
Cbx3-R	AAGGCAATGACAATCTGAGGACAC
Pcbp2-F	TCGGCAGGTTACCATCACTGGATC
Pcbp2-R	TGGATGGGTCTGCTCTGTTCTAGC
Srsf11-F	GCCTGCAGAATCGTCACCAGTACC
Srsf11-R	GCCACCCAACACTAGCCATCTACC
P4ha1-F	TGAGCGATGTGTCTGCTGGAG
P4ha1-R	CACGGCCTTCGAAATTCTTGTCC
Prkag1-F	CCGAGTCTCCGCCTTACCTGTAG
Prkag1-R	CCACCAGACGGTGAACCTCTGC
Acat1-F	CACGGAGGAGCTGTTTCTCTGG
Acat1-R	CAGCCGGTCACATGGAACTGTC
Cpt1a-F	AGCCATGGAGGTTGTCCACGAG
Cpt1a-R	GTCCATCATGGCTTGTCTCAAGTG
Myo1b-F	CCGAGGTCCCCCTGGTAGATG
Myo1b-R	GAGAGTCGTGCGATACAGCTTGG
Ero1l-F	AGCGGACCAAGTTATGAGTTCCAG
Ero1l-R	GCACATTCCAACCGTCCTCCTCAG
Mrpl15-F	GAGGGCAGACTCCATTTTACATACG
Mrpl15-R	GGCAGCAATGGCTAATTCTGAAGC
Sbno1-F	CCTCTAGCATCTTCAGTCAGTCAG
Sbno1-R	CCAGGCTATGAGCTACTGTCTACC
Cdv3-F	CCTGTCAGTTACCTACCTTGTCAC
Cdv3-R	CTCCACCTCCTGCTTCCTACACTG

Cell cycle analysis

ESCs (initially seeded at 2.0×10^5 cells/well in 6-well plates) were trypsinized and harvested on day 3 post-transfection, then washed once at RT in 1X PBS. While vortexing, ice-cold 70% ethanol was added to the cells drop by drop in order to fix the cells. Next, the cells were incubated at -20˚C for at least 2 h, then washed twice with ice-cold PBS in order to remove the ethanol. For propidium iodide (PI)/RNase staining, the cells were resuspended in PI/RNase Staining Buffer (100 μg/ml RNase and 12.5 μg/ml PI) and incubated at RT (or 37˚C) for 15-30 min in the dark. Flow cytometry was performed using a BD LSR II flow cytometer (BD Biosciences) and the data were analyzed with BD FACSDiva (BD Biosciences). The experiments were performed in triplicate.

Alkaline phosphatase (AP) staining and colony formation assay

AP staining was performed using a Leukocyte Alkaline Phosphatase Kit (Sigma-Aldrich) according to the vendor's instructions. Briefly, cells were washed with PBS, fixed with 4% paraformaldehyde, washed with deionized water, stained for 15 min at RT in the dark, washed again with PBS, and then suspended in PBS for storage.

miRNA mimics were transfected into ESCs 24 h after plating (6.0×10^4 cells/well of 12-well plates) and 3 days later the cells were trypsinized and replated at 5.0×10^3 cells/well on gelatin-coated 24-well plates. AP activity was determined 5 days after replating. Differentiated (AP-negative) and undifferentiated (AP-positive) colonies were counted to determine clonogenicity.

Analysis of DNA methylation

To analyze the high-throughput methylation data of serum and 2i cells, the pre-processed whole-genome bisulfite sequencing data of the mESCs cultured in 2i and serum conditions were

obtained from GSE56879 (Angermueller et al., 2016). From all CpGs, we kept only those with minimum sequencing coverage of 5 reads in both 2i and serum conditions. Methylation ratio of each condition and the difference between conditions were computed using custom R script. The results were visualized using IGV version 2.3.67 (Robinson et al., 2011).

To analyze DNA methylation using methylation-sensitive restriction enzyme digestion followed by qPCR (MSRE-qPCR), the methylation level of a selected set of CpG dinucleotides within the three DMRs of the *Dlk1-Dio3* locus was determined by restriction enzyme digestion and real-time PCR analysis using specific primers designed to amplify genomic regions flanking specific restriction sites within the *Dlk1-Dio3* locus. Two methylation-sensitive enzymes HhaI and HpaII (NEB, UK) were used to digest the DNA. Preparation of DNA templates, enzymatic digestion and real-time PCR analysis were performed according to Oakes and colleagues (Oakes et al., 2006). Briefly, DNA was extracted from 2i, R2i and serum cells using DNeasy Blood & Tissue Kit (Qiagen, USA). 2µg DNA (50 ng/µl) was digested by HhaI or HpaII. A mock digestion with no enzyme (sham) was performed for each sample. In order to perform qPCR, the *Dlk1-Dio3* region (GeneBank ID: AJ320506.1) was used as template and specific primer pairs were designed to amplify the flanking fragments of either HhaI or HpaII restriction sites (see Table below). A control primer pair (F: GGTGCCAGCAGAGACTTACACAG and R: CATGCCCTTTGACACTTAGTATGC) was designed for *β2m* gene to amplify a region that is devoid of the restriction sites of HpaII and HhaI. Real-time PCR was performed using a StepOnePlus instrument (Thermofisher, USA) in a final reaction volume of 20 µl with 12.5 ng of digested template, 10 µl SYBR green master mix 2X and 200 nM of each forward and reverse primers. The thermal reaction condition was as follows: an initial denaturation at 95 °C for 10

min, followed by 40 cycles of denaturation at 95 °C for 10 s, annealing at 60 °C for 20 s and extension at 72 °C for 20 s. All reactions were run in triplicate.

Primers used for DNA methylation analysis.

DMR	CpG Position in DMR (AJ320506.1)	Primer pair	Enzyme
	12062	F: 5'-AGCTGCAATGCTCATTCCTAGTG-3'<br>R: 5'-TAGTGGTCTATTCACCCAAGTGC-3'	HpaII
	12678	F: 5'-GGAAAGGGCATGGGAGAGGAC-3'<br>R: 5'-CCATCGTTCTCGCATGGGTTAGG-3'	HhaI
	13270	F: 5'-AGGCCATCTGCTTCACCATCC-3'<br>R: 5'-CGCTGTTATACTGCAACAGGAG-3'	HhaI
	14538	F: 5'-GCCCAAGACTCCACCTCATGC-3'<br>R: 5'-CACCCCACAAGCCATAGTGTC-3'	HpaII
	79652	F: 5'- GGATCCTGACCTATGTGTACCTCTG-3'<br>R: 5'- ACGGACCGTGTGTATGTGCTGTAG-3'	HhaI
	79723	F: 5'- GGATCCTGACCTATGTGTACCTCTG-3'<br>R: 5'- ACGGACCGTGTGTATGTGCTGTAG-3'	HpaII
	80437	F: 5'-TCTTGTGGCAAAGGTACGTGACTG-3'<br>R: 5'-GTATGCTATGCATTTGTGCTGAAGG-3'	HpaII
	81915	F: 5'-AGCTGACTTCCTTCAGCCACAGT-3'<br>R: 5'-TTGACCCTGTGAGAGATGCTCAG-3'	HpaII
	81915	F: 5'-AGCTGACTTCCTTCAGCCACAGT-3'<br>R: 5'-TTGACCCTGTGAGAGATGCTCAG-3'	HpaII
	85733	F: 5'- AGTTTCTGGGAAACGTACAGAAGG-3'<br>R: 5'- CACTTCTCTGCAAGGCCAAGTC-3'	HhaI
	95372	F: 5'-GGTCGGGAGCGAGATGGGTTG-3'<br>R: 5'-GCGTCCATGACACCCTAAATCAC-3'	HhaI
	97768	F: 5'-TCCCGTTCATGGCTCATGTGTCTC-3'<br>F: 5'-GCCCTGGAAATGACCGCACACTC-3'	HhaI
	97800	F: 5'-TCCCGTTCATGGCTCATGTGTCTC-3'<br>F: 5'-GCCCTGGAAATGACCGCACACTC-3'	HpaII
	99823	F: 5'-CCCTCTCAGTTTCCCAAACCTG-3'<br>R: 5'-CCAAGGTATCCTGGAAGAGCTGAC-3'	HpaII

Small RNA deep sequencing

Total RNA was extracted from the mESC samples and used to generate small RNA libraries from which cDNA libraries were prepared at LC Sciences. The purified cDNA libraries were

then used for cluster generation on Illumina's Cluster Station and then sequenced on Illumina GAIIx (Illumina HiSeq 1000/Illumina GAIIx) according to the vendor's instruction for running the instrument. Raw sequencing reads (up to 45 nt) were obtained using Illumina's Sequencing Control Studio software version 2.8 following real-time sequencing image analysis and base-calling by Illumina's Real-Time Analysis version 1.8.70 (LC Sciences).

Bioinformatics and statistical analyses

In order to determine the expression levels of miRNAs across the samples, we used miARma-Seq pipeline for raw data analysis. Briefly, the pre-processed sequences were subjected to quality assessment using FastQC v0.11.5. The minimum observed Phred score across all samples and all bases was 30 (Sanger/Illumina 1.9 encoding), which depicted the high quality of sequencing procedure. Cutadapt v1.3 (Creighton et al., 2008) was used to trim the adapter sequences (TruSeq small RNA 3' adapter TGGAATTCTCGGGTGCCAAGG and small RNA sequencing 5' primer TTCAGAGTTCTACAGTCCGACGATC according to Illumina Adapter Sequences manual v01, Feb 2016) and remove the processed sequences shorter than 15 or longer than 35 bases. A second quality control was performed after the adapter trimming using FastQC. We then used bowtie v1.1.2 (Langmead et al., 2009) to align the processed reads against *Mus musculus* genome assembly mm10 (GRCm38) obtained from the UCSC Genome Bioinformatics. The location of the miRNAs and other small non-coding RNAs was processed from the miRBase genome coordinates mmu.gff3 v21. The number of reads that were mapped to the genomic location of each mature miRNA was counted using the featureCounts v1.5.0-p1 (Liao et al., 2014).

Cross-sample normalization and differential expression analysis were performed based on a Negative Binomial distribution analysis by R/Bioconductor package DESeq2 (Love et al., 2014). The miRNAs with the absolute log2-fold change greater than 1 and the adjusted *p*-values less than 0.01 (Bonferroni-Hochberg) were considered significantly differentially expressed. Principal Components Analysis (PCA), pairwise Pearson correlation coefficients heatmaps, and visualization of the results was performed by a custom R script using several packages such as ggplot2, pheatmap, plyr, reshape2, and gplots. Target prediction analysis was performed by miRGate database (Andres-Leon et al., 2015). The predicted targets that were reported in the TargetScan database (Lewis et al., 2005) were considered for further analysis. The gene ontology (GO) analysis was done by the DAVID gene annotation tool v6.8 (Huang da et al., 2009) and Enrichr (Kuleshov et al., 2016). We also used GraphPad Prism v5.0 to visualize some of the results.

For all assays, n represents the number of independent experiments.

III. Supplemental References

Andres-Leon, E., Gonzalez Pena, D., Gomez-Lopez, G., and Pisano, D.G. (2015). miRGate: a curated database of human, mouse and rat miRNA-mRNA targets. Database (Oxford) bav035.

Angermueller, C., Clark, S.J., Lee, H.J., Macaulay, I.C., Teng, M.J., Hu, T.X., Krueger, F., Smallwood, S.A., Ponting, C.P., Voet, T., *et al.* (2016). Parallel single-cell sequencing links transcriptional and epigenetic heterogeneity. Nat. Methods *13*, 229-232.

Creighton, C.J., Nagaraja, A.K., Hanash, S.M., Matzuk, M.M., and Gunaratne, P.H. (2008). A bioinformatics tool for linking gene expression profiling results with public databases of microRNA target predictions. RNA *14*, 2290-2296.

Huang da, W., Sherman, B.T., and Lempicki, R.A. (2009). Systematic and integrative analysis of large gene lists using DAVID bioinformatics resources. Nat. Protocols *4*, 44-57.

Kuleshov, M.V., Jones, M.R., Rouillard, A.D., Fernandez, N.F., Duan, Q., Wang, Z., Koplev, S., Jenkins, S.L., Jagodnik, K.M., Lachmann, A., *et al.* (2016). Enrichr: a comprehensive gene set enrichment analysis web server 2016 update. Nucleic Acids Res. *44*, W90-97.

Langmead, B., Trapnell, C., Pop, M., and Salzberg, S.L. (2009). Ultrafast and memory-efficient alignment of short DNA sequences to the human genome. Genome Biol. *10*, R25.

Lewis, B.P., Burge, C.B., and Bartel, D.P. (2005). Conserved seed pairing, often flanked by adenosines, indicates that thousands of human genes are microRNA targets. Cell *120*, 15-20.

Liao, Y., Smyth, G.K., and Shi, W. (2014). featureCounts: an efficient general purpose program for assigning sequence reads to genomic features. Bioinformatics *30*, 923-930.

Love, M.I., Huber, W., and Anders, S. (2014). Moderated estimation of fold change and dispersion for RNA-seq data with DESeq2. Genome Biol. *15*, 550.

Oakes, C.C., La Salle, S., Robaire, B., and Trasler, J.M. (2006). Evaluation of a quantitative DNA methylation analysis technique using methylation-sensitive/dependent restriction enzymes and real-time PCR. Epigenetics *1*, 146-152.

Robinson, J.T., Thorvaldsdottir, H., Winckler, W., Guttman, M., Lander, E.S., Getz, G., and Mesirov, J.P. (2011). Integrative genomics viewer. Nat. Biotechnol. *29*, 24-26.
